# Sharing interoperable workflow provenance: A review of best practices and their practical application in CWLProv

**DOI:** 10.1093/gigascience/giz095

**Published:** 2019-11-01

**Authors:** Farah Zaib Khan, Stian Soiland-Reyes, Richard O Sinnott, Andrew Lonie, Carole Goble, Michael R Crusoe

**Affiliations:** 1 The University of Melbourne, School of Computing and Information System, Doug Mcdonnell Building, Parkville, Australia, 3052; 2 Common Workflow Language Project; 3 The University of Manchester, UK

**Keywords:** provenance, Common Workflow Language, CWL, Research Object, RO, BagIt, interoperability, scientific workflows, containers

## Abstract

**Background:**

The automation of data analysis in the form of *scientific workflows* has become a widely adopted practice in many fields of research. Computationally driven data-intensive experiments using workflows enable automation, scaling, adaptation, and provenance support. However, there are still several challenges associated with the effective sharing, publication, and reproducibility of such workflows due to the incomplete capture of provenance and lack of interoperability between different technical (software) platforms.

**Results:**

Based on best-practice recommendations identified from the literature on workflow design, sharing, and publishing, we define a hierarchical provenance framework to achieve uniformity in provenance and support comprehensive and fully re-executable workflows equipped with domain-specific information. To realize this framework, we present CWLProv, a standard-based format to represent any workflow-based computational analysis to produce workflow output artefacts that satisfy the various levels of provenance. We use open source community-driven standards, interoperable workflow definitions in Common Workflow Language (CWL), structured provenance representation using the W3C PROV model, and resource aggregation and sharing as workflow-centric research objects generated along with the final outputs of a given workflow enactment. We demonstrate the utility of this approach through a practical implementation of CWLProv and evaluation using real-life genomic workflows developed by independent groups.

**Conclusions:**

The underlying principles of the standards utilized by CWLProv enable semantically rich and executable research objects that capture computational workflows with retrospective provenance such that any platform supporting CWL will be able to understand the analysis, reuse the methods for partial reruns, or reproduce the analysis to validate the published findings.

## Introduction

Amongst the many big data domains, genomics is considered *the most demanding* with respect to all stages of the data lifecycle, including acquisition, storage, distribution, and analysis [1]. Because genomic data are growing at an unprecedented rate due to improved sequencing technologies and reduced cost, it is currently challenging to analyse the data at a rate matching their production. With data growing exponentially in size and volume, the practice of performing computational analyses using *workflows* has overtaken more traditional research methods using ad hoc scripts, which were the typical modus operandi over the past few decades [2,3]. Scientific workflow design and management has become an essential part of many computationally driven data-intensive analyses enabling automation, scaling, adaptation, and provenance support [4]. Increased use of workflows has driven rapid growth in the number of computational data analysis workflow management systems (WMSs), with hundreds of heterogeneous approaches now existing for workflow specification and execution [5]. There is an urgent need for a common format and standard to define workflows and enable sharing of analysis results using a given workflow environment.

Common Workflow Language (CWL) [6] has emerged as a workflow definition standard designed to enable portability, interoperability, and reproducibility of analyses between workflow platforms. CWL has been widely adopted by >20 organizations, providing an interoperable bridge overcoming the heterogeneity of workflow environments. Whilst a common standard for workflow definition is an important step towards interoperable solutions for workflow specifications, sharing and publishing the *results* of these workflow enactments in a common format is equally important. Transparent and comprehensive sharing of experimental designs is critical to establish trust and ensure authenticity, quality, and reproducibility of any workflow-based research result. Currently there is no common format defined and agreed upon for interoperable workflow archiving or sharing [7].

In this paper, we utilize open source standards such as CWL together with related efforts such as research objects (ROs) [8], BagIt [9], and PROV [10] to define CWLProv, a format for the interoperable representation of a CWL workflow enactment. We focus on production of a workflow-centric executable RO as the final result of a given CWL workflow enactment. This RO is equipped with the artefacts used in a given execution including the workflow inputs, outputs, and, most importantly, the retrospective provenance. This approach enables the complete sharing of a computational analysis such that any future CWL-based workflow can be rerun given the best practices discussed later for software environment provision are followed.

The concept of workflow-centric ROs has been previously considered [8, 11,12] for structuring the analysis methods and aggregating digital resources used in a given analysis. The ROs generated in these studies typically aggregate data objects, example inputs, workflow specifications, attribution details, and details about the execution environment amongst various other elements. These previous efforts were largely tied to a single platform or WMS. CWLProv aims to provide a platform-independent solution for workflow sharing, enactment, and publication. All the standards and vocabularies used to design CWLProv have an overarching goal to support a domain-neutral and interoperable solution (detailed in Section **Applied Standards and Vocabularies**).

The remainder of this paper is structured as follows. In Section **Background** we discuss the key concepts and related work, followed by a summary of the published best practices and recommendations for workflow representation and sharing in Section **Levels of Provenance and Resource Sharing**. This section also details the hierarchical provenance framework that we define to provide a principled approach for provenance capture and method sharing. Section **CWLProv 0.6.0 and Utilized Standards** introduces CWLProv and outlines its format, structure, and the details of the standards and ontologies it utilizes. Section **Practical Realization of CWLProv** presents the implementation details of CWLProv using cwltool [13], and Section **CWLProv Evaluation with Bioinformatics Workflows** demonstrates and evaluates the implemented module for 3 existing workflow case studies. We discuss the challenges of interoperable workflow sharing and the limitations of the proposed solution, listing several possible future research directions, in Section **Discussion and Future Directions** before finally drawing conclusions in Section**Conclusion**.

## Background

This work draws upon a range of topics such as “*provenance*" and “*interoperability*." We define these here to provide better context for the reader.

### Provenance

A number of studies have advocated for complete provenance tracking of scientific workflows to ensure transparency, reproducibility, analytic validity, quality assurance, and attribution of (published) research results [14]. The term *“provenance"* is defined by the World Wide Web Consortium (W3C) [15] as:

*“Provenance is information about entities, activities, and people involved in producing a piece of data or thing, which can be used to form assessments about its quality, reliability, or trustworthiness."*

Provenance for workflows is commonly divided into the following 3 categories: *retrospective provenance, prospective provenance*, and *workflow evolution. “Retrospective provenance"* refers to the detailed record of the implementation of a computational task including the details of every executed process together with comprehensive information about the execution environment used to derive a specific product. *“Prospective provenance"* refers to the “recipes" used to capture a set of computational tasks and their order, e.g., the workflow specification [16]. This is typically given as an abstract representation of the steps (tools/data analysis steps) that are necessary to create a particular research output, e.g., a data artefact. *“Workflow evolution"* refers to tracking of any alteration in the existing workflow resulting in another version of the workflow that may produce either the same or different resultant data artefacts [17]. In this work, our focus is mainly on improving representation and capture of *retrospective provenance*.

### Interoperability

The concept of interoperability varies in different domains. Here we focus on *computational interoperability*, defined as:

*“The ability of 2 or more components or systems to exchange information and to use the information that has been exchanged* [18].*"*

The focus of this study is to propose and devise methods to achieve *syntactic, semantic*, and *pragmatic* interoperability as defined in the Levels of Conceptual Interoperability Model [19]. *Syntactic* interoperability is achieved when a common data format for information exchange is unambiguously defined. The next level of interoperability, referred to as *semantic* interoperability, is reached when the content of the actual information exchanged is unambiguously defined. Once there is agreement about the format and content of the information, *pragmatic* interoperability is achieved when the context, application, and use of the shared information and data exchanged are also unambiguously defined. In the section **Evaluation Results**, we relate these general definitions to specific workflow applications with respect to workflow-centric ROs and describe to what extent these interoperability requirements are addressed.

### Related work

We focus on relevant studies and efforts trying to resolve the issue of availability of required resources used in a given computational analysis. In addition, we cover efforts directed towards provenance capture of workflow enactments. We restrict our attention to scientific workflows and studies related to the bioinformatics domain.

#### Workflow software environment capture

*Freezing* and packaging the runtime environment to encompass all the software components and their dependencies used in an analysis is a recommended and widely adopted practice [20] especially after use of cloud computing resources where images and snapshots of the cloud instances are created and shared with fellow researchers [21]. Nowadays, preservation and sharing of the software environment, e.g., in open access repositories, is becoming a regular practice in the workflow domain as well. Leading platforms managing infrastructure and providing cloud computing services and configuration on demand include DigitalOcean [22], Amazon Elastic Compute Cloud [23], Google Cloud Platform [24], and Microsoft Azure [25]. The instances launched on these platforms can be saved as snapshots and published with an analysis study to later recreate an instance representing the computing state at analysis time.

Using *“system-wide packaging"* for data-driven analyses, although simplest on the part of the workflow developers and researchers, has its own caveats. One notable issue is the size of the snapshot as it captures everything in an instance at a given time; hence, the size can range from a few gigabytes to many terabytes. To distribute research software and share execution environments, various lightweight and container-based virtualization and package managers are emerging, including Docker, Singularity, Debian Med, and Bioconda.

*Docker* [26] is a lightweight container-based virtualization technology that facilitates the automation of application development by archiving software systems and environment to improve portability of the applications on many common platforms including Linux, Microsoft Windows, Mac OS X, and cloud instances. *Singularity* [27] is also a cross-platform open source container engine specifically supporting high-performance computing resources. An existing Docker format software image can be imported and used by the Singularity container engine. *Debian Med* [28] contributes packages of medical practice and biomedical research to the Debian Linux distribution, lately also including workflows. *Bioconda* [29] packages, based on the open source package manager Conda [30], are available for Mac OS X and Linux environments, directing towards availability and portability of software used in the life sciences domain.

#### Data/method preservation, aggregation, and sharing

Preserving and sharing only the software environment is not enough to verify results of any computational analysis or reuse the methods (e.g., workflows) with a different dataset. It is also necessary to share other details including data (example or the original), scripts, workflow files, input configuration settings, the hypothesis of the experiment, and any/all trace/logging information related to “what happened," i.e., the retrospective provenance of the actual workflow enactment. The publishing of resources to improve the state of scholarly publications is now supported by various online repositories, including Zenodo [31], GitHub [32], myExperiment [33], and Figshare [34]. These repositories facilitate collaborative research, in addition to public sharing of source code and the results of a given analysis. There is however no standard format that must be followed when someone shares artefacts associated with an analysis. As a result, the quality of the shared resources can range from a highly annotated, properly documented and complete set of artefacts to raw data with undocumented code and incomplete information about the analysis as a whole. Individual organizations or groups might provide a set of “recommended practices," e.g., in readme files, to attempt to maintain the quality of shared resources. The initiative *Code as a Research Object* [35] is a joint project between Figshare, GitHub, and Mozilla Science Lab [36] and aims to archive any GitHub code repository to Figshare and produce a DOI to improve the discovery of resources (for the source code that supports this work we have used a similar publishing feature with Zenodo).

ReproZip [37] aims to resolve portability issues by identifying and packaging all dependencies in a self-contained package that when unpacked and executed on another system (with ReproZip installed) should reproduce the methods and results of the analysis. Each package also contains a human-readable configuration file containing provenance information obtained by tracing system calls during system execution. The corresponding provenance trace is however not formatted using existing open standards established by the community. Several platform-dependent studies have been targeted towards extensions to existing standards by implementing the RO model and improving aggregation of resources. Belhajjame et al. [8] proposed the application of ROs to develop workflow-centric ROs containing data and metadata to support the understandability of the utilized methods (in this case workflow specifications). They explored 5 essential requirements to workflow preservation and identified data and metadata that could be stored to satisfy the said requirements. These requirements include providing example data, preserving workflows with provenance traces, annotating workflows, tracking the evolution in workflows, and packaging the auxiliary data and information with workflows. They proposed extensions to existing ontologies such as Object Reuse and Exchange (ORE), the Annotation Ontology (AO), and PROV-O, with 4 additional ontologies to represent workflow-specific information. However, as they state, the scope of the proposed model at that time was not focused on interoperability of heterogeneous workflows because it was demonstrated for a workflow specific to Taverna WMS using myExperiment, which makes it quite platform-dependent.

A domain-specific solution was proposed by Gomez-Perez et al. [38] by extending the RO model to equip workflow-centric ROs with information catering to the specific needs of the earth science community, resulting in enhanced discovery and reusability by experts. They demonstrated that the principles of ROs can support extensions to generate aggregated resources leveraging domain-specific knowledge. Hettne et al. [11] used 3 genomic workflow case studies to demonstrate the use of ROs to capture methods and data supporting querying and useful extraction of information about the scientific investigation under observation. The solution was tightly coupled with the Taverna WMS and hence, if shared, would not be reproducible outside of the Taverna environment. Other notable efforts to use ROs for workflow preservation and method aggregation have been undertaken in systems biology [39], in clinical settings [40], and in precision medicine [41].

#### Provenance capture and standardization

A range of standards for provenance representation have been proposed. Many studies have emphasized the need for provenance focusing on aspects such as scalability, granularity, security, authenticity, modelling, and annotation [14]. They identify the need to support standardized dialogues to make provenance interoperable. Many of these were used as inputs to initial attempts at creating a standard Provenance Model to tackle the often inconsistent and disjointed terminology related to provenance concepts. This ultimately resulted in the specification of the *Open Provenance Model* (OPM) [42] together with an open source model for the governance of OPM [43]. Working towards similar goals of interoperability and standardization of provenance for web technologies, the W3C Provenance Incubator Group [44] and the authors of OPM together set the fourth provenance challenge at the International Provenance and Annotation Workshop, 2010 (IPAW’10), that later resulted in PROV, a family of documents serving as the conceptual model for provenance capture and its representation, sharing, and exchange over the Web [45] regardless of the domain or platform. Since then, a number of studies have proposed extensions to this domain-neutral standard. The model is general enough to be adapted to any field and flexible enough to allow extensions for specialized cases.

Michaelides et al. [46] presented a domain-specific PROV-based solution for retrospective provenance to support portability and reproducibility of a statistical software suite. They captured the essential elements from the log of a workflow enactment and represented them using an intermediate notation. This representation was later translated to PROV-N and used as the basis for the PROV Template System. A Linux-specific system provenance approach was proposed by Pasquier et al. [47], who demonstrated retrospective provenance capture at the system level. Another project, *UniProv*, is working to extract information from Unicore middleware and transform it into a PROV-O representation to facilitate the backtracking of workflow enactments [48]. Other notable domain-specific efforts leveraging the established standards to record provenance and context information are *PROV-man* [49], PoeM [50], and micropublications [51]. Platforms such as VisTrails and Taverna have built in retrospective provenance support. *Taverna* [39] implements an extensive provenance capture system, *TavernaProv* [52], using both PROV ontologies as well as ROs aggregating the resources used in an analysis. *VisTrails* [53] is an open source project supporting platform-dependent provenance capture, visualization, and querying for extraction of required information about a workflow enactment. Chirigati et al. [37] provide an overview of PROV terms and how they can be translated from the VisTrails schema and serialized to PROV-XML. *WINGS* [54] can report fine-grained workflow execution provenance as Linked Data using the Open Provenance Model for Workflows ontology [55], which builds on both PROV-O and OPM.

All these efforts are fairly recent and use a standardized approach to provenance capture and hence are relevant to our work on the capture of retrospective provenance. However, our aim is a domain-neutral and platform-independent solution that can be easily adapted for any domain and shared across different platforms and operating systems.

As evident from the literature, there are efforts in progress to resolve the issues associated with effective and complete sharing of computational analysis including both the results and provenance information. These studies range from highly domain-specific solutions and platform-dependent objects to open source flexible interoperable standards. CWL has widespread adoption as a workflow definition standard and hence is an ideal candidate for portable workflow definitions. The next section investigates existing studies focused on workflow-centric science and summarizes best-practice recommendations put forward in these studies. From this we define a hierarchical provenance and resource-sharing framework.

## Levels of Provenance and Resource Sharing

**Figure 1:**
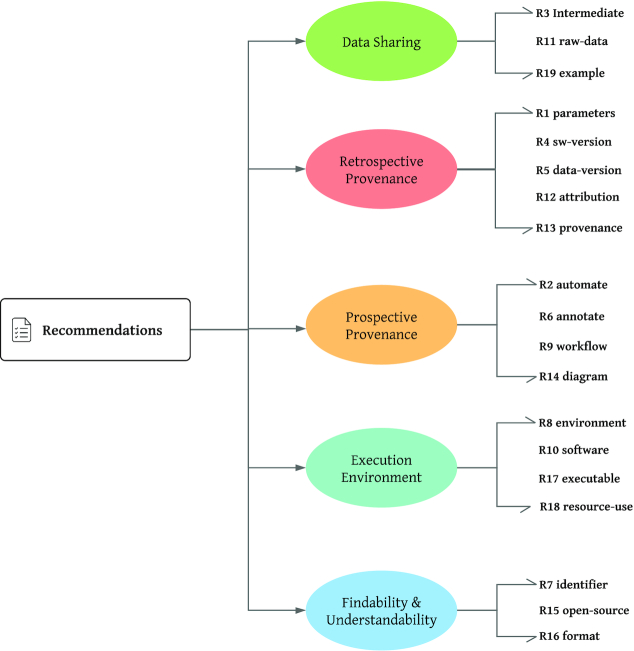
Recommendations from Table 1 classified into these categories.

Various studies have empirically investigated the role of automated computational methods in the form of workflows and published best-practice recommendations to support workflow design, preservation, understandability, and reuse. We summarize a number of these recommendations and their justifications in Table 1, where each recommendation addresses a specific requirement of workflow design and sharing. These recommendations can be clustered into broad themes as shown in Fig. 1. This classification can be made in >1 way, e.g., according to how these recommendations are supporting each FAIR dimension (Findable, Accessible, Interoperable, and Reusable) [56]. In this study, we have focused on categories with respect to workflow design, prospective provenance, data sharing, retrospective provenance, the computational environment required/used for an analysis, and better findability and understandability of all shared resources.

**Table 1.** Summarized recommendations and justifications from the literature covering best practices on reproducibility, accessibility, interoperability, and portability of workflows

R No.	Recommendations	Justifications
R1-parameters	Save and share all parameters used for each software executed in a given workflow (including default values of parameters used) [55, 57–59].	Affects reproducibility of results because different inputs and configurations of the software can produce different results. Different versions of a tool might upgrade the default values of the parameters.
R2-automate	Avoid manual processing of data, and if using shims [60], then make these part of the workflow to fully automate the computational process [57, 59].	This ensures the complete capture of the computational process without broken links so that the analysis can be executed without the need for performing manual steps.
R3-intermediate	Include intermediate results where possible when publishing an analysis [55, 58, 59].	Intermediate data products can be used to inspect and understand shared analysis when re-enactment is not possible.
R4-sw-version	Record the exact software versions used [57, 59].	This is necessary for reproducibility of results because different software versions can produce different results.
R5-data-version	If using public data (reference data, variant databases), then it is necessary to store and share the actual data versions used [3, 61, 57, 59].	This is needed because different versions of data, e.g., human reference genome or variant databases, can result in slightly different results for the same workflow.
R6-annotation	Workflows should be well-described, annotated, and offer associated metadata. Annotations such as user-contributed tags and versions should be assigned to workflows and shared when publishing the workflows and associated results [8, 12, 55, 62, 63].	Metadata and annotations improve the understandability of the workflow, facilitate independent reuse by someone skilled in the field, make workflows more accessible, and hence promote the longevity of the workflows.
R7-identifier	Use and store stable identifiers for all artefacts including the workflow, the datasets, and the software components [62, 63].	Identifiers play an important role in the discovery, citation, and accessibility of resources made available in open access repositories.
R8-environment	Share the details of the computational environment [8, 61, 63].	Such details support analysis of requirements before any re-enactment or reproducibility is attempted.
R9-workflow	Share workflow specifications/descriptions used in the analysis [8, 55, 58, 63, 64].	The same workflow specifications can be used with different datasets, thereby supporting reusability.
R10-software	Aggregate the software with the analysis and share this when publishing a given analysis [61, 63, 64].	Making software available reduces dependence on third-party resources and as a result minimizes “workflow decay" [65].
R11-raw-data	Share raw data used in the analysis [8, 55, 58, 63, 64].	When someone wants to validate published results, availability of data supports verification of claims and hence establishes trust in the published analysis.
R12-attribution	Store all attributions related to data resources and software systems used [55,64].	Accreditation supports proper citation of resources used.
R13-provenance	Workflows should be preserved along with the provenance trace of the data and results [8, 12, 55, 59, 64].	A provenance trace provides a historical view of the workflow enactment, enabling end users to better understand the analysis retrospectively.
R14-diagram	Data flow diagrams of the computational analysis using workflows should be provided [58, 61].	These diagrams are easy to understand and provide a human-readable view of the workflow.
R15-open-source	Open source licensing for methods, software, code, workflows, and data should be adopted instead of proprietary resources [58, 59, 63, 64, 66].	This improves availability and legal reuse of the resources used in the original analysis, while restricted licenses would hinder reproducibility.
R16-format	Data, code, and all workflow steps should be shared in a format that others can easily understand, preferably in a system-neutral language [8, 58, 66].	System-neutral languages help achieve interoperability and make an analysis understandable.
R17-executable	Promote easy execution of workflows without making significant changes to the underlying environment [3].	In addition to helping reproducibility, this enables adapting the analysis methods to other infrastructures and improves workflow portability.
R18-resource-use	Information about compute and storage resources should be stored and shared as part of the workflow [61].	Such information can assist users in estimating the resources needed for an analysis and thereby reduce the amount of failed executions.
R19-example	Example input and sample output data should be preserved and published along with the workflow-based analysis [8, 65].	This information enables more efficient test runs of an analysis to verify and understand the methods used.

This list is not exhaustive; other studies have identified separate issues (e.g., laboratory work provenance and data security) that are beyond the scope of this work.

**Figure 2:**
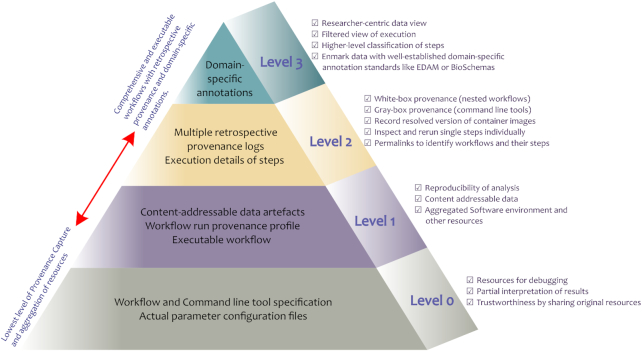
Levels of provenance and resource sharing and their applications.

Sharing “*all artefacts*” from a computational experiment (following all recommendations and best practices) is a demanding task without any informed guidance. It requires consolidated understanding of the impact of the many different artefacts involved in that analysis. This places extra efforts on workflow designers, (re)-users, authors, and reviewers and expectations on the community as a whole. Given the numerous WMSs and differences in how each system deals with provenance documentation, representation, and sharing of these artefacts, the granularity of provenance information preserved will vary for each workflow definition approach. Hence, devising 1 universal but technology-specific solution for provenance capture and the related resource sharing is impossible. Instead we propose a generic framework of provenance in Fig. 2 that all WMSs can benefit from and conform to with minimum technical overhead.

The recommendations in Table 1 aid in our understanding to define this framework by classifying the granularity of the provenance and related artefacts where the uppermost level exhibits comprehensive, reproducible, understandable, and provenance-rich computational experiment sharing. The purpose of this framework is 3-fold. First, because of its generic nature it provides uniformity in the provenance granularity across various WMSs belonging to different workflow definition approaches. Second, it provides comprehensive and well-defined guidelines that can be used by researchers to conduct principled analysis of the provenance of any published study. Third, due to its hierarchical nature, the framework can be leveraged by the workflow authors to progress incrementally towards the most transparent workflow-centric analysis. Overall, this framework will help achieve a uniform level of provenance and resource sharing with a given workflow-centric analysis guaranteed to fulfill the respective provenance applications.

Our proposed provenance levels are ordered from general to specific. In brief, Level 0 is unstructured information about the overall workflow enactment; Level 1 adds structured retrospective provenance, access to primary data, and executable workflows; Level 2 enhances the white-box provenance for individual steps; and *Level 3* adds domain-specific annotations for improved understanding. These levels are described in the following sub-sections and mapped to the requirements in Table 1 that these levels aim to satisfy.

### Level 0

To achieve this level, researchers should share the workflow specifications, input parameters used for a given workflow enactment, raw logs, and output data preferably through an open access repository. This is the least information that could be shared without exerting any extra effort to support seamless reuse or understandability of a given analysis. The artefacts shared at this level would only require uploading of the associated resources to a repository without necessarily providing any supporting metadata or provenance information. Information captured at *Level 0* is the bare minimum that can be used for result interpretation.

Workflow definitions based on *Level 0* can also potentially be repurposed for other analyses. As argued by Ludäscher, a well-written scientific workflow and its graphical representation is itself a source of prospective provenance, giving the user an idea of the steps taken and data produced [67]. Therefore, a well-described workflow specification indirectly provides prospective provenance without aiming for it. In addition to the textual workflow specification, its graphical representation should also be shared, if available, for better understandability, fulfilling *R14-diagram*. At this level, reproducing the workflow would only be possible if the end user devotes extra efforts to understand the shared artefacts and carefully recreate the execution environment. As open access journals frequently require availability of methods and data, many published studies now share workflow specifications and optionally the outputs, thereby achieving *Level 0* and specifically satisfying *R1-parameters* and *R9-workflow* (Table 1). In addition, the resources shared should have open licence starting from *Level 0*, and this practice proposed by *R15*-*open-source* should be adopted at each higher level.

### Level 1

At *Level 1, R4-sw-version,R5-data-version, R12-attribution*, and *R13-provenance* should be satisfied by providing retrospective provenance of the workflow enactment, i.e., a structured representation of machine-readable provenance that can answer questions such as “what happened," “when it happened," “what was executed," “what was used," “who did this,” and “what was produced." Seamless re-enactment of the workflow should be supported at this level. This is only possible when along with provenance information, *R8-environment* and *R10-software* are satisfied by potentially packaging the software environment for analysis sharing or there is enough information about the software environment to guide the user to reliably re-enact the workflow. Hence, *R17-executable* should be satisfied, making it possible for the end users to re-enact the shared analyses without making major changes to the underlying software environment.

In addition to the software availability and retrospective provenance, access to input data should also be provided, fulfilling *R11-raw-data*. These data can be used to re-enact the published methods or utilized in a different analysis, e.g., for performance comparison of methods. At *Level 1*, it is preferable to provide content-addressable data artefacts such as input, output, and intermediate files, avoiding local paths and file names to make a given workflow executable outside its local environment. The intermediate data artefacts should also be provided to facilitate inspection of all step results, hence satisfying *R3-intermediate*. All resources, including workflow specifications and provenance, should be shared in a format that is understandable across platforms, preferably in a technology-neutral language as proposed by *R16-format*.

While software and data can be digitally captured, the hardware and infrastructure requirements also need to be captured to fulfill *R18-resource-use*. This kind of information can naturally vary widely with runtime environments, architectures, and data sizes [68], as well as rapidly becoming outdated as hardware and cloud offerings evolve. Nevertheless a snapshot of the workflow’s overall execution resource usage for an actual run can be beneficial to give a broad overview of the requirements and can facilitate cost-efficient re-computation by taking advantage of spot-pricing for cloud resources [69].

### Level 2

It is a common practice in scientific workflows to modularize the workflow specifications by separating the related tasks into “sub-workflows” or “nested workflows” [20] to be incorporated and used in other workflows or be assigned to compute and storage resources in case of distributed computing [70]. These modular solutions promote understanding and reusability of the workflows as researchers are inclined to use these modules instead of workflow as a whole for their own computational experiments. An example of a sub-workflow is the mandatory “preprocessing” [71] needed for the Genome Analysis ToolKit (GATK) best-practice pipelines used for genomic variant calling. These steps can be separated into a sub-workflow to be used before any variant calling pipeline, be it somatic or germline.

At *Level 1*, retrospective provenance is coarse grained, and as such, there is no distinction between workflows and their sub-workflows. Ludäscher [67] distinguishes between workflow provenance as *black-box* and database provenance as *white-box*. The reasoning behind this distinction is that often the steps in a workflow, especially those based on GUI-based platforms, provide levels of abstraction/obscurity to the actual tasks being implemented. In our previous work we used an empirical case study to demonstrate that declarative approaches to workflow definition resulted in transparent workflows with the fewest assumptions [61]. This resolves the black-box/white-box issue to some extent, but to further support research transparency, we propose to share retrospective provenance logs for each nested/sub-workflow, making the details of a workflow enactment as explicit as possible and moving a step closer to *white-box* provenance. These provenance logs will support the inspection and automatic re-enactment of targeted components of a workflow such as a single step or a sub-workflow individually without necessarily having to re-enact the full analysis. Some existing make-like systems such as Snakemake support partial re-enactments but typically rely on fixed file paths for input data and require manual intervention to provide the specific directory structure. With detailed provenance logs and the corresponding content-addressable data artefacts, the partial reruns can be achieved with automatic generation of input configuration setting.

In addition, we propose to include *permalinks* at *Level 2* to identify the workflows and their individual steps, which facilitates the inspection of each step and aims to improve the longevity of the shared resources, hence supporting *R7-identifier*. Improving *R18-resource-use* for *Level* 2 would include resource usage per task execution. Along with execution times this can be useful information to identify bottlenecks in a workflow and for more complex calculations in cost optimization models [72]. At this provenance level resource usage data will however also become more noisy and highly variant on scheduling decisions by the workflow engine, e.g., sensitivity to cloud instance reuse or co-use for multiple tasks, or variation in data transfers between tasks on different instances. Thus, *Level* 2 resource usage information should be further processed with statistical models for it to be meaningful for a user keen to estimate the resource requirement for re-enactment of a given analysis.

### Level 3

Levels 0–2 are generic and domain-neutral and can apply to any scientific workflow. However, domain-specific information/metadata about data and processes plays an important role in better understanding of the analysis and exploitation of provenance information, e.g., for meaningful queries to extract information to the domain under consideration [73,74]. The addition of domain-specific metadata, e.g., file formats, user-defined tags, and other annotations to generic retrospective provenance can improve the *white-boxness* by providing domain context to the analysis as described in *R6-annotations*. Annotations can range from adding textual description and tags to marking data with more systematic and well-defined domain-specific ontologies such as EDAM [75] and BioSchemas [76] in the case of bioinformatic workflows. Some studies also propose to provide example or test data sets, which eventually helps in analyzing the methods shared and verifying their results (as described in *R19-example*).

At *Level 3*, the information from previous levels, combined with specific metadata about data artefacts, facilitates higher level classification of workflow steps into *motifs* [77] such as data retrieval, preprocessing, analysis, and visualization. This level of provenance, resource aggregation, and sharing can provide a researcher-centric view of data and enable users to re-enact a set of steps or full workflow by providing a filtered and annotated view of the execution. This can be non-trivial to achieve with mainstream methods of workflow definition and sharing because it requires guided user annotations with controlled vocabularies, but this can be simplified by reusing related tooling from existing efforts such as BioCompute Objects [41] and DataCrate [78].

Communicating resource requirements (*R18-resource-use*) at *Level 3* would involve domain-specific models for hardware use and cost prediction, as suggested for dynamic cloud costing [79] in *BioSimSpace* [80], or predicting assembler and memory settings through machine learning of variables such as source biome, sequencing platform, file size, read count, and base count in the *European Bionformatics Institute (EBI) Metagenomics* pipeline [81]. For robustness such models typically need to be derived from resource usage across multiple workflow runs with varied inputs, e.g., by a multi-user workflow platform. Taking advantage of *Level 3* resource usage models might require preprocessing workflow inputs and calculations in an environment like R or Python, so we recommend that models be provided with separate sidecar workflows for interoperable execution before the main workflow.

By explicit enumeration of the levels of provenance, it should be possible to quantify and directly assess the effort required to reuse a workflow and reproduce experiments directly. The similar effort *5-star Open Data* [82] strongly advocates open-licensed structured representation, use of stable identifiers for data sharing, and following Linked Data principles to cross-relate data. One challenge in achieving the Open Data stars is that it necessitates tool support during data processing. In our framework we proposed systematic workflow-centric resource sharing using structured Linked Data representation, including recording of the executed data operations. Hence, our effort compliments the already proposed 5-star Open Data principles and contributes to further understanding by sharing the computational method following the same principles.

Requiring researchers to achieve the above-defined levels individually is unrealistic without guidance and direct technical support. Ideally, the conceptual meaning of these levels would be translated into a practical solution utilizing the available resources. However, given the heterogeneity of workflow definition approaches, it is expected that the proposed framework, when translated into practical solutions, will result in varying workflow-centric solutions tied to specific WMSs. To support interoperability of the workflow-centric analysis achieving the provenance levels, we propose CWLProv, a format for annotating resource aggregations equipped with retrospective provenance. The next section describes CWLProv and the associated standards that are applied in this process.

## CWLProv 0.6.0 and Utilized Standards

Here we present CWLProv, a format for the methodical representation of workflow enactment, associated artefacts, and capturing and using retrospective provenance information. Keeping in view the recommendations from Table 1, e.g.,*R15-open-source* and *R16-format*, we leverage **open source, domain-independent, system-neutral**, interoperable, and most importantly **community-driven** standards as the basis for the design and formatting of reproducible and interoperable workflow-based ROs. The profile description in this section corresponds to CWLProv 0.6.0 [83] (see [84] for the latest profile).

### Applied standards and vocabularies

We follow the recommendation *“Reuse vocabularies, preferably standardized ones”* [85] from best practices associated with data sharing, representation, and publication on the Web to achieve consensus and interoperability of workflow-based analyses. Specifically we integrate CWL for workflow definition, ROs for resource aggregation, and the *PROV-Data Model* (PROV-DM) to support the retrospective provenance associated with workflow enactment. The key properties and principles of these standards are described below.

#### Common Workflow Language

CWL [6] provides declarative constructs for workflow structure and command line tool interface definition. It makes minimal assumptions about base software dependencies, configuration settings, software versions, parameter settings, or indeed the execution environment more generally [61]. The CWL object model supports comprehensive recording and capture of information for workflow design and execution. This can subsequently be published as structured information alongside any resultant analysis using that workflow.

CWL is a community-driven standard effort that has been widely adopted by many workflow design and execution platforms, supporting interoperability across a set of diverse platforms. Current adopters include Toil, Arvados, Rabix [86], Cromwell [87], REANA, and Bcbio [88], with implementations for Galaxy, Apache Taverna, and AWE currently in progress.

**Figure 3:**
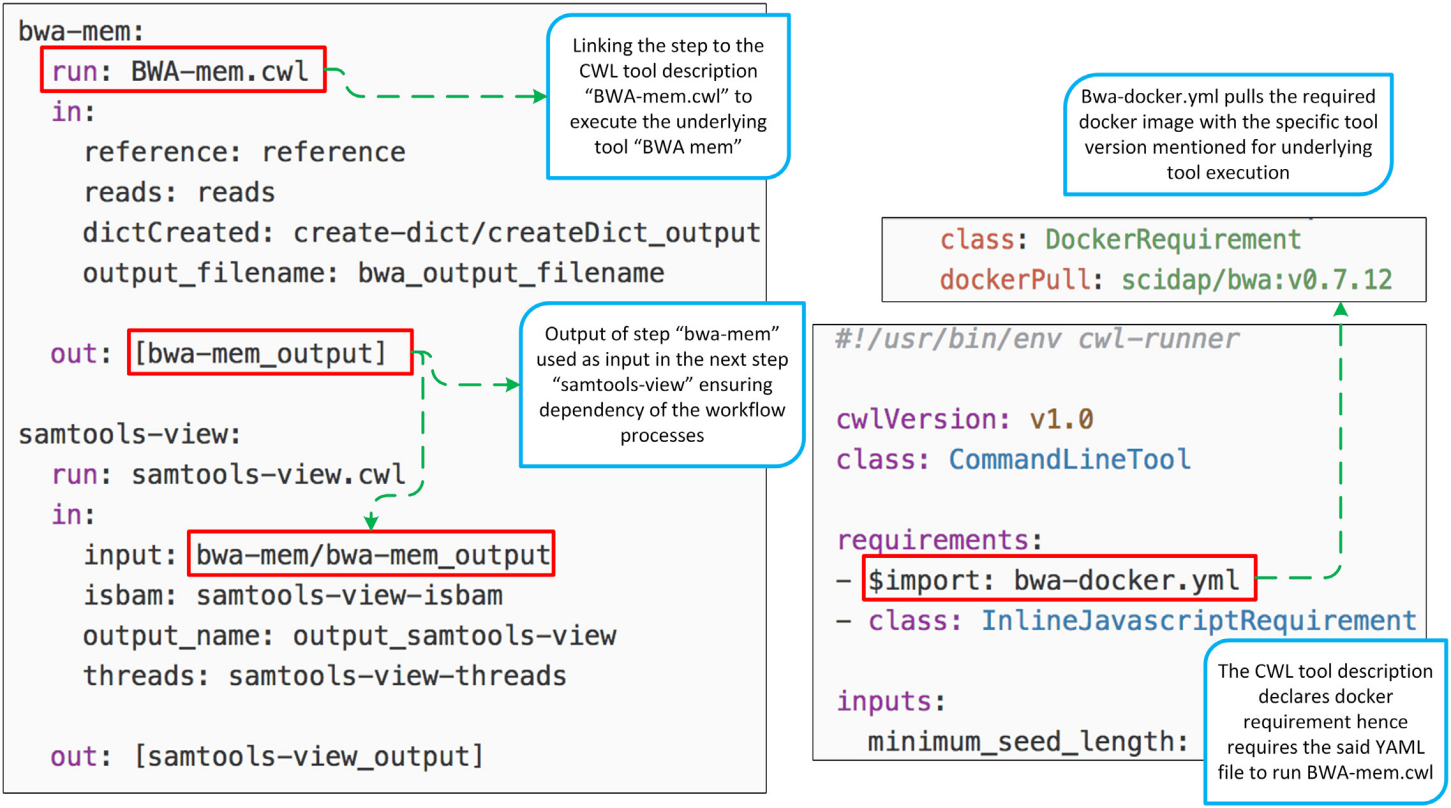
Left: A snapshot of part of a GATK workflow described using CWL. Two steps named as *“bwa-mem"* and *“samtools-view"* are shown, where the former links to the tool description executing the underlying tool (BWA-mem for alignment) and provides the output used as input for samtools. Right: Snapshot of BWA-mem.cwl and the associated Docker requirements for the exact tool version used in the workflow execution.

A workflow in CWL is composed of “steps,” where each step refers either to a command line tool (also specified using CWL) or another workflow specification incorporating the concept of “sub-workflows." Each “step” is associated with “inputs” that are composed of any data artefact required for the execution of that step (Fig. 3). As a result of the execution of each step, “outputs” are produced that can become (part of) “inputs” for the next steps, making the execution data-flow oriented. CWL is not tied to a specific operating system or platform, which makes it an ideal approach for interoperable workflow definitions.

#### Research Object

An RO encapsulates all of the digital artefacts associated with a given computational analysis contributing towards preservation of the analysis [89], together with their metadata, provenance, and identifiers.

The aggregated resources can include but are not limited to input and output data for analysis results validation, computational methods such as command line tools and workflow specifications to facilitate workflow re-enactment, attribution details regarding users, retrospective as well as prospective provenance for better understanding of workflow requirements, and machine-readable annotations related to the artefacts and the relationships between them. The goal of ROs is to make any published scientific investigation and the produced artefacts *“interoperable, reusable, citable, shareable, and portable."*

The 3 core principles [90] of the RO approach are to support “identity," “aggregation," and “annotation” of research artefacts. They look to enable accessibility of tightly coupled, interrelated, and well-understood aggregated resources involved in a computational analysis as identifiable objects, e.g., using unique (persistent) identifiers such as DOIs and/or ORCIDs. The RO approach is well aligned with the idea of interoperable and platform-independent solutions for provenance capture of workflows because of its domain-neutral and platform-independent nature.

While ROs can be serialized in several different ways, in this work we have reused the BDBag approach based on BagIt (see box), which has been shown to support large-scale workflow data [91]. This approach is also compatible with data-archiving efforts from the NIH Data Commons, Library of Congress, and the Research Data Alliance. The specialized workflow-centric RO in this study encompasses the components mentioned in the previous paragraph annotated with various targeted tools and a PROV-based *workflow provenance profile* to capture the detailed retrospective provenance of the CWL workflow enactment.

#### PROV Data Model

**Figure 4:**
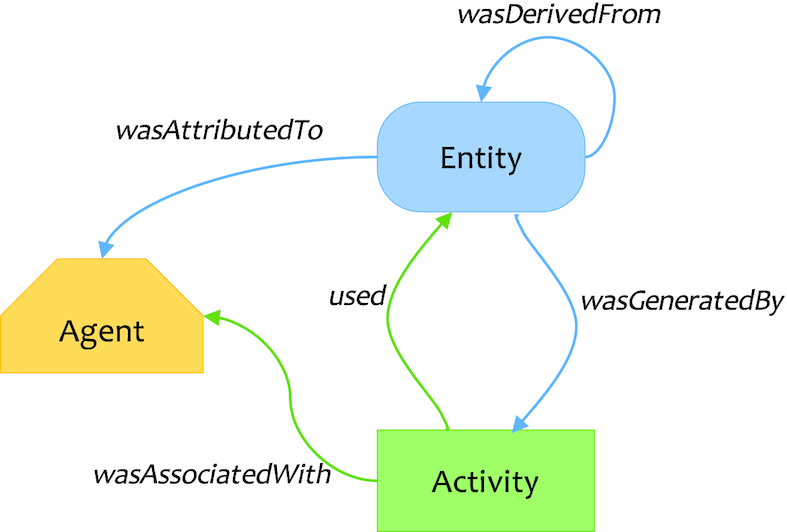
Core concepts of the PROV Data Model. Adapted from W3C PROV Model Primer [92].

The W3C developed *PROV*, a suite of specifications for unified/interoperable representation and publication of provenance information on the Web. The underlying conceptual PROV-DM [15] provides a domain-agnostic model designed to capture fundamental features of provenance with support for extensions to integrate domain-specific information (Fig. 4).

We use mainly 2 serializations of PROV for this study, PROV-Notation (PROV-N) [93] and PROV-JSON [94]. PROV-N is designed to achieve serialization of PROV-DM instances by formally representing the information using a simplified textual syntax to improve human readability. PROV-JSON is a lightweight interoperable representation of PROV assertions using JavaScript constructs and data types. The key design and implementation principles of these 2 serializations of PROV are in compliance with the goals of this study, i.e., understandable and interoperable, and hence are a natural choice to support the design of an adaptable provenance profile. For completeness we also explored serializing the provenance graph as PROV-XML [95], as well as PROV-O [96], which provides a mapping to Linked Data and ontologies, with potential for rich queries and further integration using a triple store. One challenge here is the wide variety of OWL and RDF formats; we opted for Turtle, N-Triples, and JavaScript Object Notation for Linked Data (JSON-LD) but concluded that requiring all of these PROV and RDF serializations would be an unnecessary burden for other implementations of CWLProv.

### CWLProv research object

**Figure 5:**
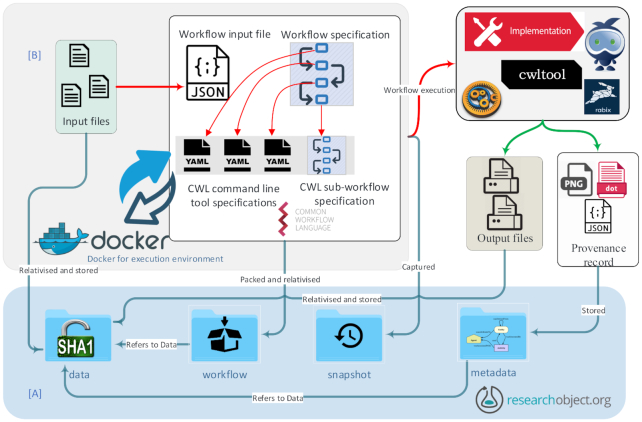
Schematic representation of the aggregation and links between the components of a given workflow enactment. Layers of execution are separated for clarity. The workflow specification and command line tool specifications are described using CWL. Each individual command line tool specification can optionally interact with Docker to satisfy software dependencies. [A] The RO layer shows the structure of the RO including its content and interactions with different components in the RO and [B] the CWL layer.

The provenance framework defined in the previous section can be satisfied by using a structured approach to share the identified resources. In this section, we define the representation of data and metadata to be shared for a given workflow enactment, stored as multiple files in their native formats. The folder structure of the CWLProv RO complies with the*BagIt* [9] format such that its content and completeness can be verified with any BagIt tool or library (see box What is BagIt?). The files used and generated by the workflow are here considered the *data payload*; the remaining directories include *metadata* of how the workflow results were created. We systematized the aggregated resources into various collections for better understanding and accessibility for a CWL workflow execution (Fig. 5).

#### data/

data/ is the *payload* collection of the RO; in CWLProv this contains all input and output files used in a given workflow enactment. Each data file should be labelled and identified based on a hashed checksum rather than derived from its file path during workflow execution. This use of *content-addressable* reference and storage [97] simplifies identifier generation for data and helps to avoid local dependencies, e.g., hard-coded file names. However, the workflow execution engine might use other unique identifiers for file objects. It is advised to reuse such identifiers to avoid redundancy and to comply with the system/platform used to run the workflow.

#### workflow/

CWLProv ROs must include a system-independent executable version of the workflow under the workflow/ folder. When using CWL, this sub-folder must contain the complete executable *workflow specification* file, an *input file object* with parameter settings used to enact the workflow and an *output file object* generated as a result of workflow enactment. The latter contains details of the workflow outputs such as data files produced by the workflow but may exclude intermediate outputs.

To ensure RO portability, these file objects may not exactly match the file names at enactment time, as the absolute paths of the inputs are recommended to be replaced with relativized *content-addressed* paths within the RO; e.g., /home/alice/exp15/sequence.fa is replaced with ../data/b1/b1946ac92492d2347c6235b4d2611184. The input file object should also capture any dependencies of the input data files, such as .bam.bai indexes neighbouring .bam files. Any folder objects should be expanded to list contained files and their file names at time of enactment.

In the case of a CWL workflow, *cwltool* can aggregate the CWL description and any referenced external descriptions (such as sub-workflows or command line tool descriptions) into a single workflow file using cwltool -pack. This feature is used in our implementation (details in section Practical Realization**of** CWLProv) to rewrite the workflow files, making them re-executable without depending on workflow or command line descriptions on the file system outside the RO. Other workflow definition approaches, WMS, or CWL executors should apply similar features to ensure that workflow definitions are executable outside their original file system location.

**What is BagIt?**

BagIt is an Internet Engineering Task Force Internet Standard (RFC8493) [9] that defines a structured file hierarchy for the purpose of digital preservation of data files. BagIt was initiated by the US Library of Congress and the California Digital Library and is now used by libraries and archives to ensure safe transmission and storage of datasets using “bags."

A bag is indicated by the presence of bagit.txt and a *payload* of digital content stored as files and sub-folders in the data/ folder. Other files are considered *tag files* to further describe the payload. All the payload files are listed in a *manifest* with checksums of their byte content, e.g.,manifest-sha256.txt and equivalent for tag files in tagmanifest-sha256.txt. Basic metadata can be provided in bag-info.txt as key-value pairs.

A bag can be verified to be *complete* if all the files listed in the manifests exist and is also considered *valid* if the manifest matches the checksum of each file, ensuring that they have been correctly transferred.

BDBag (Big Data bag) [91] is a profile of BagIt that adds an *RO* [98] metadata/manifest.json in JSON-LD [99] format to contain richer Linked Data annotations that may not fit well in bag-info.txt, e.g., authors of an individual file. BDBags can include a fetch.txt to reference external resources using *ARK MinIDs* or HTTP URLs, allowing bags that contain large files without necessarily transferring their bytes.

#### snapshot/

snapshot/ comprises copies of the workflow and tool specifications files “as-is” at enactment time, without any rewrites, packing, or relativizing as described above.

It is recommended to use snapshot resources only for validity-checking results and for understanding the workflow enactment because these files might contain absolute paths or be host-specific and thus may not be possible to re-enact elsewhere. Preserving these files untouched may nevertheless retain information that could otherwise get lost, e.g., commented-out workflow code, or identifiers baked into file names.

A challenge in capturing snapshot files is that they typically live within a file system hierarchy, which can difficult to replicate accurately, and may have internal references to other files. In our implementation we utilize cwltool -print-depsto find indirectly referenced files and store their snapshots in a flat folder.

#### metadata/

Each CWLProv RO must contain an RO manifest file metadata/manifest.json and 2 sub-directories metadata/logs and metadata/provenance. The RO manifest, part of the BDBag [91] profile, follows the JSON-LD structure defined for Research Object Bundles [98] and can provide structured Linked Data for each file in the RO, such as file type and creation date. Further detail about the manifest file contents is documented on GitHub as CWLProv specification [83].

Any raw log information from the workflow enactment should be made available in metadata/logs. This typically includes the actual commands executed for each step. Similar to the snapshot files, log files may however be difficult to process outside the original enactment system. An example of such processing is *CWL-metrics* [100], which post-processes cwltool log files to capture runtime metrics of individual Docker containers.

Capturing the details of a workflow execution requires rich metadata in provenance files (see section **Retrospective Provenance Profile**). These should exist in the sub-folder metadata/provenance. It is recommended to make the availability of a *primary* provenance file mandatory, which should conform with the PROV-N [93] format. This file describes the top-level workflow execution. As described in *Level 2* (Section **Levels of Provenance and Resource Sharing**), it is quite possible to have nested workflows. In that case, a separate provenance file for each nested workflow execution should be included in this folder. If there are additional formats of provenance files such as PROV-JSON [94], PROV-XML [95], PROV-O [96], etc., then these should be included in the said folder, with a declaration using conformsTo to declare their formats in the RO manifest being mandatory. The nested workflow profile should be named such that there is a link between the respective step in the primary workflow and the nested workflow, preferably using unique identifiers.

Because the PROV-DM has a generalized structure, there might be some provenance aspects specific to particular workflows that are hard to capture by only using PROV-N; hence, ontologies such as *wfdesc* [101] can be used to describe the abstract representation of the workflow and its steps. Use of *wfprov* [102] to capture some workflow provenance aspects is also encouraged. Alternative extensions such as ProvOne [103] can also be utilized if the WMS or workflow executor is using these extensions already.

CWLProv reuses Linked Data standards like JSON-LD [99], W3C PROV [15], and Research Object [11]. A challenge with Linked Data in distributed and desktop computing is how to make identifiers that are absolute Uniform Resource Identifiers (URIs) and hence globally unique. For example, for CWLProv a workflow may be executed by an engine that does not know where its workflow provenance will be stored, published, or finally integrated. To this end CWLProv generators should use the proposed *arcp* [104] URI scheme to map local file paths within the RO BagIt folder structure to absolute URIs for use within the RO manifest and associated PROV traces. Consumers of CWLProv ROs that do not contain an arcp-based External-Identifier should generate a temporary arcp base to safely resolve any relative URI references not present in the CWLProv folder. Implementations processing a CWLProv RO may convert arcp URIs to local file:/// or http:// URIs depending on how and where the CWLProv RO was saved, e.g., using the “arcp.py” library [105].

### Retrospective provenance profile

As stated earlier, the primary provenance file should conform to the PROV-N [93] serialization of the PROV data model and may optionally use ontologies specific to the workflow execution. The key features used in the structure of the retrospective provenance profile for a CWL workflow enactment in CWLProv are listed in Table 2. These features are not tied to any platform or workflow definition approach and hence can be used to document retrospective provenance of any workflow irrespective of the workflow definition approach.

**Table 2.** Fulfilling recommendations with the CWLProv profile of W3C PROV, extended with RO Model’s *wfdesc* (prospective provenance) and *wfprov* (retrospective provenance)

PROV type	Subtype	Relation	Range	Recommendation
Plan	wfdesc:Workflow	wfdesc:hasSubProcess	wfdesc:Process	R9-workflow
	wfdesc:Process			
Activity	wfprov:WorkflowRun	wasAssociatedWith	wfprov:WorkflowEngine	R8-environment
		↳ hadPlan	wfdesc:Workflow	R9-workflow, R17-executable
		wasStartedBy	wfprov:WorkflowEngine	R8-environment
		↳ atTime	ISO8601 timestamp	R13-provenance
		wasStartedBy	wfprov:WorkflowRun	R9-workflow
		wasEndedBy	wfprov:WorkflowEngine	R8-environment
		↳ atTime	ISO8601 timestamp	R13-provenance
	wfprov:ProcessRun	wasStartedBy	wfprov:WorkflowRun	R10-software
		↳ atTime	ISO8601 timestamp	R14-provenance
		used	wfprov:Artifact	R11-raw-data
		↳ role	wfdesc:InputParameter	R1-parameters
		wasAssociatedWith	wfprov:WorkflowRun	R9-workflow
		↳ hadPlan	wfdesc:Process	R17-executable, R16-format
		wasEndedBy	wfprov:WorkflowRun	R13-provenance
		↳ atTime	ISO8601 timestamp	R13-provenance
	SoftwareAgent	wasAssociatedWith	wfprov:ProcessRun	R8-environment
		↳ cwlprov:image	docker image id	R4-sw-version
SoftwareAgent	wfprov:WorkFlowEngine	wasStartedBy	Person ORCID	R12-attribution
		label	cwltool -version	R4-sw-version
Entity	wfprov:Artefact	wasGeneratedBy	wfprov:Processrun	R3-intermediate, R7-identifier
		↳ role	wfdesc:OutputParameter	R1-parameters
Collection	wfprov:Artefact	hadMember	wfprov:Artefact	R3-intermediate
	Dictionary	hadDictionaryMember	wfprov:Artefact	
		↳ pairKey	filename	R7-identifier

Indentation with ↳ indicates n-ary relationships, which are expressed differently depending on PROV syntax. Namespaces: http://www.w3.org/ns/prov# (default), http://purl.org/wf4ever/wfdesc# (wfdesc), http://purl.org/wf4ever/wfprov# (wfprov), https://w3id.org/cwl/prov# (cwlprov).

The core mapping is following the PROV data model as in Fig. 4: the PROV *Activity* represents the duration of a workflow run, as well as individual step executions, which *used* file and data (*Entity*), which again may be *wasGeneratedBy* previous step activities. The workflow engine (e.g., cwltool) is the *Agent* controlling these activities according to the workflow definition (*Plan*).

PROV is a general standard not specific to workflows, and it lacks features to relate a *plan* (i.e., a workflow description) with sub-plans and workflow-centric retrospective provenance elements, e.g., specific workflow enactment and its related steps enactment. We have used *wfdesc* and *wfprov* to represent a few elements of prospective and retrospective provenance, respectively. In addition, the provenance profile documented details of all the uniquely identified *activities*, e.g., workflow enactment and related command line tool invocations, and their associated *entities* (e.g., input and output data artefacts, input configuration files, workflows, and command line tool specifications). The profile also documents the relationship between activities such as which activity (workflow enactment) was responsible for starting and ending another activity (command line tool invocation).

As described in Section **Levels of Provenance and Resource Sharing**, to achieve maximum *white-box* provenance, the inner workings of a nested workflow should also be included in the provenance trace. If a step represents a nested workflow, a separate provenance profile is included in the RO. Moreover, in the parent workflow trace, this relationship is recorded using “*has_provenance*" as an attribute of the *Activity* step, which refers to the profile of the nested workflow.

## Practical Realization of CWLProv

CWLProv [83] provides a format that can be adopted by any workflow executor or platform, provided that the underlying workflow definition approach is at least as declarative as CWL; i.e., it captures the necessary components described in Section **Applied Standards and Vocabularies**. In the case of CWL, as long as the conceptual constructs are common amongst the available implementations and executors, a workflow enactment can be represented in CWLProv format. To demonstrate the practical realization of the proposed model we consider a Python-based reference implementation of *CWL cwltool*.

*cwltool* is a feature-complete reference implementation of CWL. It provides extensive validation of CWL files, as well as offering a comprehensive set of test cases to validate new modules introduced as extensions to the existing implementation. Thus it provides the ideal choice for implementing CWLProv for provenance support and resource aggregation. The existing classes and methods of the implementation were utilized to achieve various tasks such as packaging of the workflow and all associated tool specifications together. In addition, the existing Python library *prov* [106] was used to create a provenance document instance and populate it with the required artefacts generated as the workflow enactment proceeds.

It should be noted that we elected to implement CWLProv in the reference implementation *cwltool* instead of the more scalable and production-friendly CWL implementations like Toil [107], Arvados [108], Rabix [86], CWL-Airflow [109], or Cromwell [87]. An updated list of implementations is available at the CWL [110]. Compared to *cwltool* these generally have extensive scheduler and cloud compute support, and extensions for large data transfer and storage, and should therefore be considered for any adopters of the CWL. In this study we have however focused on *cwltool* because its code base was found to be easy to adapt for rich provenance capture without having to modify subsystems for distributed execution or data management, and as a reference implementation better informing us on how to model CWLProv for the general case rather than being tied into execution details of the more sophisticated CWL workflow engines.

**Figure 6:**
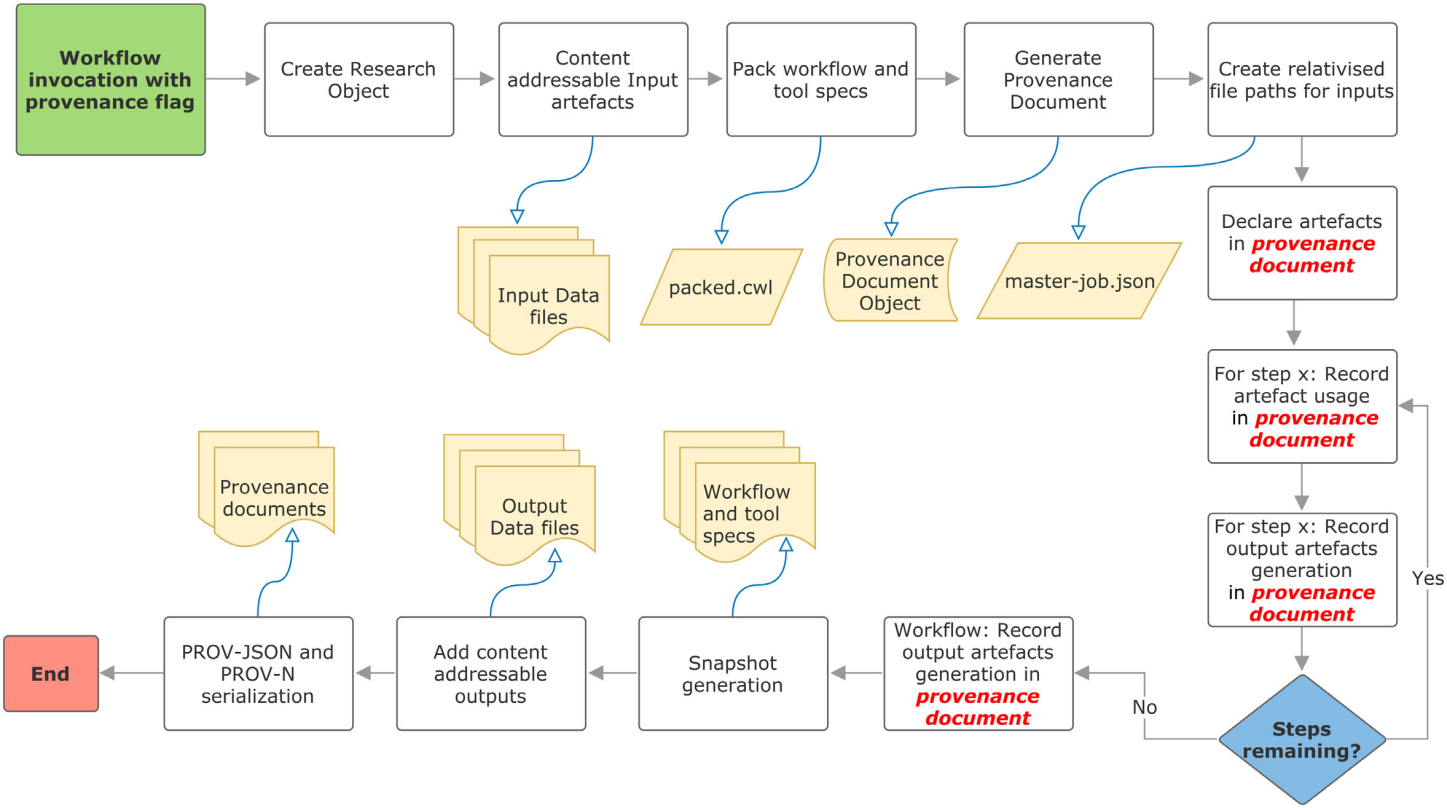
High-level process flow representation of retrospective provenance capture.

CWLProv support for *cwltool* is built as an optional module, which, when invoked as *“cwltool*-*provenance ro/ workflow.cwl job.json,"* will automatically generate an RO with the given folder name *“ro/"* without requiring any additional information from the user. Each input file is assigned a hash value and placed in the folder *“ro/data,"* making it content-addressable to avoid local dependencies (Fig. 6).

To avoid including information about attribution without consent of the user, we introduce an additional flag *“ -enable-user-provenance."*If a user provides the options -*orcid* and -*full-name*, this information will be included in the provenance profile related to user attribution. Enabling *“ -enable-user-provenance”* and not providing the full name or ORCID will store user account details from the local machine for attribution, i.e., the details of the *agent* that enacted the workflow.

The workflow and command line tool specifications are aggregated in 1 file to create an executable workflow and placed in folder *“ro/workflow."* This folder also contains transformed input job objects containing the input parameters with references to artefacts in the *ro/data* based on relativizing the paths present in the input object. These 2 files are sufficient to re-enact the workflow, provided the other required artefacts are also included in the RO and comply with the CWLProv format. The *cwltool* control flow [111] indicates the points when the execution of the workflow and command line tools involved in the workflow enactment start, end, and how the output is reported back. This information and the artefacts are captured and stored in the RO.

When the execution of a workflow begins, CWLProv extensions to *cwltool* generate a provenance document (using the *prov* library), which includes default namespaces for the workflow enactment *“activity."* The attribution details as an *agent* are also added at this stage if user provenance capture is enabled, e.g., to answer “who ran the workflow?" Each step of the workflow can correspond to either a command line tool or another nested workflow referred to as a *sub-workflow* in the CWL documentation. For each nested workflow, a separate provenance profile is initialized recursively to achieve a *white-box* finer-grained provenance view as explained in Section **Levels of Provenance and Resource Sharing**. This profile is continually updated throughout the nested workflow enactment. Each step is identified by a unique identifier and recorded as an *activity* in the parent workflow provenance profile, i.e., the *“primary profile."* The nested workflow is recorded as a step in the *primary profile* using the same identifier as the “nested workflow enactment activity” identifier in the respective provenance profile. For each step in the activity, the start time and association with the workflow activity are created and stored as part of the overall provenance to answer the question “when did it happen?"

The data used as input by these steps are either provided by the user or produced as an intermediate result from the previous steps. In both cases, the *Usage* is recorded in the respective provenance profile using checksums as identifiers to answer the question “what was used?" The non-file input parameters such as strings and integers are stored “as-is” using an additional optional argument, *prov:value*. Upon completion, each step typically generates some data. The provenance profile records the generation of outputs at the step level to record “what was produced?” and “which process produced it?" Once all steps complete, the workflow outputs are collected and the generation of these outputs at the workflow level is recorded in the provenance profile. Moreover, by using the checksum of these files generated by the *cwltool*, content-addressable copies are saved in the folder *ro/data*. The provenance profile refers to these files using the same checksum such that they are traceable or can be used for further analysis if required. The workflow specification, command line tool specifications, and JSON job file are archived in the *ro/snapshot* folder to preserve the actual workflow history.

This prototype implementation provides a model and guidance for workflow platforms and executors to identify their respective features that can be utilized in devising their own implementation of CWLProv.

### Achieving recommendations with provenance levels

Table 3 maps the best practices and recommendations from Table 1 to the Levels of Provenance (Fig. 2). The shown methods and implementation readiness indicate to which extent the recommendations are addressed by the implementation of CWLProv (detailed in this section).

**Table 3.** Recommendations and provenance levels implemented in CWLProv

Recommendation	Level 0	Level 1	Level 2	Level 3	Methods
R1-parameters	•		•		CWL, BP
R2-automate	•				CWL, Docker
R3-intermediate		•			PROV, RO
R4-sw-version	•		•		CWL, Docker, PROV
R5-data-version	•			•	CWL, BP
R6-annotation		•		*	CWL, RO, BP
R7-described		•			CWL, RO
R7-identifier		•	•	•	RO, CWLProv
R8-environment		*	*		GFD.204
R9-workflow	•	•	•		CWL, wfdesc
R10-software	•		•		CWL, Docker
R11-raw-data	•	•			CWLProv, BP
R12-attribution		•			RO, CWL, BP
R13-provenance		•	•		PROV, RO
R14-diagram	▓			*	CWL, RO
R15-open-source	•				CWL, BP
R16-format		•		•	CWL, BP
R17-executable	▓	•			CWL, Docker
R18-resource-use		*	*		CWL, GFD.204
R19-example	*	▓			RO, BP

BP: best practices need to be followed manually; CWL: Common Workflow Language and embedded annotations; CWLProv: additional attributes in PROV; PROV: W3C Provenance model; RO: RO model and BagIt; wfdesc: prospective provenance in PROV.

• Implemented.

▓ Partially implemented.

*Implementation planned/ongoing.

Note that other approaches may solve this mapping differently. For instance, Nextflow [112] may fulfill *R18-resource-use* at Provenance Level 2 because it can produce trace reports with hardware resource usage per task execution [113], but not for the overall workflow. While a Nextflow trace report is a separate CSV file with implementation-specific columns, our planned *R18-resource-use* approach for CWL is to combine *CWL-metrics* [114], permalinks, and the standard *GFD.204* [115] to further relate resource use with Level 1 and Level 2 provenance within the CWLProv RO.

In addition to following the recommendations from Table 1 through computational methods, the workflow authors are also required to exercise *best practices for workflow design and authoring*. For instance, to achieve *R1-parameters* the workflow must be written in such a way that parameters are exposed and documented at the workflow level, rather than hard-coded within an underlying Python script. Similarly, while the CWL format supports rich details of user annotations that can fulfill *R6-annotation*, for these to survive into an RO at execution time, such annotation capabilities must actually be used by workflow authors instead of unstructured text files.

It should be a goal of a scientific WMS to guide users towards achieving the required level of the provenance framework through automation where possible. For instance, a user may in the workflow have specified a Docker container image without preserving the version, but the provenance log could still record the specific container version used at execution time, achieving *R4-sw-version* retrospectively by computation rather than relying on a prospective declaration in the workflow definition.

## CWLProv Evaluation with Bioinformatics Workflows

CWLProv as a standard supports *syntactic, semantic*, and *pragmatic* interoperability (defined in Section **Interoperability**) of a given workflow and its associated results. We have defined a *“common data format”* for workflow sharing and publication such that any executor or WMS with CWL support can interpret this information and make use of it. This ensures the *syntactic* interoperability between the workflow executors on different computing platforms. Similarly the *“content”* of the shared aggregation artefact as a workflow-centric RO is unambiguously defined, thus ensuring uniform representation of the workflow and its associated results across different platforms and executors, hence supporting *semantic* interoperability. With Level 3 provenance satisfied providing domain-specific information along with level 0–2 provenance tracking, we posit that CWLProv would be able to accomplish *pragmatic* interoperability by providing unambiguous information about the *“context," “application,”* and *“use”* of the shared/published workflow-centric ROs. Hence, extension of the current implementation (described in section **Practical Realization of CWLProv**) in future to include domain-rich information in the provenance traces and the CWLProv RO will result in pragmatic interoperability.

To demonstrate the interoperability and portability of the proposed solution, we evaluate CWLProv and its reference implementation using open source bioinformatics workflows available on GitHub from different research initiatives and from different developers. Conceptually, these workflows are selected for evaluation owing to their extensive use in real-life data analyses and variety of the input data. Alignment workflow is included in the evaluation because it is one of the most time-consuming yet mandatory steps in any variant calling workflow. Practically, choosing the workflows by these particular groups out of numerous existing implementations is justified in each section below.

### RNA-seq analysis workflow

RNA sequencing (RNA-seq) data generated by next-generation sequencing platforms is composed of short sequence reads that can be aligned to a reference genome, where the alignment results form the basis of various analyses such as quantitating transcript expression and identifying novel splice junctions and isoforms and differential gene expression [116]. RNA-seq experiments can link phenotype to gene expression and are widely applied in multi-centric cancer studies [20]. Computational analysis of RNA-seq data is performed by different techniques depending on the research goals and the organism under study [117]. The workflow [118] included in this case study has been defined in CWL by one of the teams [119] participating in the NIH Data Commons initiative [120], a large research infrastructure program aiming to make digital objects (such as data generated during biomedical research and software/tools required to utilize such data) shareable and accessible and hence aligned with the FAIR principles [56].

**Figure 7:**
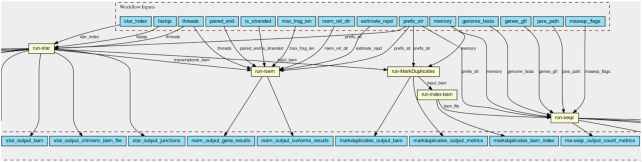
Portion of an RNA-seq workflow generated by CWL viewer [129].

This workflow (Fig. 7), designed for the pilot phase of the NIH Data Commons initiative [121], adapts the approach and parameter settings of Trans-Omics for Precision Medicine (TOPMed) [122]. The RNA-seq pipeline originated from the Broad Institute [123]. There are 5 steps in the workflow: (i) Read alignment using STAR [124] produces aligned BAM files including the genome BAM and transcriptome BAM. (ii) The genome BAM file is processed using Picard MarkDuplicates [125], producing an updated BAM file containing information on duplicate reads (such reads can indicate biased interpretation). (iii) SAMtools index [126] is then used to generate an index for the BAM file, in preparation for the next step. (iv) The indexed BAM file is processed further with RNA-SeQC [127], which takes the BAM file, human genome reference sequence, and Gene Transfer Format (GTF) file as inputs to generate transcriptome-level expression quantifications and standard quality control metrics. (v) In parallel with transcript quantification, isoform expression levels are quantified by RSEM [128]. This step depends only on the output of the STAR tool, and additional RSEM reference sequences.

For testing and analysis, the workflow author provided example data created by down-sampling the read files of a TOPMed public access data set [130]. Chromosome 12 was extracted from the *Homo Sapien Assembly 38* reference sequence and provided by the workflow authors. The required GTF and RSEM reference data files are also provided. The workflow is well documented, with a detailed set of instructions for the steps performed to down-sample the data also provided for transparency. The availability of example input data, use of containerization for underlying software, and detailed documentation are important factors in choosing this specific CWL workflow for CWLProv evaluation.

### Alignment workflow

**Figure 8:**
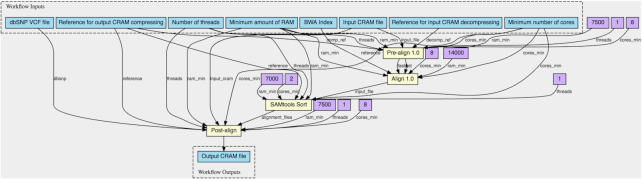
Alignment workflow representation generated by CWL viewer.

Alignment is an essential step in variant discovery workflows and considered an obligatory *preprocessing* stage according to best practices by the Broad Institute [71]. The purpose of this stage is to filter low-quality reads before variant calling or other interpretative steps [131]. The workflow for alignment is designed to operate on raw sequence data to produce analysis-ready BAM files as the final output. The typical steps followed include file format conversions, aligning the read files to the reference genome sequence, and sorting the resulting files. The CWL alignment workflow [132] included in this evaluation (Fig. 8) is designed by Data Biosphere [133]. It adapts the alignment pipeline [134] originally developed at Abecasis Lab, University of Michigan [135]. This workflow is also part of the NIH Data Commons initiative (as RNA-seq Analysis Workflow) and comprises 4 stages. First, “Pre-align” accepts a CRAM file (a compressed format for BAM files developed by EBI [136]) and human genome reference sequence as input and, using underlying software utilities of SAMtools such as view, sort, and fixmate, returns a list of fastq files, which can be used as input for the next step. The next step “Align” also accepts the human reference genome as input along with the output files from “Pre-align” and uses BWA-mem [137] to generate aligned reads as BAM files. SAMBLASTER [138] is used to mark duplicate reads and SAMtools view to convert read files from SAM to BAM format. The BAM files generated after “Align” are sorted with “SAMtool sort." Finally these sorted alignment files are merged to produce a single sorted BAM file using SAMtools merge in the “Post-align” step. The authors provide an example CRAM file, *Homo Sapien Assembly 38* reference genome, along with its index files, to be used as inputs for testing and analysis of the workflow.

### Somatic variant calling workflow

Variant discovery analysis for high-throughput sequencing data is a widely used bioinformatics technique, focused on finding genetic associations with diseases, identifying somatic mutations in cancer, and characterizing heterogeneous cell populations [139]. The *preprocessing* explained for the Alignment workflow is part of any variant calling workflow as reads are classified and ordered as part of the variant discovery process. Numerous variant calling algorithms have been developed depending on the input data characteristics and the specific application area [131]. Somatic variant calling workflows are designed to identify somatic (non-inherited) variants in a sample—generally a cancer sample—by comparing the set of variants present in a sequenced tumour genome to a non-tumour genome from the same host [140]. The set of tumour variants is a super-set of the set of host variants, and somatic mutations can be identified through various algorithmic approaches to subtracting host familial variants. Each somatic variant calling workflow typically consists of 3 stages: preprocessing, variant evaluation, and post-filtering.

**Figure 9:**
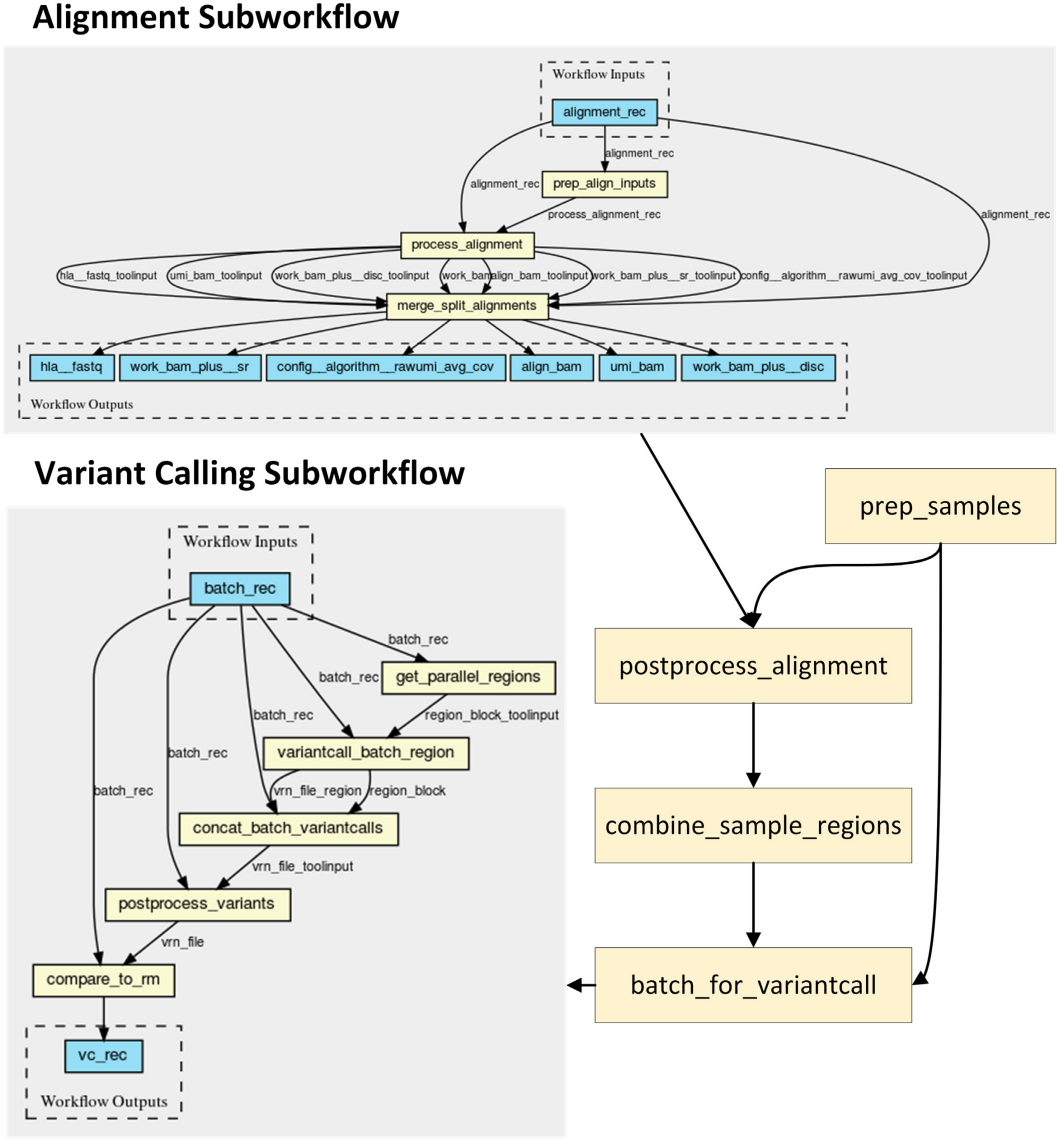
Visual representation of the bcbio somatic variant calling workflow (adapted from [143]). The subworkflow images are generated by CWL viewer .

The somatic variant calling workflow (Fig. 9) included in this case study was designed by Blue Collar Bioinformatics (bcbio) [141], a community-driven initiative to develop best-practice pipelines for variant calling, RNA-seq, and small RNA analysis workflows. According to the documentation, the goal of this project is to facilitate the automated analysis of high-throughput data by making the resources *quantifiable, analyzable, scalable, accessible*, and *reproducible*. All the underlying tools are containerized, facilitating software use in the workflow. The somatic variant calling workflow defined in CWL is available on GitHub [142] and equipped with a well-defined test dataset.

### Evaluation activity

This section describes the evaluation of cross-executor and cross-platform interoperability of CWLProv. To test cross-executor interoperability, 2 CWL executors, *cwltool* and *toil-cwl-runner*, were selected. *toil-cwl-runner* is an open source Python workflow engine supporting robust cross-platform workflow execution on cloud and high-performance computing environments [107]. The 2 operating system platforms utilized in this analysis were MacOS and Ubuntu Linux. For the Linux OS, a 16-core Linux instance with 64 GB RAM was launched on the Australian National eResearch Collaboration Tools and Resources (NeCTAR) research cloud [144]. To cater to the storage requirements, a 1000-GB persistent volume was attached to this instance. For MacOS, a local system with 16 GB RAM, 250 GB storage, and 2.8-GHz Intel Core i7 processor was used. These platforms were selected to cater to the required storage and compute resources of the workflows described above. The reference genome provided with Alignment Workflow was not down-sampled, and hence this workflow required the most resources among the 3 evaluated.

This evaluation does not include details of the installation process for *cwltool, toil-cwl-runner*, and *Docker* on the systems described above. To create CWLProv ROs during workflow execution, it is necessary to use the CWL reference runner (*cwltool*) until this practice spreads to other CWL implementations. Moreover, it is assumed that the software container (Docker) should also be installed on the system to use the workflow definitions aggregated in a given CWLProv RO.

In addition, the resource requirements (identified in *R18-resource-use* and discussed in Section **Discussion and Future Directions**) should also be satisfied by choosing a system with enough compute and storage resources for successful enactment. The systems used in this case study should be a reference when selecting a system because inadequate compute and storage resources such as insufficient RAM or number of cores will hinder the successful re-enactment of workflows using these ROs. The hardware requirements may also vary if a different dataset is used as input to re-enact the workflow using the methods aggregated in the RO. In that case, the end user must ensure the availability of adequate compute and storage resources by choosing a system that meets specifications [145].

Because the CWLProv implementation is demonstrated for one of the executors (*cwltool*), currently a CWLProv RO for any workflow can only be produced using *cwltool*. Hence, in this activity the workflows are initially enacted using just *cwltool* (Table 4). The outline of the steps performed to analyse CWLProv for each case study is as follows.

The workflow was enacted using *cwltool* to produce an RO on a MacOS computer.
  The resulting RO and aggregated resources were used to re-enact the workflow using *toil-cwl-runner* on the same MacOS computer;
  The RO produced in step I was transferred to the cloud-based Linux instance used in this activity;
  On the cloud-based Linux environment and only utilizing the resources aggregated in the RO, the workflow was re-enacted using *cwltool* and *toil-cwl-runner*.
The workflow was enacted using *cwltool* to produce an RO on Linux.
  The resulting RO and aggregated resource were utilized to re-enact the workflow using *toil-cwl-runner* on the same cloud-based Linux instance;
  The RO produced in step II was transferred to the MacOS computer used in this activity;
  On the MacOS computer and only utilizing the resources aggregated in the RO, the workflow was re-enacted using *cwltool* and *toil-cwl-runner*.

**Table 4.** CWLProv evaluation summary and status for the 3 bioinformatics case studies

Enact-produce RO with	Re-enact using RO with	Status
cwltool on MacOS	toil-cwl-runner on MacOS	✓
	cwltool on Linux	✓
	toil-cwl-runner on Linux	✓
cwltool on Linux	toil-cwl-runner on Linux	✓
	cwltool on MacOS	✓
	toil-cwl-runner on MacOS	✓

The CWLProv ROs produced as a result of this activity are published on Mendeley Data [146–148] with mirrors on Zenodo.

### Evaluation results

The steps described above were taken to produce ROs, which were then used to re-enact the workflows (outlined in Table 4), without any further changes required. This demonstration illustrated the syntactic and semantic interoperability of the workflows across different systems. It shows that **both CWL executors were able to *exchange, comprehend*, and *use* the information represented as CWLProv ROs**. The current implementation described in section **Practical Realization of CWLProv** does not resolve *Level 3*. Hence, the inclusion of domain-specific annotations referring to scientific context to address pragmatic interoperability is identified as a crucial future direction and further detailed in section **Discussion and Future Directions**.

#### *CWLProv* and interoperability

CWL already builds on technologies such as JSON-LD [99] for data modeling and Docker [26] to support portability of the runtime environments. The portability and interoperability as basic principles of the underlying workflow definition approach for any workflow-centric analysis implies that the analysis should also be portable and interoperable. However, the workflow definition/specification alone is insufficient when dealing with command line tool specifications, data, and input configuration files used in the analysis if these are not readily available.

CWLProv ensures availability of these resources for a given analysis conforming to the framework defined in Section **CWLProv 0.6.0 and utilized standards**. The input configurations are saved as *primary-job.json* in folder *workflow*/ and refer to the input data contained in the payload *data*/ folder of the given RO. In this way, availability of data aggregated with the analysis is made possible. Existing features of *cwltool* are used to generate the CWL workflow specification file containing all of the command line tool specifications referred to in the workflow specification and placed in the same *workflow*/ folder.

One might argue that copying a folder tree might serve the same purpose, but in that case we again will be relying on users to exert a substantial amount of effort beyond the actual analysis; i.e., they would have to carefully structure their directories to be aligned with the workflow creators. Instead CWL encourages researchers to use container technologies such as Docker, Singularity, or software packaging systems like Debian (Med) or Bioconda to ensure availability of underlying tools as recommended by numerous studies [8,55,63, 64,149]. This practice facilitates the preservation of methods utilized in data-intensive scientific workflows and enables verification of the published claims without requiring the end user to do any manual installation and configuration. Examples of tools available via Docker containers used here are the alignment tool (BWA-mem) used in the Alignment workflow and STAR aligner used in the RNA-seq workflow.

#### Evaluating provenance profile

The retrospective provenance profile generated as part of CWLProv for each workflow enactment can be examined and queried to extract the required subset of information. *Provenance analytics* is a separate domain and a next step after provenance collection in the provenance life cycle [150]. Often provenance data are queried using specialized query languages such as SQL SPARQL or TriQL depending on the storage mechanism used. Query operations can combine information from prospective and retrospective provenance to understand computational experiments better.

The focus of this paper is not in-depth provenance analytics, but we have demonstrated the application of the provenance profile generated as part of CWLProv. We have developed a command line tool and Python API *“cwlprov-py”* [151] for CWLProv RO analytics to interpret the captured retrospective provenance of CWL workflow enactment. This API currently supports the following use cases.

Given a CWLProv RO:

- **Workflow Runs** As each RO can contain >1 *workflow run* if sub-workflows are utilized to group related tasks into 1 workflow. In that case, the provenance traces are stored in separate files for each workflow run. cwlprov-py identifies the workflow enactments including the sub-workflows (*if any*) and returns the workflow identifiers annotated with the step names. The user can select the required trace and explore particular traces in detail.
- **Attribution** Each RO is assumed to be associated with a single enactment of the primary workflow and hence assumed to be enacted by 1 person. As discussed previously, CWLProv provides additional flags to enable user provenance capture. A user can provide their name and ORCID details that can be stored as part of an RO. cwlprov-py displays attribution details of the researcher responsible for the enactment (*if enabled*) and the versions of the workflow executor utilized in the analysis.
- **Input/Output of a Process** Provenance traces contain associations between the steps/workflows and the data they used or generated. A user interested in a particular step can identify the inputs used and outputs produced linked explicitly to that process using cwlprov-py. This option works using individual step identifiers (level 1) as well as nested workflows (level 2), facilitating reuse of intermediate data even if the original workflow author did not explicitly expose these as workflow outputs.
- **Partial Reruns** Rerunning or reusing only desired parts of a given workflow has been emphasized [20] as important to evaluate the workflow process or validate the published results associated without necessarily re-enacting the workflow as a whole. cwlprov-py uses the identifier of the step/workflow to be rerun, parses the provenance trace to identify the inputs required, and ultimately creates a JSON input object with the associated input parameters. This input object can then be used for partial reruns of the desired step/workflow, making segmented analysis possible even for CWLProv consumers who do not have sufficient hardware resources for re-executing more computationally heavy steps.

While the above explores some use cases for consuming and reusing workflow execution data, we have not explored this in full detail. Further work could develop more specific user scenarios and perform usability testing with independent domain experts who have not seen the executed workflow before.

An important point of CWLProv is to capture sufficient information at workflow execution time, so that post-processing (potentially by a third party) can support unforeseen queries without requiring instrumentation at workflow design time. For instance, cwlprov runtimes calculates average runtime per step (requiring capture of start/stop time of each step iteration), while cwlprov derived calculates derivation paths back to input data (requiring consistent identifiers during execution). Further work could build a more researcher-oriented interface based on this approach, e.g., hardcoded data exploration for a particular workflow.

#### Temporal and spatial overhead with provenance

Table 5 shows the runtimes for the 3 workflow enactments using cwltool and toil-cwl-runner on Linux and MacOS with and without enabling provenance capture as described in the evaluation activity section. These workflows were enacted at least once before this time calculation; hence, the timing does not include the time for Docker images to be downloaded. On a new system, when these workflows are being rerun for the first time, the Docker images will be downloaded and may take significantly longer than the time specified here, especially in case of the Somatic Variant Calling workflow because of the image size.

**Table 5.** Runtime comparison for the workflow enactments done cross-executor and cross-platform

Workflow	Linux			MacOS		
	cwltool		toil-cwl-runner	cwltool		toil-cwl-runner
RNA-Seq Analysis Workflow	With Prov	Without Prov	Without Prov	With Prov	Without Prov	Without Prov
	4m30.289s	4m0.139s	3m46.817s	3m33.306s	3m41.166s	3m30.406s
Alignment Workflow	28m23.792s	24m12.404s	15m3.539s	–	162m35.111s	146m27.592s
Somatic Variant Calling Workflow	21m25.868s	19m27.519s	7m10.470s	17m26.722s	17m0.227s	**

** This could not be tested because of a Docker mount issue on MacOS: https://github.com/DataBiosphere/toil/issues/2680.

– This could not be tested because of the insufficient hardware resources on the MacOS test machine; hence. step I of the evaluation activity could not be performed for this workflow.

Runtime and storage overheads are important for provenance-enabled computational experiments. The choice of different operating systems and provenance capture mechanisms such as operating-system–level, application-level, or workflow-level as well as input/output workload, interception mechanism, and fine-grained information capture are key for provenance [152,153].

In our case study, a substantial time difference can be seen for the alignment workflow that used the most voluminous dataset, hence producing a sizable RO as well. This was due to the RO-generation where data were aggregated within the RO. The difference between the provenance-enabled enactment vs the enactment without provenance is barely noticeable for the other 2 workflow enactments with the smaller datasets. The discussion about handling the big “-omics" data such as human genome reference sequence, its index files, and other database files (e.g., dbsnp) in Section **Discussion and Future Directions** provides a possible solution to avoid such overheads.

In addition, noticeable time difference between the cwltool and *toil-cwl-runner* enactments is because of the default parallel vs serial job execution in the case of toil-cwl-runner and *cwltool*, respectively. The “scatter” operation in CWL, when applied to ≥1 input parameters of a workflow step or a sub-workflow, supports parallel execution of the associated processes. Parallelism is also available without “scatter” when separate processes have all their inputs ready. If sufficient compute resources are available, these jobs will be enacted concurrently; otherwise they are queued for subsequent execution. Compute-intensive steps of a workflow can benefit from scatter features for parallel execution by reducing the overall runtime. Both alignment and somatic variant calling workflows utilize the scatter feature to enable higher degrees of parallel job execution in the case of *toil-cwl-runner*, which explains the time difference for the cross-executor of these 2 workflows. The difference is negligible for the RNA-Seq workflow, which is composed of serial jobs with comparatively small test data.

#### Output comparison across enactments

We compared the workflow outputs after each enactment to observe the concordance and/or discordance (if any) for the workflow enactment results produced across the platforms and across the executors. As CWLProv RO refers to the data with hashed checksums, these checksums are utilized for the result comparison. The comparison was made between the output files generated by the different enactments against a single *“truth-set”* output file available and checksum in the respective Git repositories.

The checksum of the output data generated cross-platform and cross-executor comparison data as a result of the initial enactments and reruns using the CWL ROs to elicit the concordance in all but 1 cases. The “correctness” as well as agreement of these outputs given different execution environments (e.g., platform and executor) held true except for the Alignment workflow. The Alignment workflow produced varying outputs after every execution even with the same executor and platform. The output of the alignment algorithm, “BWA mem,” used in this workflow was non-deterministic because it depended on the *number of threads -t* and the *seed length -K*, which affected the output produced. While the seed length in this case was set to a constant value, the number of threads varied depending on the availability of hardware resources at runtime, thereby resulting in varying output for the same input files.

## Discussion and Future Directions

This section discusses the current and future work with reference to enriched provenance capture and smart resource aggregation, and enhancements to both the CWLProv standard and implementation.

### Compute and storage resources

The CWLProv format encapsulates the data and workflow definitions involved in a given workflow enactment along with its retrospective provenance trace. CWL as a standard provides constructs to declare basic hardware resource requirements such as minimum and maximum cores, RAM, and reserved file system storage required for a particular workflow enactment. The workflow authors can provide this information in the *“requirements”* or *“hints”* section as *“ResourceRequirement."* These requirements/hints can be declared at workflow or individual step level to help platforms/executors to allocate the required resources. This information indirectly stores some aspects of prospective view of provenance with respect to hardware requirements of the underlying system used to enact a workflow. Currently this information is only available if declared as part of workflow specification. In future, we plan to include these requirements as part of provenance for a given workflow such that all such information is gathered in one space and users are not required to inspect multiple sources to extract this information. This information can then be used as a precondition for potential successful enactment of a given workflow.

Because CWLProv is focused on retrospective provenance capture of workflow enactment, we plan to include provenance information about the compute and storage resources utilized in a given enactment to fulfill *R18-resource-use*. We believe that documenting these resources will allow users to analyse their environment and resource allocations before execution, as opposed to trial-and-error methods that may result in multiple failed enactments of a given workflow. Despite the importance of this factor, most existing provenance standards surprisingly lack dedicated constructs to represent the underlying hardware resource usage information as part of prospective or retrospective provenance. In the case of complex workflows using distributed resources, where each step could be executed on a different node/server, including all this information in a single *PROV* profile will clutter the profile and render it potentially incomprehensible. Therefore, we plan to add a separate *Usage Record* document in the RO conforming to GFD.204 [115] to describe *Level 1* (and potentially *Level 2*) resource usage in a common format independent of actual execution environment.

Capturing such resource usage records requires a tighter integration with the execution platform, so we consider this future work better suited for a cloud-based CWL engine like *Toil* or *Arvados* because the reference implementation *cwltool* does not exercise fine-grained control of its task execution. Detailed raw log files can also be provided as *Level 0* provenance, as we have demonstrated with cwltool, but these will by their nature be custom per execution platform and thus should be considered unstructured. Some related work already exploring this approach is *cwl-metrics* [114], which analyses raw *cwltool* log files in combination with detailed Docker invocation statistics using the container monitoring tool *Telegraf*. Ongoing collaboration is exploring adding these metrics as additional provenance to the CWLProv RO with summaries in PROV and GFD.204 formats.

### Provenance profile augmented with domain knowledge

CWLProv benefits from existing best practices proposed by numerous studies (Table 1) and includes defined standards for workflow representation, resource aggregation, and provenance tracking (Section **Applied Standards and Vocabularies**). We posit that the principle of following well-defined data and metadata standards enables explicit data sharing and reuse. In order to include rich metadata for bioinformaticians to produce specialized ROs for bioinformatics to achieve CWLProv *Level 3* as defined in section **Levels of Provenance and Resource Sharing**, we are investigating reuse of concepts from the BioCompute Object project [41]. This domain-specific information is not necessary for computation and execution but for understandability of the shared resources. We encourage workflow authors to include such metadata and external identifiers for data and underlying tools, e.g., EDAM identifiers for the resources used in designing a given workflow. The plan is to extract these annotations and represent them in the retrospective provenance profile in CWLProv to ultimately achieve pragmatic interoperability by providing domain-specific scientific context of the experiments. Domain-specific information is essential in determining the nature of inputs, outputs, and context of the processes linked to a given workflow enactment [73]. This information can be captured in the RO if and only if the workflow author adds it in the workflow definition; thus, achieving CWLProv *Level 3* depends on the individual workflows.

### Big -omics data

While aggregating all resources as 1 downloadable object improves reproducibility, the size of the resulting RO is an important factor in practice. On one hand, completeness of the resources contributes towards minimizing the *workflow decay* phenomenon by least dependence on availability of third-party resources. On the other hand, the nature of -omics data sizes can result in hard-to-manage workflow-centric ROs also leading to the spatial and temporal overheads as discussed in evaluation.

One solution is archiving the big datasets in online repositories or data stores and including the existing persistent identifiers and checksums in the RO instead of the actual data files, as previously demonstrated with BDBags [91,154]. While CWL executors *like toil-cwl-runner* can be configured to deposit data in a shared repository, the *cwltool* reference implementation explored in this study can only write to the local file system. External references raise the risk of unavailability of data at a later time. Therefore we recommend including the data in the RO if sufficient network and storage resources are available. Future work may explore post-processing CWLProv ROs to replace large data files with references to stable data repositories, producing a slimmer RO for transfer where individual data items can be retrieved on demand, as well as reducing data duplication across multiple related ROs.

### Improving CWLProv efficiency with selective provenance capture

*Shim* refers to an adaptor step to resolve format incompatibility issues between 2 workflow tasks [60], typically converting the previous output into an acceptable format for the next step. For example in our case study *RNA-seq* workflow, *RNA-SeQC* requires an indexed BAM file, whereas the output of *STAR* or *Picard MarkDuplicates* only comprises the BAM file alone. Hence, a shim step executing *SAMtools index* makes the aligned reads analysis ready for RNA-SeQC. Compared to the more analytical steps, the provenance of such shim steps is not particularly interesting for domain scientists, and in many cases their intermediate data would effectively double the storage cost with little information gain because such data can be reliably recreated by re-applying the predictable transformation step (considering it as a *pure function* without side effects). Another type of ignorable steps could be purely diagnostic: which outputs are used primarily during workflow design to verify tool settings. A workflow engine does not necessarily know which steps are “boring” (the CWL 1.1 specification will add a hint WorkReuse for this purpose), and our proof-of-concept implementation will dutifully store provenance from all steps.

To improve efficiency, future CWLProv work could add options to ignore capturing outputs of specified *shim* steps or to not store files larger than a particular size. Similarly a scientist or a WMS may elect to only capture provenance at a particular provenance level (see Section **Levels of Provenance and Resource Sharing**). Provenance captured under such settings would be “incomplete” (e.g., PROV would say *RNA-SeQC* consumed an identified BAM index file, but the corresponding bytes would not be stored in the RO); thus, it is envisioned that this can be indicated in the RO manifest as a variant of the CWLProv profile identifier to give the end user clear indication of what to expect in terms of completeness, so that tools like cwlprov-py could be extended to recreate missing outputs, verifying their expected checksums, or collapse provenance listing of “boring” steps to improve human tractability.

### Enforcement of best practices—an open problem

Recommendations and best practices from the scientific community are proposed frequently, to guide researchers to design their computational experiments in such a way as to make their research reproducible and verifiable. Not only the best practices for workflow design, but also for resource declaration, software packaging, and configuration management are put forward [149] to avoid dependence on local installations and manual processes of dependency management. The term *“better software, better research”* [155] can also be applied to and adapted for the workflow design process.

Declarative approaches to workflow definition such as CWL facilitate and encourage users to explicitly declare everything in a workflow, improving white-box view of the retrospective as well as prospective provenance. Such workflows should provide insights of the complete process followed, to produce a data artefact resolving the black-boxness often associated with the workflow provenance. However, it is entirely up to researchers to leverage these approaches to produce well-defined workflows with explicit details facilitating enriched capture of the provenance trace at the appropriate level, and this can require considerable effort and consistency on the workflow designer’s behalf. For instance, the alignment workflow used in this case study embeds bash scripts into the CWL tool definition, therefore requiring another layer to be penetrated for provenance information extraction. Despite using CWL for the workflow definition and CWLProv for provenance capture, the provenance trace will be missing critical information, making it coarse-grained, and the raw logs capturing the enactment will also not be as informative.

The 3 criteria defined by Cohen-Boulakia et al. [20] to be followed by workflow designers are modularized specifications, unified representation, and workflow annotations. CWL facilitates a modular structure to workflow definitions by coupling similar steps to *subworkflows*; and, as an interoperable standard, CWL provides a common platform moving towards resolution of the heterogeneity of the workflowj specification languages. In addition, users can add standardized domain-specific annotations to data and workflows incorporating the constructs defined by external ontologies (e.g., EDAM) to enhance understanding of the shared specification and the resources it refers to. All these features can be used to design better workflows and maximize the information declaration, resulting in semantically rich and provenance-complete CWLProv ROs, and should thus be expressed clearly in user guides [156] for workflow authors.

The usability of any CWLProv RO directly relies on the choice of practices followed by the researchers to design and communicate their computational analyses. Workflow-centric initiatives similar to *“software carpentry"* [157] and *“code is science"* [158] are one possible way to organize training and publicize best practices. Community-driven efforts to further consolidate the understanding of requirements to make a given workflow explicit and understandable should be made. Not only awareness about the workflow design is needed, but also the availability of the associated resources should be emphasized, e.g., software as containers or software packages, big datasets in public repositories, and preprocessing/post-processing as part of workflow. Without putting proposed best practices into actual practice, complete communication and hence the reproducibility of a workflow-centric computational analysis is likely to remain challenging.

## Conclusion

The comprehensive sharing and communication of the computational experiments used to achieve a scientific objective establishes trust in published results. Shared resources are sometimes rendered ineffective by incomplete provenance, heterogeneity of platforms, unavailability of software, and limited access to data. In this context, the contributions of the present study are 4-fold. First, we have provided a comprehensive summary of the recommendations put forward by the community regarding workflow design and resource sharing. Second, we define a hierarchical provenance framework to achieve homogeneity in the granularity of the information shared, with each level addressing specific provenance recommendations.

Third, we leverage the existing standards best suited to define a standardized format, CWLProv, for methodical representation of workflow enactments, provenance, and the associated artefacts used. Finally, to demonstrate the applicability of CWLProv, we extend an existing workflow executor (*cwltool*) to provide a reference implementation to generate interoperable workflow-centric ROs, aggregating and preserving data and methods to support the coherent sharing of computational analyses and experiments.

With any published scientific research, statements such as *“methods and data are available upon request”* should no longer be acceptable in a modern open-science–driven research community. Considering on one hand the collaborative nature and emerging openness of bioinformatics research and on the other hand the heterogeneity of workflow design approaches, it is essential to provide open access to the structured representation of the data and methods used in any scientific study to achieve interoperable solutions facilitating reproducibility of science.

Provenance capture and its subsequent use to support published research transparency should not be treated as an afterthought but rather as a standard practice of utmost priority. With adoption of well-defined standards for provenance and declarative workflow definition approaches, the assumption of black-box provenance often associated with workflows can be addressed. The workflow authors should be encouraged to follow well-established and consensus best practices for workflow design and software environment deployment. In conclusion, we do not require new standards, new WMSs, or indeed new best practices; instead the focus should be to implement, utilize, and reuse existing mature community-driven initiatives to achieve consensus in representing different aspects of computational experiments.

## Availability of source code and requirements

CWLProv is implemented as part of the CWL reference implementation cwltool:

- Project name: cwltool (RRID:SCR_015528)
- Project home page: https://github.com/common-workflow-language/cwltool
- Version: 1.0.20181012180214 [13]
- Operating system(s): Platform independent
- Programming language: Python 3.5 or later (RRID:SCR_008394)
- Other requirements: Docker (RRID:SCR_016445) recommended
- License: Apache License, Version 2.0

The CWLProv profile documents the use of W3C PROV in an RO to capture a CWL workflow run:

- Project name: CWLProv profile
- Project home page: https://w3id.org/cwl/prov
- Version: 0.6.0 [83]
- Operating system(s): Platform independent
- License: Apache License, Version 2.0

The CWLProv Python Tool can be used to explore CWLProv ROs on the command line:

- Project name: CWLProv Python Tool (cwlprov-py)
- Project home page: https://github.com/common-workflow-language/cwlprov-py
- Version: 0.1.1 [151]
- Operating system(s): Platform independent
- Programming language: Python 3.5 or later (RRID:SCR_008394)
- License: Apache License, Version 2.0

## Availability of supporting data and materials

CWLProv ROs of CWL workflow executions are published in Mendeley Data and mirrored to Zenodo.

- CWL run of Somatic Variant Calling Workflow (CWLProv 0.5.0 RO) [148]
  https://zenodo.org/record/2841641
- CWL run of Alignment Workflow (CWLProv 0.6.0 RO) [147]
  https://zenodo.org/record/2632836
- CWL run of RNA-seq Analysis Workflow (CWLProv 0.5.0 RO) [146]
  https://zenodo.org/record/2838898

The CWLProv Python Tool can be used to explore the above ROs.

The data and methods supporting this work are also available in the *GigaScience repository*, GigaDB [159].

## Abbreviations

API: Application Programming Interface; AWE: Argonne Workflow Engine; BAM: Binary Alignment Map; BWA: Burrows-Wheeler Aligner; CRAM: Compressed Alignment Map; CSV: comma separated values; CWL: Common Workflow Language; DOI: Digital Object Identifier; EBI: European Bionformatics Institute; FAIR: Findable, Accessible, Interoperable, and Reusable; GATK: Genome Analysis ToolKit; GUI: graphical user interface; JSON-LD: JavaScript Object Notation for Linked Data; NIH: National Institutes of Health; OPM: Open Provenance Model; OS: Operating System; OWL: Web Ontology Language; PROV-DM: PROVenance Data Model; PROV-N: PROV-Notation; RNA-seq: RNA sequencing; RO: research object; TOPMed: Trans-Omics for Precision Medicine; URI: Uniform Resource Identifier; URL: Universal Resource Locator; W3C: World Wide Web Consortium; WMS: workflow management system.

## Competing interests

S.S.R. and M.R.C. are members of the leadership team for Common Workflow Language at the Software Freedom Conservancy.

## Funding

F.Z.K. is funded by Melbourne International Research Scholarship (MIRS) and Melbourne International Fee Remission Scholarship (MIFRS). S.S.R. and C.G. are funded by BioExcel CoE, a project funded by the European Commission Horizon 2020 Framework Programme under contracts H2020-INFRAEDI-02-2018-823830 and H2020-EINFRA-2015-1-675728, as well as IBISBA (H2020-INFRAIA-1-2014-2015-730976). MRC has received funding from the European Commission’s Horizon 2020 research and innovation programme under the Grant Agreement no 739563.

## Authors' contributions

Conceptualization: F.Z.K., S.S.R., M.R.C. Data curation: F.Z.K. Formal analysis: F.Z.K. Funding acquisition: R.O.S., A.L., C.A.G. Investigation: F.Z.K. Methodology: F.Z.K., S.S.R. Project administration: F.Z.K., S.S.R., R.O.S., A.L. Computing Resources: R.O.S., A.L. Software: F.Z.K., S.S.R., M.R.C. Supervision: M.R.C., R.O.S., A.L., C.A.G. Validation: F.Z.K., S.S.R. Writing—original draft: F.Z.K. Writing—review and editing: F.Z.K., S.S.R., R.O.S., A.L., M.R.C.

## Supplementary Material

giz095_GIGA-D-18-00483_Original_Submission

giz095_GIGA-D-18-00483_Revision_1

giz095_GIGA-D-18-00483_Revision_2

giz095_Response_to_Reviewer_Comments_Original_Submission

giz095_Response_to_Reviewer_Comments_Revision_1

giz095_Reviewer_1_Report_Original_Submission Tomoya Tanjo, Ph.D. -- 1/5/2019 Reviewed

giz095_Reviewer_1_Report_Revision_1 Tomoya Tanjo, Ph.D. -- 6/20/2019 Reviewed

giz095_Reviewer_2_Report_Original_Submission Alban Gaignard -- 1/22/2019 Reviewed

giz095_Reviewer_2_Report_Revision_1 Alban Gaignard -- 6/24/2019 Reviewed
