# Supplementary material for: Sharing interoperable workflow provenance: A review of best practices and their practical application in CWLProv
Source: Gigascience. 2019 Nov 1;8(11):giz095. doi: 10.1093/gigascience/giz095 (PMC6824458; doi:10.1093/gigascience/giz095)
Supplement: giz095_GIGA-D-18-00483_Revision_1 [file giz095_giga-d-18-00483_revision_1.pdf]

GigaScience

Sharing interoperable workflow provenance: A review of best practices and their practical application in CWLProv

--Manuscript Draft--

**Manuscript Number:**	GIGA-D-18-00483R1
**Full Title:**	Sharing interoperable workflow provenance: A review of best practices and their practical application in CWLProv
**Article Type:**	Research
**Funding Information:**	
**Abstract:**	Background: The automation of data analysis in the form of scientific workflows has become a widely adopted practice in many fields of research. Computationally driven data-intensive experiments using workflows enable Automation, Scaling, Adaption and Provenance support (ASAP). However, there are still several challenges associated with the effective sharing, publication and reproducibility of such workflows due to the incomplete capture of provenance and lack of interoperability between different technical (software) platforms.<br><br>Results: Based on best practice recommendations identified from literature on workflow design, sharing and publishing, we define a hierarchical provenance framework to achieve uniformity in the provenance and support comprehensive and fully re-executable workflows equipped with domain-specific information. To realise this framework, we present CWLProv, a standard-based format to represent any workflow-based computational analysis to produce workflow output artefacts that satisfy the various levels of provenance. We utilise open source community-driven standards; interoperable workflow definitions in Common Workflow Language (CWL), structured provenance representation using the W3C PROV model, and resource aggregation and sharing as workflow-centric Research Objects (RO) generated along with the final outputs of a given workflow enactment. We demonstrate the utility of this approach through a practical implementation of CWLProv and evaluation using real-life genomic workflows developed by independent groups.<br><br>Conclusions: The underlying principles of the standards utilised by CWLProv enable semantically-rich and executable Research Objects that capture computational workflows with retrospective provenance such that any platform supporting CWL will be able to understand the analysis, re-use the methods for partial re-runs, or reproduce the analysis to validate the published findings.
**Corresponding Author:**	Farah Zaib Khan<br>University of Melbourne<br>Melbourne, VIC AUSTRALIA
**Corresponding Author Secondary Information:**	
**Corresponding Author's Institution:**	University of Melbourne
**Corresponding Author's Secondary Institution:**	
**First Author:**	Farah Zaib Khan
**First Author Secondary Information:**	
**Order of Authors:**	Farah Zaib Khan
	Stian Soiland-Reyes
	Richard O. Sinnott
	Andrew Lonie
	Carole Goble
	Michael R. Crusoe

Powered by Editorial Manager® and ProduXion Manager® from Aries Systems Corporation

**Order of Authors Secondary Information:**	
**Response to Reviewers:**	Hi,<br>We have provided detailed responses in a pdf document (attached as supplementary material).<br>Please refer to that.<br>Cheers,
**Additional Information:**	
**Question**	**Response**
Are you submitting this manuscript to a special series or article collection?	No
**Experimental design and statistics**<br><br>Full details of the experimental design and statistical methods used should be given in the Methods section, as detailed in our Minimum Standards Reporting Checklist. Information essential to interpreting the data presented should be made available in the figure legends.<br><br>Have you included all the information requested in your manuscript?	Yes
**Resources**<br><br>A description of all resources used, including antibodies, cell lines, animals and software tools, with enough information to allow them to be uniquely identified, should be included in the Methods section. Authors are strongly encouraged to cite Research Resource Identifiers (RRIDs) for antibodies, model organisms and tools, where possible.<br><br>Have you included the information requested as detailed in our Minimum Standards Reporting Checklist?	Yes
**Availability of data and materials**<br><br>All datasets and code on which the conclusions of the paper rely must be either included in your submission or deposited in publicly available repositories	Yes

(where available and ethically appropriate), referencing such data using a unique identifier in the references and in the "Availability of Data and Materials" section of your manuscript.<br><br>Have you have met the above requirement as detailed in our Minimum Standards Reporting Checklist?	

Powered by Editorial Manager® and ProduXion Manager® from Aries Systems Corporation

Revised Manuscript
Click here to access/download;Manuscript;CWLProv.pdf
Click here to view linked References

OXFORD (GIGA)^n SCIENCE

GigaScience, 2018, 1–26

doi: 10.5281/zenodo.1966881
Revised manuscript submitted for publication
Research

RESEARCH

Sharing interoperable workflow provenance: A review of best practices and their practical application in CWLProv

Farah Zaib Khan[1,2,*], Stian Soiland-Reyes[2,3,*], Richard O. Sinnott[1,*], Andrew Lonie[1,*], Carole Goble[3,*] and Michael R. Crusoe[2,*]

[1]The University of Melbourne, Australia and [2]Common Workflow Language Project and [3]The University of Manchester, UK

[*]khanf1@unimelb.edu.au; soiland-reyes@manchester.ac.uk; rsinnott@unimelb.edu.au; alonie@unimelb.edu.au; carole.goble@manchester.ac.uk; mrc@commonwl.org

Abstract

Background: The automation of data analysis in the form of *scientific workflows* has become a widely adopted practice in many fields of research. Computationally driven data-intensive experiments using workflows enable Automation, Scaling, Adaption and Provenance support (ASAP). However, there are still several challenges associated with the effective sharing, publication and reproducibility of such workflows due to the incomplete capture of provenance and lack of interoperability between different technical (software) platforms.
Results: Based on best practice recommendations identified from literature on workflow design, sharing and publishing, we define a hierarchical provenance framework to achieve uniformity in the provenance and support comprehensive and fully re-executable workflows equipped with domain-specific information. To realise this framework, we present *CWLProv*, a standard-based format to represent any workflow-based computational analysis to produce workflow output artefacts that satisfy the various levels of provenance. We utilize open source community-driven standards; interoperable workflow definitions in Common Workflow Language (CWL), structured provenance representation using the W3C PROV model, and resource aggregation and sharing as workflow-centric Research Objects (RO) generated along with the final outputs of a given workflow enactment. We demonstrate the utility of this approach through a practical implementation of *CWLProv* and evaluation using real-life genomic workflows developed by independent groups.
Conclusions: The underlying principles of the standards utilized by *CWLProv* enable semantically-rich and executable Research Objects that capture computational workflows with retrospective provenance such that any platform supporting CWL will be able to understand the analysis, re-use the methods for partial re-runs, or reproduce the analysis to validate the published findings.

Key words: Provenance; Common Workflow Language; CWL; Research Object; RO; BagIt; Interoperability; Scientific Workflows; Containers

Introduction

Out of the many big data domains, genomics is considered "*the most demanding*" with respect to all stages of the data lifecycle - from acquisition, storage, distribution and analysis [1]. As genomic data is growing at an unprecedented rate due to improved sequencing technologies and reduced cost, it is currently challenging to analyse the data at a rate matching its production. With data growing exponentially in size and volume, the practice to perform computational analyses using *workflows*

Compiled on: May 23, 2019.
Draft manuscript prepared by the author.

Key Points
The contribution of this paper is fourfold:

- We have gathered best-practice recommendations from the existing literature, and reflect on the various authors' experiences with workflow managements systems and especially with regards to factors to consider when a computational analysis is designed, executed and shared.
- Combining the above with our own experiences from empirical studies [6, 7, 8, 9], we define a set of hierarchical levels of provenance tracking and method sharing where the highest level represent complete understanding of the shared resources supported by reproducibility and re-use of the methods from the lower levels.
- Building on this provenance hierarchy, we define *CWLProv* for the methodical representation of artefacts associated with a given workflow enactment associated with any study involving computational data-intensive analysis.
- Finally, we demonstrate the utilisation of *CWLProv* by extending an existing workflow execution engine *cwltool* [10] to produce workflow-centric Research Objects generated as a result of a given workflow enactment. We illustrate this through a case study of using workflows designed by external (independent) developers, and subsequently evaluate the interoperability, reproducibility and completeness of the generated *CWLProv* outcome.

has overtaken more traditional research methods using ad-hoc scripts which were the typical modus operandi over the last few decades [2, 3]. Scientific workflow design and management has become an essential part of many computationally driven data-intensive analyses enabling Automation, Scaling, Adaptation and Provenance support (ASAP)[4]. Increased use of workflows has driven rapid growth in the number of computational data analysis WMSs, with hundreds of heterogeneous approaches now existing for workflow specification and execution [5]. There is an urgent need for a common format and standard to define workflows and enable sharing of analysis results using a given workflow environment.

Common Workflow Language (CWL) [11] has emerged as a workflow definition standard designed to enable portability, interoperability and reproducibility of analyses between workflow platforms. CWL has been widely adopted by more than 20 organisations, providing an interoperable bridge overcoming the heterogeneity of workflow environments. Whilst a common standard for workflow definition is an important step towards interoperable solutions for workflow specifications, sharing and publishing the *results* of these workflow enactments in a common format is equally important. Transparent and comprehensive sharing of experimental designs is critical to establish trust and ensure authenticity, quality and reproducibility of any workflow-based research result. Currently there is no common format defined and agreed upon for interoperable workflow archiving or sharing [12].

In this paper, we utilize open-source standards such as CWL together with related efforts such as Research Objects (ROs) [13], BagIt [14] and PROV [15] to define ***CWLProv***, a format for the interoperable representation of a CWL workflow enactment. We focus on production of a workflow-centric executable RO as the final result of a given CWL workflow enactment. This RO is equipped with the artefacts used in a given execution including the workflow inputs, outputs and, most importantly, the retrospective provenance. This approach enables the complete sharing of a computational analysis such that any future CWL-based workflow can be re-run given the best practices discussed later for software environment provision are followed.

The concept of workflow-centric ROs has been previously considered [13, 16, 17] for structuring the analysis methods and aggregating digital resources utilized in a given analysis. The generated ROs in these studies typically aggregate data objects, example inputs, workflow specifications, attribution details, details about the execution environment amongst various other elements. These previous efforts were largely tied to a single platform or a single Workflow Management System (WMS). *CWLProv* aims to provide a platform-independent solution for workflow sharing, enactment and publication. All the standards and vocabularies used to design *CWLProv* have an overarching goal to support a domain-neutral and interoperable solution (detailed in Section **Applied Standards and Vocabularies**).

The contribution of this work are summarized and listed in the **Key Points** section and the remainder of this paper is structured as follows. In Section **Background and Related Work** we discuss the key concepts and related work followed by a summary of the published best-practices and recommendations for workflow representation and sharing in Section **Levels of Provenance and Resource Sharing**. This section also details the hierarchical provenance framework that we define to provide a principled approach for provenance capture and method sharing. Section **CWLProv 0.6.0 and utilized standards** introduces *CWLProv* and outlines its format, structure and the details of the standards and ontologies it utilizes. Section **Practical Realisation of *CWLProv*** presents the implementation details of *CWLProv* using *cwltool* [10] and Section **CWLProv Evaluation with Bioinformatics Workflows** demonstrates and evaluates the implemented module for three existing workflow case studies. We discuss the challenges of interoperable workflow sharing and the limitations of the proposed solution listing several possible future research directions in Section **Discussion and Future Directions** before finally drawing conclusions on the work as a whole in Section **Conclusion**.

Background and Related Work

This work draws upon a range of topics as *Provenance* and *Interoperability*. We define these here to provide better context for the reader.

Provenance

A number of studies have advocated the need for complete provenance tracking of scientific workflows to ensure transparency, reproducibility, analytic validity, quality assurance and attribution of (published) research results [18]. The term *Provenance* is defined by World Wide Web Consortium (W3C) [19] as:

> *"Provenance is information about entities, activities, and people involved in producing a piece of data or thing, which can be used to form assessments about its quality, reliability or trustworthiness."*

Provenance for workflows is commonly divided into the following three categories: *Retrospective Provenance*; *Prospective Prove-*

nance and *Workflow Evolution*. *Retrospective Provenance* refers to the detailed record of the implementation of a computational task including the details of every executed process together with comprehensive information about the execution environment used to derive a specific product. *Prospective Provenance* refers to the 'recipes' used to capture a set of computational tasks and their order, e.g. the workflow specification [20]. This is typically given as an abstract representation of the steps (tools/data analysis steps) that are necessary to create a particular research output, e.g. a data artefact. *Workflow Evolution* refers to tracking of any alteration in the existing workflow resulting in another version of the workflow that may produce either the same or different resultant data artefacts [21]. In this work, our focus is mainly on improving representation and capture of *Retrospective Provenance*.

Interoperability

The concept of interoperability varies in different domains. Here we focus on *computational interoperability* defined as:

> *The ability of two or more components or systems to exchange information and to use the information that has been exchanged* [22].

The focus of this study is to propose and devise methods to achieve *syntactic*, *semantic* and *pragmatic* interoperability as defined in Levels of Conceptual Interoperability Model (LCIM)[23]. *Syntactic* interoperability is achieved when a common data format for information exchange is unambiguously defined. The next level of interoperability, referred to as *semantic* interoperability, is reached when the content of the actual information exchanged is unambiguously defined. Once there is an agreement about the format and content of the information, *pragmatic* interoperability is achieved when the context, application and use of the shared information and data exchanged is also unambiguously defined. In the section **Evaluation Results**, we relate these general definitions to specific workflow applications with respect to workflow-centric ROs and describe to what extent these interoperability requirements are addressed.

Related Work

We focus on relevant studies and efforts trying to resolve the issue of availability of required resources used in a given computational analysis. In addition, we cover efforts directed towards provenance capture of workflow enactments. As these concepts have been around for a considerable time, we restrict our attention to scientific workflows and studies related to the bioinformatics domain.

Workflow Software Environment Capture

Freezing and packaging the run-time environment to encompass all the software components and their dependencies used in an analysis is a recommended and widely adopted practice [24] especially after use of cloud computing resources where images and snapshots of the cloud instances are created and shared with fellow researchers [25]. Nowadays, preservation and sharing of the software environment e.g. in open access repositories, is becoming a regular practice in the workflow domain as well. Leading platforms managing infrastructure and providing cloud computing services and configuration on demand include DigitalOcean [26], Amazon Elastic Compute Cloud [27], Google Cloud Platform [28] and Microsoft Azure [29]. The instances launched on these platforms can be saved as snapshots and published with an analysis study to later recreate an instance representing the computing state at analysis time.

Using *"System-wide packaging"* for data-driven analyses, although simplest on part of the workflow developers and researchers, has its own caveats. One of the notable issue is the size of the snapshot as it captures everything in an instance at a given time, hence the size can range from few gigabytes to many terabytes. To distribute research software and share execution environments, various light-weight and container-based virtualisation and package managers are emerging, including: Docker, Singularity, Debian Med and Bioconda.

Docker[30] is a lightweight container-based virtualisation technology that facilitates the automation of application development by archiving software systems and environment to improve portability of the applications on many common platforms including Linux, Microsoft windows, Mac OS X and cloud instances. *Singularity*[31] is also a cross-platform open source container engine specifically supporting High Performance Computing (HPC) resources. An existing Docker format software image can be imported and used by the Singularity container engine. *Debian Med* [32] contribute packages of medical practice and biomedical research to the Debian Linux distribution, lately also including workflows [8]. *Bioconda*[33] packages, based on the an open source package manager Conda [34], are available for Mac OS X and Linux environments, directing towards availability and portability of software used in the life science domain.

Data/Method Preservation, Aggregation & Sharing

Preserving and sharing only the software environment is not enough to verify results of any computational analysis or reuse the methods used (e.g. workflows) with a different dataset. It is also necessary to share other details including data (example or the original), scripts, workflow files, input configuration settings, the hypothesis of the experiment and any/all trace/logging information related to "what happened", i.e. the retrospective provenance of the actual workflow enactment. The publishing of resources to improve state of scholarly publications is now supported by various online repositories, including Zenodo [35], GitHub [36], myExperiment [37] and Figshare [38]. These repositories facilitate collaborative research, in addition to public sharing of source code and the results of a given analysis. There is however no agreed format that must be followed when someone shares artefacts associated with an analysis. As a result, the quality of the shared resources can range from a highly annotated, properly documented and complete set of artefacts, to raw data with undocumented code and incomplete information about the analysis as a whole. Individual organisations or groups might provide a set of "recommended practices", e.g. in readme files, to attempt to maintain the quality of shared resources. The initiative *Code as a Research Object* [39] is a joint project between Figshare, GitHub and Mozilla Science Lab [40] and aims to archive any GitHub code repository to Figshare and produce a Digital Object Identifier (DOI) to improve the discovery of resources[1].

Reprozip[41] aims to resolve portability issues by identifying and packaging all dependencies in a self-contained package which when unpacked and executed on another system (with Reprozip installed) should reproduce the methods and results of the analysis. Each package also contains a human readable configuration file containing provenance information obtained by tracing system calls during system execution. The corresponding provenance trace is however not formatted using existing open standards established by the community. Several platform-dependent studies have been targeted towards extensions to existing standards by implementing the Research Ob-

1 For the source code that support this work we have used a similar publishing feature with Zenodo.

ject model and improving aggregation of resources. Belhajjame et al. [13] proposed the application of ROs to develop workflow-centric ROs containing data and metadata to support the understandability of the utilized methods (in this case workflow specifications). They explored five essential requirements to workflow preservation and identified data and metadata that could be stored to satisfy the said requirements. These requirements include providing example data, preserving workflows with provenance traces, annotating workflows, tracking the evolution in workflows and packaging the auxiliary data and information with workflows. They proposed extensions to existing ontologies such as Object Reuse and Exchange (ORE), the Annotation Ontology (AO) and PROV-O, with four additional ontologies to represent workflow specific information. However, as stated in the paper, the scope of the proposed model at that time was not focused on interoperability of heterogeneous workflows as it was demonstrated for a workflow specific to Taverna WMS using myExperiment, which makes it quite platform-dependent.

A domain-specific solution is proposed by Gomez-Perez et al. [42] by extending the RO model to equip workflow-centric ROs with information catering for the specific needs of the Earth Science community, resulting in enhanced discovery and reusability by experts. They demonstrated that the principles of ROs can support extensions to generate aggregated resources leveraging domain specific knowledge. Hettne et al. [16] used three genomic workflow case studies to demonstrate the utilisation of ROs to capture methods and data supporting querying and useful extraction of information about the scientific investigation under observation. The solution was tightly coupled with the Taverna WMS and hence if shared, would not be reproducible outside of the Taverna environment. Other notable efforts to use ROs for workflow preservation and method aggregation include [7] in systems biology, [43] in clinical settings and [9] in precision medicine.

Provenance Capture & Standardization

A range of standards for provenance representation have been proposed. Many studies have emphasized the need for provenance focusing on aspects such as scalability, granularity, security, authenticity, modelling and annotation [18]. They identify the need to support standardized dialogues to make provenance interoperable. Many of these were used as inputs to initial attempts at creating a standard Provenance Model to tackle the often inconsistent and disjointed terminology related to provenance concepts. This ultimately resulted in the specification of the *Open Provenance Model* (OPM)[44] together with an open-source model for the governance of OPM [45]. Working towards similar goals of interoperability and standardization of provenance for web technologies, the World Wide Web Consortium (W3C) Provenance Incubator Group [46] and the authors of OPM together set the fourth provenance challenge at the International Provenance and Annotation Workshop, 2010 (IPAW'10) that later resulted in *PROV*, a family of documents serving as the conceptual model for provenance capture, its representation, sharing and exchange over the Web [47] regardless of the domain or platform. Since then, a number of studies have proposed extensions to this domain-neutral standard. The model is general enough to be adapted to any field and flexible enough to allow extensions for specialized cases.

Michaelides et al. [48] presented a domain-specific PROV-based solution for retrospective provenance to support portability and reproducibility of a statistical software suite. They captured the essential elements from the log of a workflow enactment and represented them using an intermediate notation. This representation was later translated to PROV-N and used as the basis for the PROV Template System. A Linux specific system provenance approach was proposed in [49] where they demonstrated retrospective provenance capture at the system level. Another project *UniProv* is working to extract information from Unicore middleware and transform it into a PROV-O representation to facilitate the back-tracking of workflow enactments [50]. Other notable domain-specific efforts leveraging the established standards to record provenance and context information are *PROV-man* [51], PoeM [52] and micropublications [53]. Platforms such as VisTrails and Taverna have built in retrospective provenance support. *Taverna* [7] implements an extensive provenance capture system *TavernaProv*[54], utilising both PROV ontologies as well as ROs aggregating the resources used in an analysis. *VisTrails*[55] is an open source project supporting platform-dependent provenance capture, visualisation and querying for extraction of required information about a workflow enactment. [41] provide an overview of PROV terms and how they can be translated from the VisTrails schema and serialized to PROV-XML. *WINGS*[56] can report fine-grained workflow execution provenance as Linked Data using the OPMW ontology [57], which builds on both PROV-O and OPM.

All these efforts are fairly recent and use a standardized approach to provenance capture and hence are relevant to our work on the capture of retrospective provenance. However, our aim is a domain-neutral and platform-independent solution that can be easily adapted for any domain and shared across different platforms and operating systems.

As evident from the literature, there are efforts in progress to resolve the issues associated with effective and complete sharing of computational analysis including both the results and provenance information. These studies range from highly domain-specific solutions and platform-dependent objects to open source flexible interoperable standards. CWL has widespread adoption as a workflow definition standard, hence is an ideal candidate for portable workflow definitions. The next section investigates existing studies focused on workflow-centric science, and summarises best practice recommendations put forward in these studies. From this we define a hierarchical provenance and resource sharing framework.

Levels of Provenance and Resource Sharing

Various studies have empirically investigated the role of automated computational methods in the form of workflows and published best practice recommendations to support workflow design, preservation, understandability and re-use. We summarise a number of these recommendations and the their justifications in Table 1, where each recommendation addresses specific requirement of workflow design and sharing. These recommendations can be clustered into broad themes as shown in Figure 1. This classification can be in more than one way e.g. according to how these recommendations are supporting each FAIR dimension [67]. In this study, we have focused on categories with respect to workflow design, prospective provenance, data sharing, retrospective provenance, the computational environment required/used for an analysis and lastly better findability and understandability of all shared resources.

Sharing *"all artefacts"* from a computational experiment (following all recommendations and best practices) is a demanding task without any informed guidance. It requires consolidated understanding of the impact of the many different artefacts involved in that analysis. This places extra efforts on workflow designers, (re)-users, authors, reviewers and expectations on the community as a whole. Given the numerous WMS and differences in how each system deals with provenance documentation, representation and sharing of these artefacts, the granularity of provenance information preserved will vary for each workflow definition approach. Hence, devising one universal

Table 1. Summarized recommendations and justifications from literature covering best practices on reproducibility, accessibility, interoperability and portability of workflows

R.no	Recommendations	Justifications
R1 parameters	Save and share all parameters used for each software executed in a given workflow (including default values of parameters used) [58, 59, 57, 60].	Impacts on reproducibility of results since different inputs and configurations of the software can produce different results. Different versions of a tool might upgrade the default values of the parameters.
R2 automate	Avoid manual processing of data and if using *shims* [61] then make these part of the workflow to fully automate the computational process [58, 60].	This ensures the complete capture of the computational process without broken links so that the analysis can be executed without need for performing manual steps.
R3 intermediate	Include intermediate results where possible when publishing an analysis [59, 57, 60].	Intermediate data products can be used to inspect and understand shared analysis when re-enactment is not possible.
R4 sw-version	Record the exact software versions used [58, 60].	This is necessary for reproducibility of results as different software versions can produce different results.
R5 data-version	If using public data (reference data, variant databases), then it is necessary to store and share the actual data versions used [3, 6, 58, 60] .	This is needed as different versions of data, e.g. human reference genome or variant databases, can result in slightly different results for the same workflow.
R6 annotation	Workflows should be well-described, annotated and offer associated metadata. Annotations such as user contributed tags and versions should be assigned to workflows and shared when publishing the workflows and associated results [13, 17, 57, 62, 63] .	Metadata and annotations improve the understandability of the workflow, facilitate independent re-use by someone skilled in the field, make workflows more accessible and hence promote the longevity of the workflows.
R7 identifier	Use and store stable identifiers for all artefacts including the workflow, the datasets and the software components [62, 63].	Identifiers play an important role in the discovery, citation and accessibility of resources made available in open-access repositories.
R8 environment	Share the details of the computational environment [13, 6, 63] .	Such details support requirements analysis before any re-enactment or reproducibility is attempted.
R9 workflow	Share workflow specifications/descriptions used in the analysis [13, 59, 57, 63, 64].	The same workflow specifications can be used with different datasets thereby supporting re-usability.
R10 software	Aggregate the software with the analysis and share this when publishing a given analysis [13, 6, 63, 64, 57].	Making software available reduces dependence on third party resources and as a result minimizes *workflow decay* [65].
R11 raw-data	Share raw data used in the analysis [13, 59, 57, 63, 64].	When someone wants to validate published results, availability of data supports verification of claims and hence establishes trust in the published analysis
R12 attribution	Store all attributions related to data resources and software systems used [57, 64].	Accreditation supports proper citation of resources used.
R13 provenance	Workflows should be preserved along with the provenance trace of the data and results [13, 17, 57, 60, 64].	A provenance trace provides a historical view of the workflow enactment, enabling end users to better understand the analysis retrospectively
R14 diagram	Data flow diagrams of the computational analysis using workflows should be provided [6, 59].	These diagrams are easy to understand and provide a human readable view of the workflow.
R15 open-source	Open source licensing for methods, software, code, workflows and data should be adopted instead of proprietary resources [6, 59, 60, 63, 64, 66].	This improve availability and legal re-use of the resources used in the original analysis, while restricted licenses would hinder reproducibility.
R16 format	Data, code and all workflow steps should be shared in a format that others can easily understand preferably in a system neutral language [13, 59, 66].	System neutral languages help achieve interoperability and make an analysis understandable.
R17 executable	Promote easy execution of workflows without making significant changes to the underlying environment [3].	In addition to helping reproducibility, this enables adapting the analysis methods to other infrastructures and improves workflow portability.
R18 resource-use	Information about compute and storage resources should be stored and shared as part of the workflow [6].	Such information can assist users in estimating the required resources needed for an analysis and thereby reduce the amount of failed executions.
R19 example	Example input and sample output data should be preserved and published along with the workflow-based analysis [13, 65].	This information enables more efficient test runs of an analysis to verify and understand the methods used.

This list is not exhaustive, other studies have identified separate issues (e.g. lab work provenance and data security) that are beyond the scope of this work.

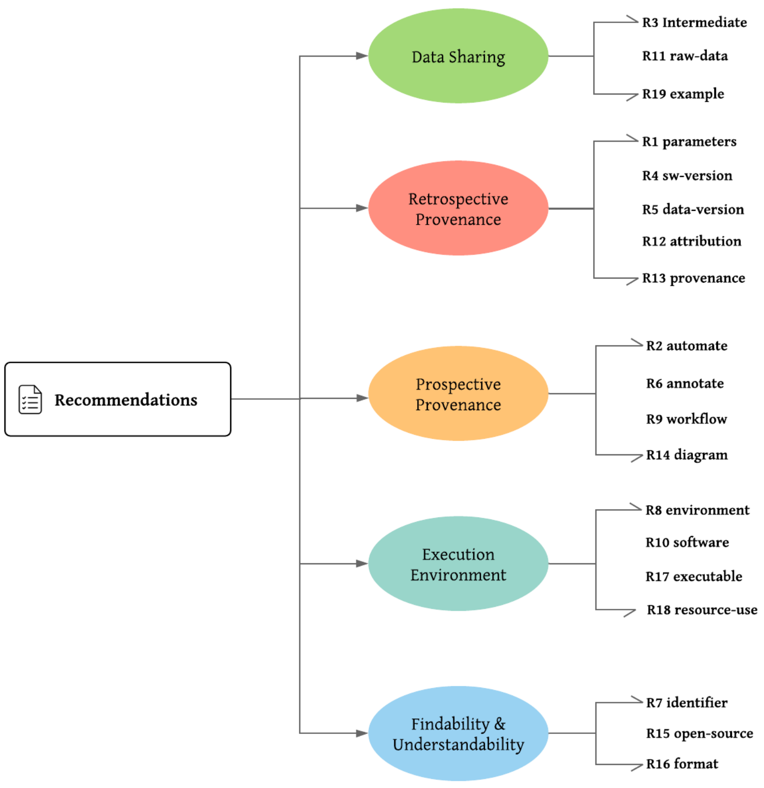

Figure 1. Recommendations from Table 1 classified into these categories

but technology-specific solution for provenance capture and the related resource sharing is impossible. Instead we propose a generic framework of provenance in Figure 2 that all WMSs can benefit from and conform to with minimum technical overheads.

The recommendations in Table 1 aid in our understanding to define this framework by classifying the granularity of the provenance and related artefacts where the uppermost level exhibits comprehensive, reproducible, understandable and provenance-rich computational experiment sharing. The purpose of this framework is threefold. First, because of its generic nature it brings the uniformity in the provenance granularity across various WMS belonging to different workflow definition approaches. Second, it provides comprehensive and well-defined guidelines that can be used by the researchers to conduct principled analysis of the provenance of any published study. Third, due to its hierarchical nature, the framework can be leveraged by the workflow authors to progress incrementally towards the most transparent workflow-centric analysis. Overall, this framework will help achieve a uniform level of provenance and resource sharing with a given workflow-centric analysis guaranteed to fulfill the respective provenance applications.

Our proposed provenance levels are ordered from low granularity to higher degrees of specificity. In brief, **Level 0** is unstructured information about the overall workflow enactment, **Level 1** adds structured retrospective provenance, access to primary data and executable workflows, **Level 2** enhances the white-box provenance for individual steps, and ***Level 3*** adds domain-specific annotations for improved understanding. These levels are described in the following sub-sections and mapped to the requirements in Table 1 that these levels aim to satisfy.

Level 0

To achieve this level, researchers should share the workflow specifications, input parameters used for a given workflow enactment, raw logs and output data preferably through an open-access repository. This is the least information that could be shared without putting any extra efforts to support seamless reuse or understandability of a given analysis. The artefacts shared at this level would only require uploading of the associated resources to a repository without necessarily providing any supporting metadata or provenance information. Information captured at *Level 0* is the bare minimum that can be used for result interpretation.

Workflow definitions based on *Level 0* can also potentially be re-purposed for other analyses. As argued by Ludäscher, a well-written scientific workflow and its graphical representation is itself a source of prospective provenance giving user an idea of the steps taken and data produced [68]. Therefore a well-described workflow specification indirectly provides prospective provenance without aiming for it. In addition to the textual workflow specification, its graphical representation should also be shared if available for better understandability fulfilling *R14-diagram*. At this level, reproducing the workflow would only be possible if the end-user devotes extra efforts to understand the shared artefacts and carefully recreate the execution environment. As open access journals frequently require availability of methods and data, many published studies now share workflow specifications and optionally the outputs thereby achieving *Level 0* and specifically sat-

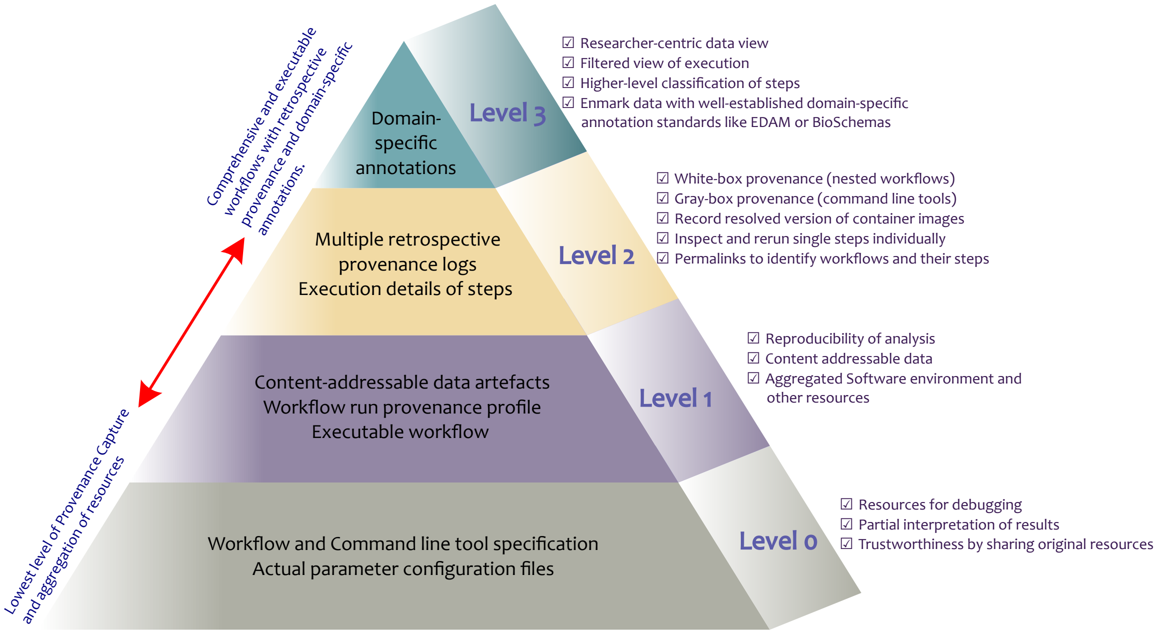

Figure 2. Levels of Provenance and resource sharing and their applications

isfying *R1-parameters* and *R9-workflow* (Table 1). In addition, the resources shared should have open licence starting from *Level 0* and this practice proposed by *R15-open-source* should be adopted at each higher level.

Level 1

At *Level 1*, *R4-sw-version*, *R5-data-version*, *R12-attribution* and *R13-provenance* should be satisfied by providing retrospective provenance of the workflow enactment – i.e. a structured representation of machine readable provenance which can answer questions such as "what happened", "when happened", "what was executed", "what was used", "who did this" and "what was produced". Seamless re-enactment of the workflow should be supported at this level. This is only possible when along with provenance information, *R8-environment* and *R10-software* is satisfied by potentially packaging the software environment for analysis sharing or there is enough information about the software environment that guide the user to reliably re-enact the workflow. Hence *R17-executable* should be satisfied making it possible for the end users to re-enact the shared analyses without making major changes to the underlying software environment.

In addition to the software availability and retrospective provenance, access to input data should also be provided fulfilling *R11-raw-data*. This data can be used to re-enact the published methods or utilized in a different analysis, e.g. for performance comparison of methods. At *Level 1*, it is preferable to provide content-addressable data artefacts such as input, output and intermediate files, avoiding local paths and file names to make a given workflow executable outside its local environment. The intermediate data artefacts should also be provided to facilitate inspection of all step results, hence satisfying *R3-intermediate*. All resources, including workflow specifications and provenance, should be shared in a format that is understandable across platforms, preferably in a technology-neutral language as proposed by *R16-format*.

While software and data can be digitally captured, the hardware and infrastructure requirements also need to be captured to fulfill *R18-resource-use*. This kind of information can naturally vary widely with runtime environments, architectures and data sizes [69], as well as rapidly becoming outdated as hardware and cloud offerings evolve. Nevertheless a snapshot of the workflow's overall execution resource usage for an actual run can be beneficial to give a broad overview of the requirements, and can facilitate cost-efficient re-computation by taking advantage of spot-pricing for cloud resources [70].

Level 2

It is a common practice in scientific workflows to modularize the workflow specifications by separating the related tasks into "sub-workflows" or "nested workflows" [24] to be incorporated and used in other workflows or be assigned to compute and storage resources in case of distributed computing [71]. These modular solutions promote understanding and reusability of the workflows as researchers are inclined to use these modules instead of workflow as whole for their own computational experiments. An example of a sub-workflow is the mandatory "pre-processing" [72] needed for the Genome Analysis ToolKit (GATK) best practice pipelines used for genomic variant calling. These steps can be separated into a sub-workflow to be used before any variant calling pipeline, be it somatic or germline.

At *Level 1*, retrospective provenance is coarse grained and as such, there is no distinction between workflows and their sub-workflows. Ludäscher [68] distinguishes workflow provenance between *black-box* and database provenance as *white-box*. The reasoning behind this distinction is that often the steps in a workflow, especially those based on graphical user interface-based platforms, provide levels of abstraction/obscurity to the actual tasks being implemented. In our previous work we used an empirical case study to demonstrate that declarative approaches to workflow definition resulted in transparent workflows with the least number of assumptions [6]. This resolves the black box/white box issue to some extent, but to further support research transparency, we propose to share retrospective provenance logs for each nested/sub-workflow making the details of a workflow enactment as explicit as possible and moving a step closer to *white-box* provenance. These provenance logs will support the inspection and automatic re-enactment of targeted components of a workflow such as a

single step or a sub-workflow individually without necessarily having to re-enact the full analysis. Some existing make-like systems such as Snakemake support partial re-enactments but typically rely on fixed file paths for input data and require manual intervention to provide the specific directory structure. With detailed provenance logs and the corresponding content-addressable data artefacts, the partial re-runs can be achieved with automatic generation of input configuration setting.

In addition, we propose to include *permalinks* at *Level 2* to identify the workflows and their individual steps which facilitates the inspection of each step and aim to improve the longevity of the shared resources, hence supporting *R7-identifier*. Improving *R18-resource-use* for *Level 2* would include resource usage per task execution. Along with execution times this can be useful information to identify bottlenecks in a workflow and for more complex calculations in cost optimization models [73]. At this provenance level resource usage data will however also become more noisy and highly variant on scheduling decisions by the workflow engine, e.g. sensitivity to cloud instance reuse or co-use for multiple tasks, or variation in data transfers between tasks on different instances. Thus *Level 2* resource usage information should be further processed with statistical models for it to be meaningful for a user keen to estimate the resource requirement for re-enactment of a given analysis.

Level 3

Levels 0-2 are generic and domain-neutral, and can apply to any scientific workflow. However, domain-specific information/metadata about data and processes plays an important role in better understanding of the analysis and exploitation of provenance information, e.g. for meaningful queries to extract information to the domain under consideration [74, 75]. Addition of domain specific metadata e.g. file formats, user-defined tags and other annotations to generic retrospective provenance can improve the *white-boxness* by providing domain context to the analysis as described in *R6-annotations*. Annotations can range from adding textual description and tags to marking data with more systematic and well-defined domain-specific ontologies such as EDAM [76] and BioSchemas [77] in the case of bioinformatic workflows. Some studies also propose to provide example or test data sets which eventually helps in analyzing the methods shared and verifying their results (as described in *R19-example*).

At *Level 3*, the information from previous levels combined with specific metadata about data artefacts facilitates higher level classification of workflow steps into *motifs* [78] such as data retrieval, pre-processing, analysis and visualisation. This level of provenance, resource aggregation and sharing can provide a researcher-centric view of data and enable users to re-enact a set of steps or full workflow by providing filtered and annotated view of the execution. This can be non-trivial to achieve with mainstream methods of workflow definition and sharing, as it requires guided user annotations with controlled vocabularies, but this can be simplified by reusing related tooling from existing efforts like BioCompute Objects [9] and DataCrate [79].

Communicating resource requirements (*R18-resource-use*) at *Level 3* would involve domain-specific models for hardware use and cost prediction, as suggested for dynamic cloud costing [80] in *BioSimSpace* [81], or predicting assembler and memory settings through machine learning of variables like source biome, sequencing platform, file size, read count and base count in the *European Bionformatics Institute (EBI) Metagenomics* pipeline [82]. For robustness such models typically need to be derived from resource usage across multiple workflow runs with varied inputs, e.g. by a multi-user workflow platform. Taking advantage of *Level 3* resource usage models might require pre-processing workflow inputs and calculations in an environment like R or Python, and so we recommend that models are provided with separate sidecar workflows for interoperable execution before the main workflow.

By explicit enumeration of the levels of provenance, it should be possible to quantify and directly assess the effort required to re-use a workflow and reproduce experiments directly. Similar effort like *5-star Open Data* [83] strongly advocates open-licensed structured representation, use of stable identifiers for data sharing and following Linked Data principles to cross-relate data. One challenge on achieving the Open Data stars is that it needs tool support during data processing. In our framework we proposed systematic workflow-centric resource sharing using structured Linked Data representation, including recording of the executed data operations. Hence, our effort compliments the already proposed 5-star Open Data principles and contributes to further understanding by sharing the computational method following the same principles.

Requiring researchers to achieve the above defined levels individually is unrealistic without guidance and direct technical support. Ideally, the conceptual meaning of these levels would be translated into a practical solution utilising the available resources. However, given the heterogeneity of workflow definition approaches, it is expected that the proposed framework, when translated into practical solutions, will also naturally result in varying workflow-centric solutions tied to specific WMSs. To support interoperability of the workflow-centric analysis achieving the provenance levels, we propose ***CWLProv***, a format for annotating resource aggregations equipped with retrospective provenance. The next section describes *CWLProv* and the associated standards that are applied in this process.

CWLProv 0.6.0 and utilized standards

Here we present *CWLProv*, a format for the methodical representation of workflow enactment, associated artefacts and capturing and using retrospective provenance information. Keeping in view the recommendations from Table 1 for example *R15-open-source* and *R16-format*, we leverage **open-source**, **domain-independent**, **system-neutral**, **interoperable** and most importantly **community-driven** standards as the basis for the design and formatting of reproducible and interoperable workflow-based ROs. The profile description in this section correspond to *CWLProv* 0.6.0 [84]. (see https://w3id.org/cwl/prov for the latest profile).

Applied Standards and Vocabularies

We follow the recommendation *"Reuse vocabularies, preferably standardized ones"* [85] from best practices associated with data sharing, representation and publication on the web to achieve consensus and interoperability of workflow-based analyses. Specifically we integrate the *Common Workflow Language* (CWL) for workflow definition, *Research Objects* (ROs) for resource aggregation and the *PROV-Data Model* (PROV-DM) to support the retrospective provenance associated with workflow enactment. The key properties and principles of these standards are described below.

Common Workflow Language (CWL)

Common Workflow Language [11] provides declarative constructs for workflow structure and command line tool interface definition. It makes minimal assumptions about base software dependencies, configuration settings, software versions,

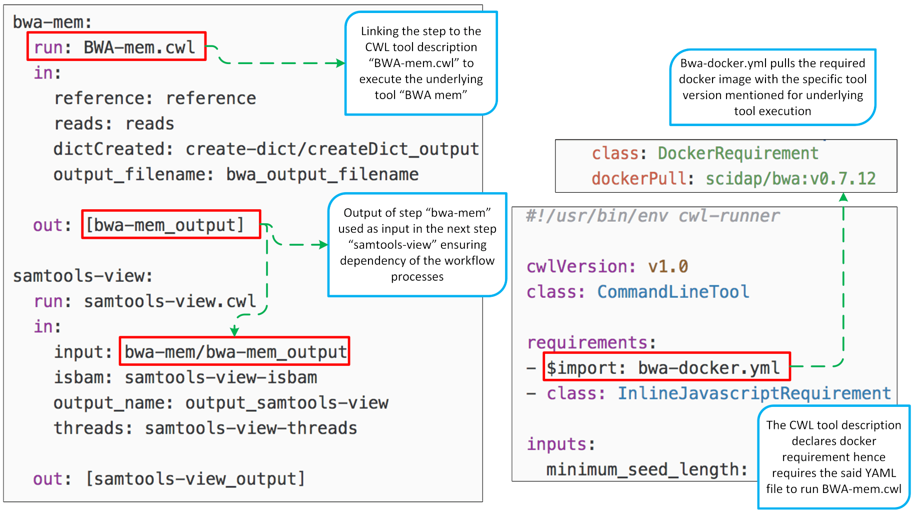

Figure 3. Left: A snapshot of part of a GATK workflow described using CWL. Two steps named as *bwa-mem* and *samtools-view* are shown where the former links to the tool description executing the underlying tool (BWA-mem for alignment) and provides the output used as input for samtools. Right: Snapshot of BWA-mem.cwl and the associated Docker requirements for the exact tool version used in the workflow execution.

parameter settings or indeed the execution environment more generally [6]. The CWL object model supports comprehensive recording and capture of information for workflow design and execution. This can subsequently be published as structured information alongside any resultant analysis using that workflow.

CWL is a community-driven standard effort that has been widely adopted by many workflow design and execution platforms, supporting interoperability across a set of diverse platforms. Current adopters include Toil, Arvados, Rabix [86], Cromwell [87], REANA, and Bcbio [88] with implementations for Galaxy, Apache Taverna, and AWE currently in progress.

A workflow in CWL is composed of "steps" where each step refers either to a command line tool (also specified using CWL) or another workflow specification incorporating the concept of "sub-workflows". Each "step" is associated with "inputs" that are comprised of any data artefact required for the execution of that step (Figure 3). As a result of the execution of each step, "outputs" are produced which can become (part of) "inputs" for the next steps making the execution data-flow oriented. CWL is not tied to a specific operating system or platform which makes it an ideal approach for interoperable workflow definitions.

Research Object (RO)

A Research Object encapsulates all of the digital artefacts associated with a given computational analysis contributing towards preservation of the analysis [89], together with their metadata, provenance and identifiers.

The aggregated resources can include but are not limited to: input and output data for analysis results validation; computational methods such as command line tools and workflow specifications to facilitate workflow re-enactment; attribution details regarding users; retrospective as well as prospective provenance for better understanding of workflow requirements, and machine-readable annotations related to the artefacts and the relationships between them. The goal of ROs is to make any published scientific investigation and the produced artefacts *"interoperable, reusable, citable, shareable and portable"*.

The three core principles [90] of the RO approach are to support "Identity", "Aggregation", and "Annotation" of research artefacts. They look to enable accessibility of tightly-coupled, interrelated and well-understood aggregated resources involved in a computational analysis as identifiable objects, e.g. using unique (persistent) identifiers such as DOIs and/or ORCIDs. The RO approach is well aligned with the idea of interoperable and platform-independent solutions for provenance capture of workflows because of its domain-neutral and platform-independent nature.

While ROs can be serialized in several different ways, in this work we have reused the BDBag approach based on *BagIt* (see box), which has been shown to support large-scale workflow data [91]. This approach is also compatible with data archiving efforts from the NIH Data Commons, Library of Congress and the Research Data Alliance. The specialized workflow-centric RO in this study encompasses the components mentioned in the previous paragraph annotated with various targeted tools and a PROV-based *Workflow provenance profile* to capture the detailed retrospective provenance of the CWL workflow enactment.

PROV Data Model (PROV-DM)

The World Wide Web Consortium (W3C) developed *PROV*, a suite of specifications for unified/interoperable representation and publication of provenance information on the Web. The underlying conceptual PROV Data Model (PROV-DM) [19] provides a domain-agnostic model designed to capture fundamental features of provenance with support for extensions to integrate domain-specific information (Figure 4).

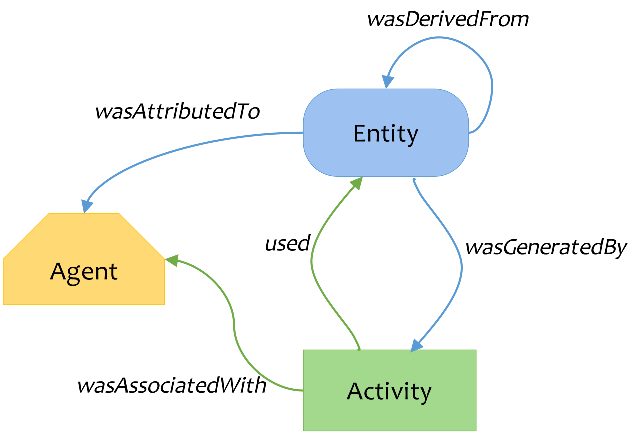

Figure 4. Core concepts of the PROV Data Model. Adapted from W3C PROV Model Primer [92].

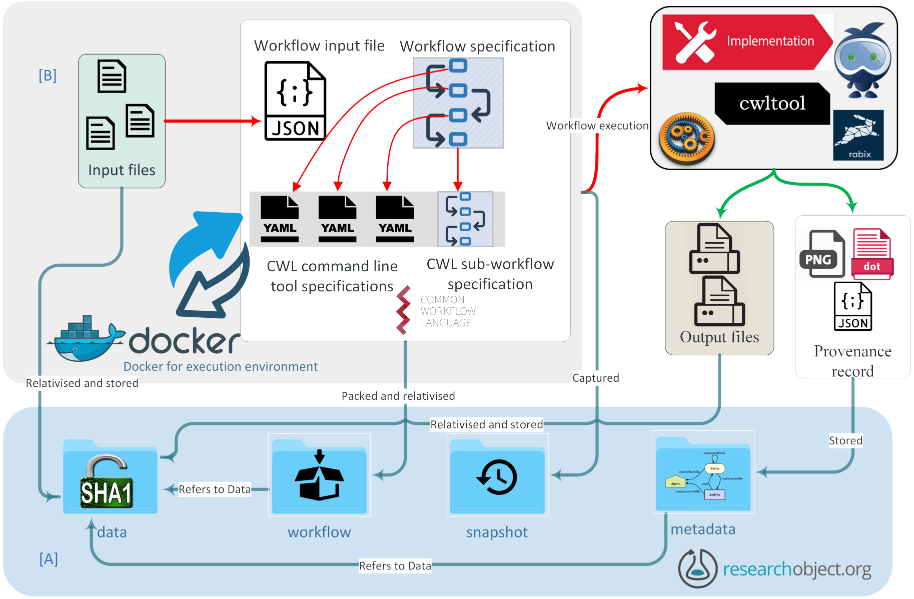

Figure 5. Schematic representation of the aggregation and links between the components of a given workflow enactment. Layers of execution are separated for clarity. The workflow specification and command line tool specifications are described using CWL. Each individual command line tool specification can optionally interact with Docker to satisfy software dependencies. [A] The RO layer shows the structure of the RO including its content and interactions with different components in the RO and [B] the CWL layer.

We utilize mainly two serialisations of PROV for this study, PROV-Notation (PROV-N) [93] and PROV-JSON [94]. PROV-N is designed to achieve serialisation of PROV-DM instances by formally representing the information using a simplified textual syntax to improve human readability. PROV-JSON is a lightweight interoperable representation of PROV assertions using JavaScript constructs and data types. The key design and implementation principles of these two serialisations of PROV are in compliance with the goals of this study, i.e. understandable and interoperable, hence are a natural choice to support the design of an adaptable provenance profile. For completeness we also explored serializing the provenance graph as PROV-XML [95] as well as PROV-O [96], which provides a mapping to Linked Data and ontologies, with potential for rich queries and further integration using a triple store. One challenge here is the wide variety of OWL and RDF formats, we opted for Turtle, N-Triples and JSON-LD, but concluded that requiring all of these PROV and RDF serializations would be an unnecessary burden for other implementations of *CWLProv*.

CWLProv Research Object

The provenance framework defined in previous section can be satisfied by using a structured approach to share the identified resources. In this section, we define the representation of data and metadata to be shared for a given workflow enactment, stored as multiple files in their native formats. The folder structure of the *CWLProv* Research Object complies with the *BagIt* [14] format such that its content and completeness can be verified with any BagIt tool or library (see box **What is BagIt?**). The files used and generated by the workflow are here considered the *data payload*; the remaining directories include *metadata* of how the workflow results were created. We systematized the aggregated resources into various collections for better understanding and accessibility for a CWL workflow execution (Figure 5).

data/

`data/` is the *payload* collection of the Research Object, in *CWLProv* this contains all input and output files used in a given workflow enactment. Data files should be labelled and identified based on a hashed checksum rather than derived from its file path during workflow execution. This use of *content-addressable* reference and storage [97] simplifies identifier generation for data and helps to avoid local dependencies, e.g. hard-coded file names. However, the workflow execution engine might use other unique identifiers for file objects. It is advised to re-use such identifiers to avoid redundancy and to comply with the system/platform used to run the workflow.

workflow/

CWLProv ROs must include a system-independent executable version of the workflow under the `workflow/` folder. When using CWL, this sub-folder must contain the complete executable *workflow specification* file, an *input file object* with parameter settings used to enact the workflow and an *output file object* generated as a result of workflow enactment. The latter contain details of the workflow outputs such as data files produced by the workflow, but may exclude intermediate outputs.

To ensure RO portability, these file objects may not exactly match the file names at enactment time, as the absolute paths of the inputs are recommended to be replaced with relativized *content-addressed* paths within the RO, e.g. `/home/alice/exp15/sequence.fa` is replaced with `../data/b1/b1946ac92492d2347c6235b4d2611184`. The input file object should also capture any dependencies of the input data files, such as `.bam.bai` indexes neighbouring `.bam` (*Binary Alignment Map*) files. Any folder objects should be expanded to list contained files and their file names at time of enactment.

In the case of a CWL workflow, *cwltool* can aggregate the CWL description and any referenced external descriptions (such as sub-workflows or command line tool descriptions) into a single workflow file using `cwltool --pack`. This feature is used in our implementation (details in section **Practical Realisation of *CWLProv***) to rewrite the workflow files, making them re-executable without depending on workflow or commandline descriptions on the file system outside the RO. Other workflow definition approaches, WMS or CWL executors should apply similar features to ensure workflow definitions are executable outside their original file system location.

What is BagIt?

BagIt is an IETF Internet Standard (RFC8493)[14] that defines a structured file hierarchy for the purpose of digital preservation of data files. BagIt was initiated by the US Library of Congress and the California Digital Library, and is now used by libraries and archives to ensure safe transmission and storage of datasets using "bags".

A **bag** is indicated by the presence of `bagit.txt` and a *payload* of digital content stored as files and sub-folders in the `data/` folder. Other files are considered *tag files* to further describe the payload. All the payload files are listed in a *manifest* with checksums of their byte content, e.g. `manifest-sha256.txt` and equivalent for tag files in `tagmanifest-sha256.txt`. Basic metadata can be provided in `bag-info.txt` as key-value pairs.

A bag can be checked to be *complete* if all the files listed in the manifests exist, and is also considered *valid* if the manifest matches the checksum of each file, ensuring they have been correctly transferred.

BDBag (Big Data bag)[91] is a profile of BagIt that adds a *Research Object*[98] `metadata/manifest.json` in JSON-LD [99] format to contain richer Linked Data annotations that may not fit well in `bag-info.txt`, e.g. authors of an individual file. BDBags can include a `fetch.txt` to reference external resources using *ARK MinIDs* or HTTP URLs, allowing bags that contain large files without necessarily transferring their bytes.

snapshot/

`snapshot/` comprises copies of the workflow and tool specifications files "as-is" at enactment time, without any rewrites, packing or relativizing as described above.

It is recommended to use snapshot resources only for validity checking results and for understanding the workflow enactment, since these files might contain absolute paths or be host-specific, and thus may not be possible to re-enact elsewhere. Preserving these files untouched may nevertheless retain information that could otherwise get lost, e.g. commented out workflow code, or identifiers baked into file names.

A challenge in capturing snapshot files is that they typically live within a file system hierarchy which can difficult to replicate accurately, and may have internal references to other files. In our implementation we utilize `cwltool --print-deps` to find indirectly referenced files and store their snapshots in a flat folder.

metadata/

Each *CWLProv* RO must contain an RO manifest file `metadata/manifest.json` and two sub-directories `metadata/logs` and `metadata/provenance`. The RO manifest, part of the BDBag [91] profile, follows the JSON-LD structure defined for Research Object Bundles [98] and can provide structured Linked Data for each file in the RO, like file type and creation date. Further detail about the manifest file contents is documented on GitHub as *CWLProv* specification [84].

Any raw log information from the workflow enactment should be made available in `metadata/logs`. This typically includes the actual commands executed for each step. Similar to the snapshot files, log files may however be difficult to process outside the original enactment system. An example of such processing is *CWL-metrics* [100], which post-process cwltool log files to capture runtime metrics of individual Docker containers.

Capturing the details of a workflow execution require rich metadata in provenance files (see section **Retrospective Provenance Profile**). These should exist in the sub-folder `metadata/provenance`. It is recommended to make the availability of a *primary* provenance file mandatory, which should conform with the PROV-N [93] format. This file describes the top-level workflow execution. As described in *Level 2* (Section **Levels of Provenance and Resource Sharing**), it is quite possible to have nested workflows. In that case, a separate provenance file for each nested workflow execution should be included in this folder. If there are additional formats of provenance files such as PROV-JSON [94], PROV-XML [95], PROV-O [96] etc, then these should be included in the said folder with a declaration using `conformsTo` to declare their formats in the RO manifest being mandatory. The nested workflow profile should be named such that there is a link between the respective step in the primary workflow and the nested workflow preferably using unique identifiers.

As the PROV-DM has a generalized structure, there might be some provenance aspects specific to particular workflows that are hard to capture if only using PROV-N, hence ontologies such as *wfdesc* [101] can be used to describe the abstract representation of the workflow and its steps. Use of *wfprov* [102] to capture some workflow provenance aspects is also encouraged. Alternative extensions such as ProvOne [103] can also be utilized if the WMS or workflow executor is using these extensions already.

CWLProv reuses Linked Data standards like JSON-LD [99], W3C PROV [19] and Research Object [16]. A challenge with Linked Data in distributed and desktop computing is how to make identifiers that are absolute URIs and hence globally unique. For example, for *CWLProv* a workflow may be executed by an engine that does not know where its workflow provenance will be stored, published or finally integrated. To this end *CWLProv* generators should use the proposed *arcp* [104] URI scheme to map local file paths within the RO BagIt folder structure to absolute URIs for use within the RO manifest and associated PROV traces. Consumers of *CWLProv* ROs that do not contain an arcp-based External-Identifier should generate a temporary arcp base to safely resolve any relative URI references not present in the *CWLProv* folder. Implementations processing a *CWLProv* RO may convert arcp URIs to local `file:///` or `http://` URIs depending on how and where the *CWLProv* RO was saved, e.g. using the "arcp.py" library [105].

Retrospective Provenance Profile

As stated earlier, the primary provenance file should conform to the PROV-N [93] serialisation of PROV data model, and may optionally use ontologies specific to the workflow execution. The key features used in the structure of the retrospective provenance profile for a CWL workflow enactment in *CWLProv* are listed in Table 2). These features are not tied to any platform or workflow definition approach and hence can be used to document retrospective provenance of any workflow irrespective of the workflow definition approach.

The core mapping is following the PROV data model as in Figure 4): The PROV *Activity* represent the duration of a workflow run, as well as individual step executions, which *used* file and data (*Entity*), which again may be *wasGeneratedBy* previous step activities. The workflow engine (e.g. cwltool) is the *Agent* controlling these activities according to the workflow definition (*Plan*).

PROV is a general standard not specific to workflows, and lacks features to relate a *plan* (i.e. a workflow description) with sub-plans and workflow-centric retrospective provenance elements e.g. specific workflow enactment and its related steps enactment. We have utilized *wfdesc* and *wfprov* to represent few elements of prospective and retrospective provenance respectively. In addition, the provenance profile documented details

Table 2. Fulfilling recommendations with the *CWLProv* profile of W3C PROV, extended with Research Object Model's *wfdesc* (prospective provenance) and *wfprov* (retrospective provenance).

PROV type	Subtype	Relation	Range	Recommendation
**Plan**	wfdesc:Workflow	wfdesc:hasSubProcess	wfdesc:Process	R9-workflow
	wfdesc:Process			
**Activity**	wfprov:WorkflowRun	wasAssociatedWith	wfprov:WorkflowEngine	R8-environment
		↳ hadPlan	wfdesc:Workflow	R9-workflow, R17-executable
		wasStartedBy	wfprov:WorkflowEngine	R8-environment
		↳ atTime	*ISO8601 timestamp*	R13-provenance
		wasStartedBy	wfprov:WorkflowRun	R9-workflow
		wasEndedBy	wfprov:WorkflowEngine	R8-environment
		↳ atTime	*ISO8601 timestamp*	R13-provenance
	wfprov:ProcessRun	wasStartedBy	wfprov:WorkflowRun	R10-software
		↳ atTime	*ISO8601 timestamp*	R14-provenance
		used	wfprov:Artifact	R11-raw-data
		↳ role	wfdesc:InputParameter	R1-parameters
		wasAssociatedWith	wfprov:WorkflowRun	R9-workflow
		↳ hadPlan	wfdesc:Process	R17-executable, R16-format
		wasEndedBy	wfprov:WorkflowRun	R13-provenance
		↳ atTime	*ISO8601 timestamp*	R13-provenance
	SoftwareAgent	wasAssociatedWith	wfprov:ProcessRun	R8-environment
		↳ cwlprov:image	*docker image id*	R4-sw-version
**SoftwareAgent**	wfprov:WorkFlowEngine	wasStartedBy	Person *ORCID*	R12-attribution
		label	*cwltool --version*	R4-sw-version
**Entity**	wfprov:Artefact	wasGeneratedBy	wfprov:Processrun	R3-intermediate, R7-identifier
		↳ role	wfdesc:OutputParameter	R1-parameters
**Collection**	wfprov:Artefact	hadMember	wfprov:Artefact	R3-intermediate
	Dictionary	hadDictionaryMember	wfprov:Artefact	
		↳ pairKey	*filename*	R7-identifier

Indentation with ↳ indicates n-ary relationships which are expressed differently depending on PROV syntax. Namespaces: http://www.w3.org/ns/prov# (default), http://purl.org/wf4ever/wfdesc# (*wfdesc*), http://purl.org/wf4ever/wfprov# (*wfprov*), https://w3id.org/cwl/prov# *cwlprov*)

of all the uniquely identified *activities* e.g. workflow enactment and related command line tool invocations, their associated *entities* (e.g. input and output data artefacts, input configuration files, workflows and command line tool specifications). The profile also documents the relationship between activities such as which activity (workflow enactment) was responsible for starting and ending another activity (command line tool invocation).

As described in Section **Levels of Provenance and Resource Sharing**, in order to achieve maximum *white-box* provenance, the inner workings of a nested workflow should also be included in the provenance trace. If a step represents a nested workflow, a separate provenance profile is included in the RO. Moreover, in the parent workflow trace, this relationship is recorded using *has_provenance* as an attribute of the *Activity* step which refers to the profile of the nested workflow.

Practical Realisation of *CWLProv*

CWLProv [84] provides a format that can be adopted by any workflow executor or platform, provided that the underlying workflow definition approach is at least as declarative as CWL, i.e. it captures the necessary components described in Section **Applied Standards and Vocabularies**. In the case of CWL, as long as the conceptual constructs are common amongst the available implementations and executors, a workflow enactment can be represented in *CWLProv* format. To demonstrate the practical realisation of the proposed model we consider a Python-based reference implementation of CWL *cwltool*.

cwltool is a feature complete reference implementation of CWL. It provides extensive validation of CWL files as well as offering a comprehensive set of test cases to validate new modules introduced as extensions to the existing implementation. Thus it provides the ideal choice for implementing *CWLProv* for provenance support and resource aggregation. The existing classes and methods of the implementation were utilized to achieve various tasks such as packaging of the workflow and all associated tool specifications together. In addition, the existing python library *prov* [106] was used to create a provenance document instance and populate it with the required artefacts generated as the workflow enactment proceeds.

It should be noted that we elected to implement *CWLProv* in the reference implementation *cwltool* instead of the more scalable and production-friendly CWL implementations like Toil [107], Arvados [108], Rabix [86], CWL-Airflow [109] or Cromwell [87]. An updated list of implementations is available at the CWL homepage [2]. Compared to *cwltool* these generally have extensive scheduler and cloud compute support, and extensions for large data transfer and storage, and should therefore be considered for any adopters of the Common Workflow Language. In this study we have however focused on *cwltool* as its code base was found to be easy to adapt for rich provenance capture without having to modify subsystems for distributed execution or data management, and as a reference implementation better informing us on how to model *CWLProv* for the general case rather than being tied into execution details of the more sophisticated CWL workflow engines.

CWLProv support for *cwltool* is built as an optional module which when invoked as *"cwltool --provenance ro/ workflow.cwl job.json"*, will automatically generate an RO with the given folder name *ro/* without requiring any additional information from the user. Each input file is assigned a hash value and placed in the folder *ro/data*, making it content-addressable to avoid local dependencies (Figure 6).

In order to avoid including information about attribution without consent of the user, we introduce an additional flag

2 https://www.commonwl.org/#Implementations

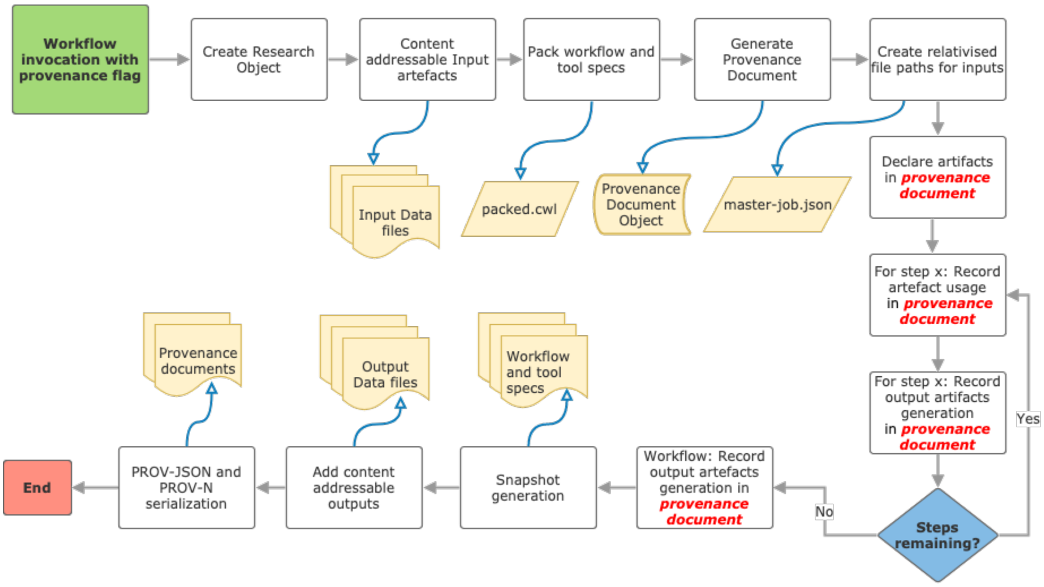

Figure 6. High level process flow representation of retrospective provenance capture

" *--enable-user-provenance*". If a user provides the options *--orcid* and *--full-name*, this information will be included in the provenance profile related to user attribution. Enabling " *--enable-user-provenance*" and not providing the full name or ORCID will store user account details from the local machine for attribution, i.e. the details of the *agent* that enacted the workflow.

The workflow and command line tool specifications are aggregated in one file to create an executable workflow and placed in folder *ro/workflow*. This folder also contains transformed input job objects containing the input parameters with references to artefacts in the *ro/data* based on relativising the paths present in the input object. These two files are sufficient to re-enact the workflow, provided the other required artefacts are also included in the RO and comply to the *CWLProv* format. The *cwltool* control flow [110] indicates the points when the execution of the workflow and command line tools involved in the workflow enactment start, end and how the output is reported back. This information and the artefacts are captured and stored in the RO.

When the execution of a workflow begins, *CWLProv* extensions to *cwltool* generate a provenance document (using the *prov* library) which includes default namespaces for the workflow enactment *"activity"*. The attribution details as an *agent* are also added at this stage if user provenance capture is enabled, e.g. to answer "who ran the workflow?". Each step of the workflow can correspond to either a command line tool or another nested workflow referred to as a *sub-workflow* in the CWL documentation. For each nested workflow, a separate provenance profile is initialized recursively to achieve a *white-box* finer-grained provenance view as explained in Section **Levels of Provenance and Resource Sharing**. This profile is continually updated throughout the nested workflow enactment. Each step is identified by a unique identifier and recorded as an *activity* in the parent workflow provenance profile, i.e. the *"primary profile"*. The *nested* workflow is recorded as a step in the *primary profile* using the same identifier as the "nested workflow enactment activity" identifier in the respective provenance profile. For each step in the activity, the start time and association with the workflow activity is created and stored as part of the overall provenance to answer the question "when did it happen?".

The data used as input by these steps is either provided by the user or produced as an intermediate result from the previous steps. In both cases, the *Usage* is recorded in the respective provenance profile using checksums as identifiers to answer the question "what was used?". The non-file input parameters such as strings and integers are stored "as-is" using an additional optional argument, *prov:value*. Upon completion, each step typically generates some data. The provenance profile records the generation of outputs at the step level to record "what was produced?" and "which process produced it?". Once all steps complete, the workflow outputs are collected and the generation of these outputs at the workflow level are recorded in the provenance profile. Moreover, using the checksum of these files generated by the *cwltool*, content-addressable copies are saved in the folder *ro/data*. The provenance profile refers to these files using the same checksum such that they are traceable or can be used for further analysis if required. The workflow specification, command line tool specifications and JSON job file is archived in the *ro/snapshot* folder to preserve the actual workflow history.

This prototype implementation provides a model and guidance for workflow platforms and executors to identify their respective features that can be utilized in devising their own implementation of *CWLProv*.

Achieving recommendations with provenance levels

Table 3 map the best practices and recommendations from Table 1 to the Levels of Provenance (Figure 2). The shown methods and implementation readiness indicate to which extent the recommendations are addressed by the implementation of *CWLProv* (detailed in this section).

Note that other approaches may solve this mapping differently. For instance, Nextflow [111] may fulfill *R18-resource-use* at Provenance Level 2 as it can produce trace reports with hardware resource usage per task execution [112], but not for the overall workflow. While a Nextflow trace report is a separate CSV file with implementation-specific columns, our planned *R18-resource-use* approach for CWL is to combine *CWL-metrics* [113], permalinks and the standard *GFD.204* [114] to further relate resource use with Level 1 and Level 2 provenance within the *CWLProv* Research Object.

In addition to following the recommendations from Table 1 through computational methods, the workflow authors are

Table 3. Recommendations and provenance levels implemented in *CWLProv*

Recommendation	L0	L1	L2	L3	Methods
R1-parameters	•		•		CWL, BP
R2-automate	•				CWL, Docker
R3-intermediate		•			PROV, RO
R4-sw-version	•		•		CWL, Docker, PROV
R5-data-version	•			•	CWL, BP
R6-annotation		•		⁎	CWL, RO, BP
R7-described		•			CWL, RO
R7-identifier		•	•	•	RO, CWLProv
R8-environment		⁎	⁎		GFD.204
R9-workflow	•	•	•		CWL, wfdesc
R10-software	•		•		CWL, Docker
R11-raw-data	•	•			CWLProv, BP
R12-attribution		•			RO, CWL, BP
R13-provenance		•	•		PROV, RO
R14-diagram	○			⁎	CWL, RO
R15-open-source	•				CWL, BP
R16-format		•		•	CWL, BP
R17-executable	○	•			CWL, Docker
R18-resource-use		⁎	⁎		CWL, GFD.204
R19-example	⁎	○			RO, BP

CWL: Common Workflow Language and embedded annotations
RO: Research Object model and BagIt
PROV: W3C Provenance model
CWLProv: Additional attributes in PROV
wfdesc: Prospective provenance in PROV
BP: Best Practice need to be followed manually
• Implemented
○ Partially implemented
⁎ Implementation planned/ongoing

also required to exercise *best practices for workflow design and authoring*. For instance, to achieve *R1-parameters* the workflow must be written in such a way that parameters are exposed and documented at workflow level, rather than hard-coded within an underlying Python script. Similarly, while the CWL format support rich details of user annotations that can fulfill *R6-annotation*, for these to survive into a Research Object at execution time, such annotation capabilities must actually be used by workflow authors instead of unstructured text files.

It should be a goal of a scientific WMS to guide users towards achieving the required level of the provenance framework through automation where possible. For instance a user may in the workflow have specified a Docker container image without preserving the version, but the provenance log could still record the specific container version used at execution time, achieving *R4-sw-version* retrospectively by computation rather than relying on a prospective declaration in the workflow definition.

CWLProv Evaluation with Bioinformatics Workflows

CWLProv as a standard supports *syntactic*, *semantic* and *pragmatic* interoperability (defined in Section **Interoperability**) of a given workflow and its associated results. We have defined a *"common data format"* for workflow sharing and publication such that any executor or WMS with CWL support can interpret this information and make use of it. This ensures the *syntactic* interoperability between the workflow executors on different computing platforms. Similarly the *"content"* of the shared aggregation artefact as a workflow-centric RO is unambiguously defined, thus ensuring uniform representation of the workflow and its associated results across different platforms and executors hence supporting *semantic* interoperability. With *Level 3* provenance satisfied providing domain-specific information along with level 0-2 provenance tracking, we posit that *CWLProv* would be able to accomplish *pragmatic* interoperability by providing unambiguous information about the *"context"*, *"application"* and *"use"* of the shared/published workflow-centric ROs. Hence, extension of the current implementation (described in section) in future to include domain-rich information in the provenance traces and the *CWLProv RO* will result in pragmatic interoperability.

To demonstrate the interoperability and portability of the proposed solution, we evaluate *CWLProv* and its reference implementation using open source bioinformatics workflows available on GitHub from different research initiatives and from different developers. Conceptually, these workflows are selected for evaluation due to their excessive use in real-life data analyses and variety of the input data. Alignment workflow is included in the evaluation as it is one of the most time consuming yet mandatory steps in any variant calling workflow. Practically, choosing the workflows by these particular groups out of numerous existing implementations is justified in each section below.

RNA-seq Analysis Workflow

RNA sequencing (RNA-seq) data generated by Next Generation Sequencing (NGS) platforms is comprised of short sequence reads that can be aligned to a reference genome, where the alignment results form the basis of various analyses such as quantitating transcript expression; identifying novel splice junctions and isoforms and differential gene expression [116]. RNA-seq experiments can link phenotype to gene expression and are widely applied in multi-centric cancer studies [24]. Computational analysis of RNA-seq data is performed by different techniques depending on the research goals and the organism under study [117]. The workflow [118] included in this case study has been defined in CWL by one of the teams [119] participating in NIH Data Commons initiative [120], a large research infrastructure program aiming to make digital objects (such as data generated during biomedical research and software/tools required to utilize such data) shareable and accessible and hence aligned with the FAIR principles [67].

This workflow (Figure 7), designed for the pilot phase of the NIH Data Commons initiative [121], adapts the approach and parameter settings of Trans-Omics for precision Medicine (TOPMed) [122]. The RNA-seq pipeline originated from the Broad Institute [123]. There are in total five steps in the workflow starting from: 1) Read alignment using STAR [124] which produces aligned BAM files including the Genome BAM and Transcriptome BAM. 2) The Genome BAM file is processed using Picard MarkDuplicates [125] producing an updated BAM file containing information on duplicate reads (such reads can indicate biased interpretation). 3) SAMtools index [126] is then employed to generate an index for the BAM file, in preparation for the next step. 4) The indexed BAM file is processed further with RNA-SeQC [127] which takes the BAM file, human genome reference sequence and Gene Transfer Format (GTF) file as inputs to generate transcriptome-level expression quantifications and standard quality control metrics. 5) In parallel with transcript quantification, isoform expression levels are quantified by RSEM [128]. This step depends only on the output of the STAR tool, and additional RSEM reference sequences.

For testing and analysis, the workflow author provided example data created by down-sampling the read files of a TOPMed public access data [129]. Chromosome 12 was extracted from the *Homo Sapien Assembly 38* reference sequence and provided by the workflow authors. The required GTF and

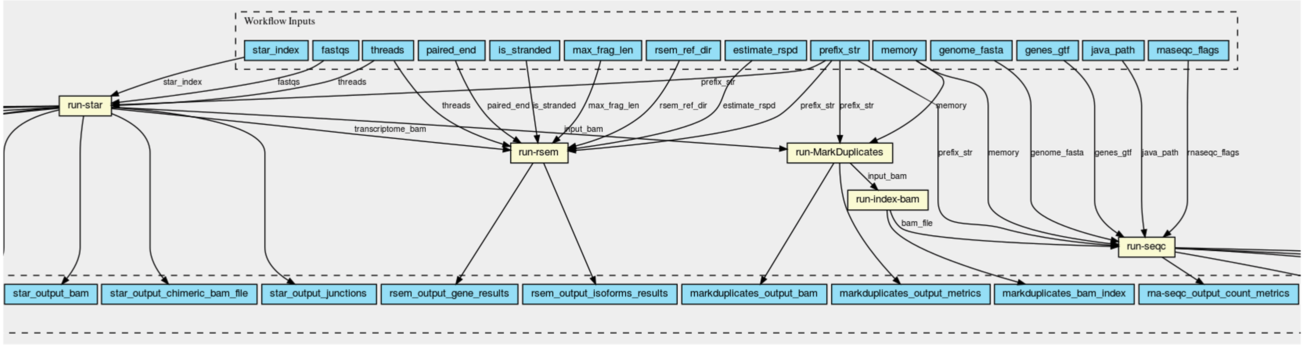

Figure 7. Portion of a RNA-seq workflow generated by CWL viewer [115].

RSEM reference data files are also provided. The workflow is well-documented with a detailed set of instructions of the steps performed to down-sample the data are also provided for transparency. The availability of example input data, use of containerization for underlying software and detailed documentation are important factors in choosing this specific CWL workflow for *CWLProv* evaluation.

Alignment Workflow

Alignment is an essential step in variant discovery workflows and considered an obligatory *pre-processing* stage according to Best Practices by the Broad Institute [72]. The purpose of this stage is to filter low-quality reads before variant calling or other interpretative steps [130]. The workflow for alignment is designed to operate on raw sequence data to produce analysis-ready BAM files as the final output. The typical steps followed include file format conversions, aligning the read files to the reference genome sequence, and sorting the resulting files. The CWL alignment workflow [131] included in this evaluation (Figure 8) is designed by Data Biosphere [132]. It adapts the alignment pipeline [133] originally developed at Abecasis Lab, The University of Michigan [134]. This workflow is also part of NIH Data Commons initiative (as RNA-seq Analysis Workflow) and comprises of four stages. First step, "Pre-align" accepts a Compressed Alignment Map (CRAM) file (a compressed format for BAM files developed by European Bioinformatics Institute (EBI) [135]) and human genome reference sequence as input and using underlying software utilities of SAMtools such as view, sort and fixmate returns a list of fastq files which can be used as input for the next step. The next step "Align" also accepts the human reference genome as input along with the output files from "Pre-align" and uses BWA-mem [136] to generate aligned reads as BAM files. SAMBLASTER [137] is used to mark duplicate reads and SAMtools view to convert read files from SAM to BAM format. The BAM files generated after "Align" are sorted with "SAMtool sort". Finally these sorted alignment files are merged to produce single sorted BAM file using SAMtools merge in "Post-align" step. The authors provide an example CRAM file, *Homo Sapien Assembly 38* reference genome along with its index files to be used as inputs for testing and analysis of the workflow.

Somatic Variant Calling Workflow

Variant discovery analysis for high-throughput sequencing data is a widely used bioinformatics technique, focused on finding genetic associations with diseases, identifying somatic mutations in cancer and characterizing heterogeneous cell populations [138]. The *pre-processing* explained for the Alignment workflow is part of any variant calling workflow as reads are classified and ordered as part of the variant discovery process. Numerous variant calling algorithms have been developed depending on the input data characteristics and the specific application area [130]. Somatic variant calling workflows are designed to identify somatic (non-inherited) variants in a sample – generally a cancer sample – by comparing the set of variants present in a sequenced tumour genome to a non-tumour genome from the same host [139]. The set of tumour variants is a super-set of the set of host variants, and somatic mutations can be identified through various algorithmic approaches to subtracting host familial variants. Each somatic variant calling workflow typically consists of three stages: pre-processing; variant evaluation and post-filtering.

The somatic variant calling workflow (Figure 9) included in this case study is designed by Blue Collar Bioinformatics (bcbio) [140], a community-driven initiative to develop best-practice pipelines for variant calling, RNA-seq and small RNA analysis

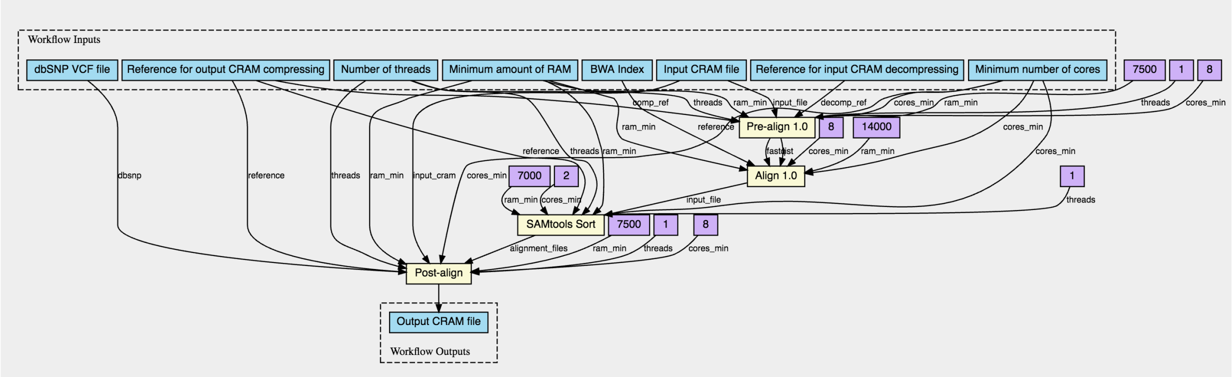

Figure 8. Alignment workflow representation generated by CWL viewer.

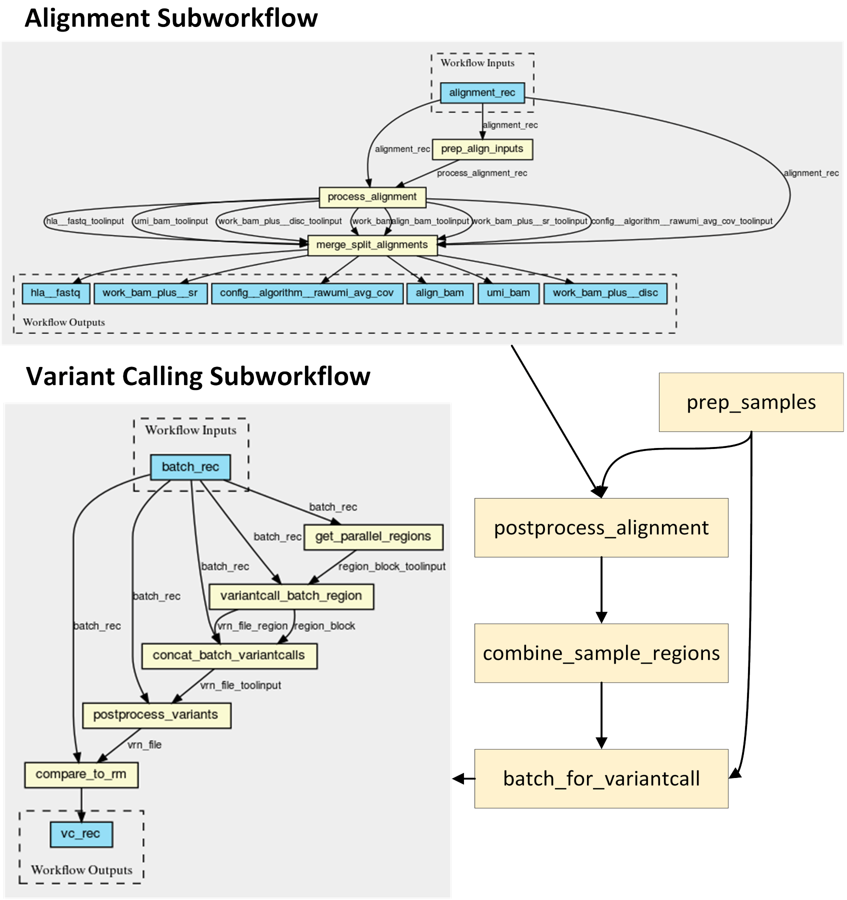

Figure 9. Visual representation of the bcbio somatic variant calling workflow (Adapted from [142]) and the subworkflow images are generated by CWL viewer.

workflows. According to the documentation, the goal of this project is to facilitate the automated analysis of high throughput data by making the resources *quantifiable*, *analyzable*, *scalable*, *accessible* and *reproducible*. All the underlying tools are containerized facilitating software use in the workflow. The somatic variant calling workflow defined in CWL is available on GitHub [141] and equipped with a well defined test dataset.

Evaluation Activity

This section describes the evaluation of cross-executor and cross-platform interoperability of *CWLProv*. To test cross-executor interoperability, two CWL executors *cwltool* and *toil-cwl-runner* were selected. *toil-cwl-runner* is an open source Python workflow engine supporting robust cross-platform workflow execution on Cloud and High Performance Computing (HPC) environments [107]. The two operating system platforms utilized in this analysis were MacOS and Ubuntu Linux. For the Linux OS, a 16-core Linux instance with 64GB RAM was launched on the Australian National eResearch Collaboration Tools and Resources (NeCTAR) research cloud [143]. To cater for the storage requirements, a 1000GB persistent volume was attached to this instance. For MacOS, a local system with 16GB RAM, 250GB storage and 2.8 GHz Intel Core i7 processor was used. These platforms were selected to cater for the required storage and compute resources of the workflows described above. The reference genome provided with Alignment Workflow was not down-sampled and hence this workflow required most resources among the three evaluated.

It is worth mentioning that this evaluation does not include details of the installation process for *cwltool*, *toil-cwl-runner* and *Docker* on systems described above. To create *CWLProv* ROs during workflow execution, it is necessary to use the CWL reference runner (*cwltool*) until this practice spreads to other CWL implementations. Moreover, it is assumed that the software container (Docker) should also be installed on the system to use the workflow definitions aggregated in a given *CWLProv* RO.

In addition, the resource requirements (identified in *R18-resource-use* and discussed in Section **Discussion and Future Directions**) should also be satisfied by choosing a system with enough compute and storage resources for successful enactment. The systems used in this case study should be a reference when selecting a system as inadequate compute and storage resources such as insufficient RAM or number of cores will hinder the successful re-enactment of workflows using these ROs. The hardware requirements may also vary if a different dataset is used as input to re-enact the workflow using the methods aggregated in the RO. In that case, the end user must ensure availability of adequate compute and storage resources by choosing a system that meets the required specifications [144].

Since the *CWLProv* implementation is demonstrated for one of the executors (*cwltool*), currently a *CWLProv* RO for any workflow can only be produced using *cwltool*. Hence, in this activity the workflows are initially enacted using just *cwltool* (Table 4). The outline of the steps performed to analyse *CWLProv* for each case study is as follows.

I) The workflow was enacted using *cwltool* to produce a RO on a MacOS computer.

1) The resulting RO and aggregated resources were used

to re-enact the workflow using *toil-cwl-runner* on the same MacOS computer;
2) The RO produced in step I was transferred to the cloud-based Linux instance used in this activity;
3) On the cloud-based Linux environment and only utilizing the resources aggregated in the RO, the workflow was re-enacted using *cwltool* and *toil-cwl-runner*.

II) The workflow was enacted using *cwltool* to produce a RO on Linux.

1) The resulting RO and aggregated resource were utilized to re-enact the workflow using *toil-cwl-runner* on the same cloud-based Linux instance;
2) The RO produced in step II was transferred to the MacOS computer used in this activity;
3) On the MacOS computer and only utilizing the resources aggregated in the RO, the workflow was re-enacted using *cwltool* and *toil-cwl-runner*.

The *CWLProv* ROs produced as a results of this activity are published on Mendeley Data [145, 146, 147] with mirrors on Zenodo.

Evaluation Results

The steps described above were taken to produce ROs which were then used to re-enact the workflows (outlined in Table 4), without any further changes required. This demonstration illustrated the syntactic and semantic interoperability of the workflows across different systems. It shows that **both CWL executors were able to *exchange*, *comprehend* and *use* the information represented as *CWLProv* ROs**. The current implementation described in section **Practical Realisation of *CWLProv*** does not resolve *Level 3*. Hence, the inclusion of domain-specific annotations referring to scientific context to address pragmatic interoperability is identified as crucial future direction and further detailed in section **Discussion and Future Directions**.

Table 4. *CWLProv* evaluation summary and status for the 3 bioinformatics case studies.

Enact-produce RO with	Re-enact using RO with	Status
cwltool on MacOS	*toil-cwl-runner* on MacOS	✓
	cwltool on Linux	✓
	toil-cwl-runner on Linux	✓
cwltool on Linux	*toil-cwl-runner* on Linux	✓
	cwltool on MacOS	✓
	toil-cwl-runner on MacOS	✓

CWLProv and Interoperability

CWL already builds on technologies such as JavaScript Object Notation for Linked Data (JSON-LD) [99] for data modeling and Docker [30] to support portability of the run-time environments. The portability and interoperability as basic principles of the underlying workflow definition approach for any workflow-centric analysis implies that the analysis should also be portable and interoperable. However, the workflow definition/specification alone is insufficient when dealing with commandline tool specifications, data, and input configuration files used in the analysis if these are not readily available.

CWLProv ensures availability of these resources for a given analysis conforming to the framework defined in Section **CWL-Prov 0.6.0 and utilized standards**. The input configurations are saved as *primary-job.json* in folder *workflow/* and refer to the input data contained in the payload *data/* folder of the given RO. In this way, availability of data aggregated with the analysis is made possible. Existing features of *cwltool* are used to generate the CWL workflow specification file containing all of the commandline tool specifications referred to in the workflow specification and placed in the same *workflow/* folder.

One might argue that copying a folder tree might serve the same purpose but in that case we again will be relying on users to put substantial amount of effort on top of the actual analysis, i.e. they would have to carefully structure their directories to be aligned with the workflow creators. Instead CWL encourages researchers to utilize container technologies such as Docker, Singularity, or software packaging systems like Debian (Med) or Bioconda to ensure availability of underlying tools as recommended by numerous studies [13, 6, 57, 63, 64, 148]. This practice facilitates the preservation of methods utilized in data-intensive scientific workflows and enables verification of the published claims without requiring the end-user to do any manual installation and configuration. Examples of tools available via Docker containers used here are the alignment tool (BWA mem) used in the Alignment workflow and STAR aligner used in RNA-seq workflow.

Evaluating Provenance Profile

The retrospective provenance profile generated as part of *CWL-Prov* for each workflow enactment can be examined and queried to extract the required subset of information. *Provenance Analytics* is a separate domain and a next step after provenance collection in the provenance life cycle [149]. Often provenance data is queried using specialized query languages such as SQL SPARQL or TriQL depending on the storage mechanism used. Query operations can combine information from prospective and retrospective provenance to understand computational experiments better.

The focus of this paper is not in-depth provenance analytics but we have demonstrated the application of the provenance profile generated as part of *CWLProv*. We have developed a commandline tool and Python API *"cwlprov-py"* [150] for *CWLProv* RO analytics to interpret the captured retrospective provenance of CWL workflow enactment. This API currently supports the following use-cases.

Given a *CWLProv* RO:

- **Workflow Runs**
  As each RO can contain more than one *workflow run* if sub-workflows are utilized to group related tasks into one workflow. In that case, the provenance traces are stored in separate files for each workflow run. *cwlprov-py* identifies the workflow enactments including the sub-workflows *(if any)* and returns the workflow identifiers annotated with the step names. The user can select the required trace and explore particular traces in detail.
- **Attribution**
  Each RO is assumed to be associated with a single enactment of the primary workflow and hence assumed to be enacted by one person. As discussed previously, *CWLProv* provides additional flags to enable user provenance capture. A user can provide their name and ORCID details that can be stored as part of a RO. *cwlprov-py* displays attribution details of the researcher responsible for the enactment *(if enabled)* and the versions of the workflow executor utilized in the analysis.
- **Input/Output of a Process**
  Provenance traces contain associations between the steps/workflows with the data they used or generated. A user interested in a particular step can identify the inputs used and outputs produced linked explicitly to that process

Table 5. Run-time comparison for the workflow enactments done cross-executor and cross-platform

Workflow	Linux			MacOS		
	cwltool		toil-cwl-runner	cwltool		toil-cwl-runner
	With Prov	W/O Prov	W/O Prov	With Prov	W/O Prov	W/O Prov
RNA-Seq Analysis Workflow	4m30.289s	4m0.139s	3m46.817s	3m33.306s	3m41.166s	3m30.406s
Alignment Workflow	28m23.792s	24m12.404s	15m3.539s	–	162m35.111s	146m27.592s
Somatic Variant Calling Workflow	21m25.868s	19m27.519s	7m10.470s	17m26.722s	17m0.227s	**

** This could not be tested because of a Docker mount issue on MacOS: https://github.com/DataBiosphere/toil/issues/2680
– This could not be tested because of the insufficient hardware resources on the MacOS test machine, hence step I of the evaluation activity could not be performed for this workflow

using *cwlprov-py*. This option works using individual step identifiers (level 1) as well as nested workflows (level 2), facilitating re-use of intermediate data even if the original workflow author did not explicitly expose these as workflow outputs.

- **Partial Re-runs**
  Re-running or re-using only desired parts of a given workflow has been emphasized [24] as important to evaluate the workflow process or validate the published results associated without necessarily re-enacting the workflow as a whole. *cwlprov-py* uses the identifier of the step/workflow to be re-run, parses the provenance trace to identify the inputs required and ultimately creates a JSON input object with the associated input parameters. This input object can then be used for partial re-runs of the desired step/workflow, making segmented analysis possible even for CWLProv consumers who don't have sufficient hardware resources for re-executing more computationally heavy steps.

While the above explores some use cases for consuming and re-using workflow execution data, we have not explored this in full detail. Further work could develop more specific user scenarios and perform usability testing with independent domain-experts who have not seen the executed workflow before.

An important point of *CWLProv* is to capture sufficient information at workflow execution time, so that post-processing (potentially by a third-party) can support unforeseen queries without requiring instrumentation at workflow design time. For instance, `cwlprov runtimes` calculates average runtime per step (requiring capture of start/stop time of each step iteration), while `cwlprov derived` calculates derivation paths back to input data (requiring consistent identifiers during execution). Further work could build a more researcher-oriented interface based on this approach, e.g. hardcoded data exploration for a particular workflow.

Temporal and Spatial Overhead with Provenance

Table 5 shows the run-times for the three workflow enactments using cwltool and toil-cwl-runner on Linux and MacOS with and without enabling provenance capture as described in the evaluation activity section. These workflows were enacted at least once before this time calculation, hence the timing does not include the time for Docker images to be downloaded. On a new system, when re-running these workflows for the first time, the Docker images will be downloaded and may take significantly longer than the time specified here especially in case of the Somatic Variant Calling workflow because of the image size.

Run-time and storage overheads are important for provenance-enabled computational experiments. The choice of different operating systems and provenance capture mechanisms such as operating-system level, application-level or workflow-level as well as I/O workload, interception mechanism and fine-grained information capture are key for provenance [151, 152].

In our case study, significant time difference can be seen for the alignment workflow that used the most voluminous dataset, hence producing a sizable RO as well. This was due to the RO-generation where data was aggregated within the RO. The difference between the provenance-enabled enactment versus the enactment without provenance is barely noticeable for the other two workflow enactments with the smaller datasets. The discussion about handling the big '-omics' data such as human genome reference sequence, its index files and other database files (e.g. dbsnp) in Section **Discussion and Future Directions** provides a possible solution to avoid such overheads.

In addition, noticeable time difference between the cwltool and toil-cwl-runner enactments is because of the default parallel versus serial job execution in case of *toil-cwl-runner* and *cwltool* respectively. The "scatter" operation in CWL when applied to one or more input parameters of a workflow step or a sub-workflow, supports parallel execution of the associated processes. Parallelism is also available without "scatter" when separate processes have all their inputs ready. If sufficient compute resources are available, these jobs will be enacted concurrently otherwise they are queued for subsequent execution. Compute intensive steps of a workflow can benefit from scatter features for parallel execution by reducing the overall run-time. Both Alignment and Somatic Variant Calling workflows utilize the scatter feature to enable higher degrees of parallel job execution in case of *toil-cwl-runner* which explains the time difference for the cross-executor of these two workflows. The difference is negligible for RNA-Seq workflow which is comprised of serial jobs with comparatively small test data.

Output Comparison Across Enactments

We compared the workflow outputs after each enactment to observe the concordance and/or discordance (if any) for the workflow enactment results produced across the platforms and across the executors. As *CWLProv* RO refers to the data with hashed checksums, these checksums are utilized for the result comparison. It is worth-mentioning that the comparison was made between the output files generated by the different enactments against a single *"truth-set"* output file available and checksum in the respective Git repositories.

The checksum of the output data generated cross-platform and cross-executor comparison data as a result of the initial enactments and re-runs using the CWL ROs to elicit the concordance in all but one cases. The "correctness" as well as agreement of these outputs given different execution environments (e.g. platform and executor) hold true except for Alignment workflow. Alignment workflow produced varying outputs after every execution even with the same executor and platform. The output of the alignment algorithm, "BWA mem" used in this workflow was non-deterministic as it depended on the *number of threads --t* and the *seed length --K* which affected the output produced. While the seed length in this case was set to a constant value, the number of threads varied depending on the availability of hardware resources at run-time, thereby resulting in varying output for the same input files.

Discussion and Future Directions

This section discusses the current and future work with reference to enriched provenance capture and smart resource aggregation, and enhancements to both the *CWLProv* standard and implementation.

Compute and Storage Resources

The *CWLProv* format encapsulates the data and workflow definitions involved in a given workflow enactment along with its retrospective provenance trace. CWL as a standard provides constructs to declare basic hardware resource requirements such as minimum and maximum cores, RAM and reserved file system storage required for a particular workflow enactment. The workflow authors can provide this information in the *"requirements"* or *"hints"* section as *"ResourceRequirement"*. These requirements/hints can be declared at workflow or individual step level, to help platforms/executors to allocate the required resources. This information indirectly stores some aspects of prospective view of provenance with respect to hardware requirements of the underlying system used to enact a workflow. Currently this information is only available if declared as part of workflow specification. In future, we plan to include these requirements as part of provenance for a given workflow such that all such information is gathered in one space and users are not required to inspect multiple sources to extract this information. This information can then be used as a pre-condition for potential successful enactment of a given workflow.

As *CWLProv* is focused on retrospective provenance capture of workflow enactment, we plan to include provenance information about the compute and storage resources utilized in a given enactment to fulfill *R18-resource-use*. We believe that documenting these resources will allow users to analyse their environment and resource allocations before execution, as opposed to trial and error methods that may result in multiple failed enactments of a given workflow. Despite being an important factor, it is surprising to see that most of existing provenance standards lack dedicated constructs to represent the underlying hardware resource usage information as part of prospective or retrospective provenance. In the case of complex workflows using distributed resources, where each step could be executed on a different node/server, including all this information in a single *PROV* profile will clutter the profile and render it potentially incomprehensible. Therefore, we plan to add a separate *Usage Record* document in the RO conforming to GFD.204 [114] to describe *Level 1* (and potentially *Level 2*) resource usage in a common format independent on actual execution environment.

Capturing such resource usage records require a tighter integration with the execution platform, and so we consider this future work better suited for a cloud-based CWL engine like *Toil* or *Arvados*, as the reference implementation *cwltool* does not exercise fine-grained control of its task execution. Detailed raw log files can also be provided as *Level 0* provenance, as we have demonstrated with cwltool, but these will by their nature be custom per execution platform and thus should be considered unstructured. Related work that is already exploring this approach is *cwl-metrics* [113], which analyses raw *cwltool* log files in combination with detailed Docker invocation statistics using the container monitoring tool *Telegraf*. Ongoing collaboration is exploring adding these metrics as additional provenance to the *CWLProv* RO with summaries in PROV and GFD.204 formats.

Provenance Profile Augmented with Domain Knowledge

CWLProv benefits from existing best practices proposed by numerous studies (Table 1) and includes defined standards for workflow representation, resource aggregation and provenance tracking (Section **Applied Standards and Vocabularies**). We posit that the principle of following well-defined data and metadata standards enables explicit data sharing and reuse. In order to include rich metadata for bioinformaticians to produce specialized ROs for bioinformatics to achieve *CWLProv Level 3* as defined in section **Levels of Provenance and Resource Sharing**, we are investigating re-use of concepts from the BioCompute Object (BCO) project [9]. This domain-specific information is not necessary for computation and execution but for understandability of the shared resources. We encourage workflow authors to include such metadata and external identifiers for data and underlying tools, e.g. EDAM identifiers for the resources employed in designing a given workflow. The plan is to extract these annotations and represent in the retrospective provenance profile in *CWLProv* to ultimately achieve pragmatic interoperability by providing domain-specific scientific context of the experiments. Domain-specific information is essential in determining the nature of inputs, outputs and context of the processes linked to a given workflow enactment [74]. This information can be captured in the RO if and only if the workflow author adds it in the workflow definition, thus achieving *CWLProv Level 3* depends on the individual workflows.

Big -omics Data

While aggregating all resources as one download-able object improves reproducibility, the size of the resulting RO is an important factor in practice. On one hand, completeness of the resources contributes towards minimizing the *workflow decay* phenomenon by least dependence on availability of third party resources. On the other hand, the nature of -omics data sizes can result in hard-to-manage workflow-centric ROs also leading to the spatial and temporal overheads as discussed in evaluation.

One solution is archiving the big datasets in online repositories or data stores and including the existing persistent identifiers and checksums in the RO instead of the actual data files, as previously demonstrated with BDBags [91, 153]. While CWL executors like *toil-cwl-runner* can be configured to deposit data in a shared repository, the *cwltool* reference implementation explored in this study can only write to the local file system. External references raise the risk of unavailability of data at a later time. Therefore we recommend including the data in the RO if sufficient network and storage resources are available. Future work may explore post-processing *CWLProv* ROs to replace large data files with references to stable data repositories, producing a slimmer RO for transfer where individual data items can be retrieved on demand, as well as reducing data duplication across multiple related ROs.

Improving CWLProv efficiency with selective provenance capture

Shim refers to an adaptor step to resolve a format incompatibility issues between two workflow tasks [61], typically converting the previous output into an acceptable format for the next step. For example in our case study *RNA-seq* workflow, *RNA-SeQC* require an indexed BAM file, whereas the output of *STAR* or *Picard MarkDuplicates* only comprises of the BAM file alone. Hence, a shim step executing *SAMtools index* make the aligned reads analysis ready for RNA-SeQC. Compared to the more analytical steps, the provenance of such shim steps are not particularly interesting for domain scientists, and in many cases their intermediate data would effectively double the storage cost with little information gains, as such data can be reliably recreated by re-applying the predictable transformation step (considering it as a *pure function* without side-effects). Another type of ignorable steps could be purely diagnostic, which outputs are used primarily during workflow design to verify tool settings. A workflow engine does not necessarily know

which steps are "boring" [3] and our proof of concept implementation will dutifully store provenance from all steps.

To improve efficiency, future *CWLProv* work could add options to ignore capturing outputs of specified *shim* steps, or to not store files over a particular file size. Similarly a scientist or a WMS may elect to only capture provenance at a particular provenance level (see Section **Levels of Provenance and Resource Sharing**). Provenance captured under such settings would be "incomplete" (e.g. PROV would say *RNA-SeQC* consumed an identified BAM index file, but the corresponding bytes would not be stored in the RO), thus it is envisioned this can be indicated in the RO manifest as a variant of the *CWLProv* profile identifier to give the end-user clear indication of what to expect in terms of completeness, so that tools like *cwlprov-py* could be extended to re-create missing outputs, verifying their expected checksums, or collapse provenance listing of "boring" steps to improve human tractability.

Enforcement of Best Practices – An Open Problem

Recommendations and best practices from the scientific community are proposed frequently, to guide researchers to design their computational experiments in such a way as to make their research reproducible and verifiable. Not only the best practices for workflow design, but also for resource declaration, software packaging and configuration management are put forward [148] to avoid dependence on local installations and manual processes of dependency management. The term *"Better Software, Better Research"* [154] can also be well-applied on and adapted for the workflow design process.

Declarative approaches to workflow definition such as CWL facilitate and encourage users to explicitly declare everything in a workflow, improving white-box view of the retrospective as well as prospective provenance. Such workflows should provide insights of the complete process followed, to produce a data artefact resolving the black-boxness often associated with the workflow provenance. However, it is entirely up to researchers to leverage these approaches to produce well-defined workflows with explicit details facilitating enriched capture of the provenance trace at the appropriate level, and this can require considerable effort and consistency on the workflow designer's behalf. For instance, the alignment workflow used in this case study embeds bash scripts into the CWL tool definition, therefore requiring another layer needed to be penetrated for provenance information extraction. Despite using CWL for the workflow definition and *CWLProv* for provenance capture, the provenance trace will be missing critical information making it coarse-grained, and the raw logs capturing the enactment will also not be as informative.

The three criteria defined by Cohen-Boulakia et al. [24] to be followed by workflow designers are: modularized specifications, unified representation and workflow annotations. CWL facilitates a modular structure to workflow definitions by coupling similar steps to *subworkflows*; and, as an interoperable standard, CWL provides a common platform moving towards resolution of the heterogeneity of the workflow specification languages. In addition, users can add standardised domain-specific annotations to data and workflows incorporating the constructs defined by external ontologies (e.g. EDAM) to enhance understanding of the shared specification and the resources it refers to. All these features can be utilized to design better workflows and maximize the information declaration resulting in semantically-rich and provenance-complete *CWLProv* ROs, and should thus be expressed clearly in user guides[4] for workflow authors.

3 The CWL 1.1 specification will add a hint `WorkReuse` for this purpose.
4 See for instance https://view.commonwl.org/about#format

The usability of any *CWLProv* RO directly relies on the choice of practices followed by the researchers to design and communicate their computational analyses. Workflow-centric initiatives similar to *software carpentry* [155] and *code is science* [156] are one possible way to organize training and create awareness around best practices. Community-driven efforts to further consolidate the understanding of requirements to make a given workflow explicit and understandable should be made. Not only awareness about the workflow design is needed, but also the availability of the associated resources should be emphasized e.g. software as containers or software packages, big datasets in public repositories and pre-processing/post-processing as part of workflow. Without putting proposed best practices into actual practice, complete communication and hence the reproducibility of a workflow-centric computational analysis is likely to remain challenging.

Conclusion

The comprehensive sharing and communication of the computational experiments employed to achieve a scientific objective establishes trust on published results. Shared resources are sometimes rendered ineffective due to incomplete provenance, heterogeneity of platforms, unavailability of software and limited access to data. To this context, the contributions of this study are four-fold. First, we have provided a comprehensive summary of the recommendations put forward by the community regarding workflow design and resource sharing. Second, we define a hierarchical provenance framework to achieve homogeneity in the granularity of the information shared with each level addressing specific provenance recommendations.

Third, we leverage the existing standards best suited to define a standardized format, *CWLProv* for methodical representation of workflow enactments, its provenance and the associated artefacts employed. Finally, to demonstrate the applicability of *CWLProv*, we extend an existing workflow executor (*cwltool*) to provide a reference implementation to generate interoperable workflow-centric ROs, aggregating and preserving data and methods to support the coherent sharing of computational analyses and experiments.

With any published scientific research, statements such as *"Methods and data are available upon request"* should no longer be acceptable in a modern open-science-driven research community. Considering on one hand the collaborative nature and emerging openness of bioinformatics research and on the other hand the heterogeneity of workflow design approaches, it is essential to provide open access to the structured representation of the data and methods utilized in any scientific study to achieve interoperable solutions facilitating reproducibility of science.

Provenance capture and its subsequent use to support published research transparency should not be treated as an afterthought but rather as a standard practice of up-most priority. With adoption of well-defined standards for provenance and declarative workflow definition approaches, the assumption of black-box provenance often associated with workflows can be addressed. The workflow authors should be encouraged to follow well-established and agreed upon best practices for workflow design and software environment deployment. In conclusion, we do not require new standards, new WMSs or indeed new best practices, instead the focus should be to implement, utilize and re-use existing mature community-driven initiatives to achieve consensus in representing different aspects of computational experiments.

Availability of source code and requirements

CWLProv is implemented as part of the CWL reference implementation *cwltool*:

- Project name: cwltool (RRID:SCR_015528)
- Project home page: `https://github.com/common-workflow-language/cwltool`
- Version: 1.0.20181012180214 [10]
- Operating system(s): Platform independent
- Programming language: Python 3.5 or later (RRID:SCR_008394)
- Other requirements: Docker (RRID:SCR_016445) recommended
- License: Apache License, Version 2.0

The *CWLProv profile* documents the use of W3C PROV in a Research Object to capture a CWL workflow run:

- Project name: *CWLProv* profile
- Project home page: `https://w3id.org/cwl/prov`
- Version: 0.6.0 [84]
- Operating system(s): Platform independent
- License: Apache License, Version 2.0

The *CWLProv Python Tool* can be used to explore *CWLProv* ROs on the command line:

- Project name: *CWLProv* Python Tool (*cwlprov-py*)
- Project home page: `https://github.com/common-workflow-language/cwlprov-py`
- Version: 0.1.1 [150]
- Operating system(s): Platform independent
- Programming language: Python 3.5 or later (RRID:SCR_008394)
- License: Apache License, Version 2.0

Availability of supporting data and materials

CWLProv Research Objects of CWL workflow executions are published in Mendeley Data and mirrored to Zenodo.

- CWL run of Somatic Variant Calling Workflow (CWLProv 0.5.0 Research Object) [147]
  `https://doi.org/10.17632/97hj93mkfd.3`
  `https://zenodo.org/record/2841641`
- CWL run of Alignment Workflow (CWLProv 0.6.0 Research Object) [146]
  `https://doi.org/10.17632/6wtpgr3kbj.1`
  `https://zenodo.org/record/2632836`
- CWL run of RNA-seq Analysis Workflow (CWLProv 0.5.0 Research Object) [145]
  `https://doi.org/10.17632/xnwncxpw42.1`
  `https://zenodo.org/record/2838898`

The *CWLProv Python Tool* can be used to explore the above research objects.

Declarations

List of abbreviations

BAM: Binary Alignment Map; BCO: BioCompute Object; CRAM: Compressed Alignment Map; CWL: Common Workflow Language; EBI: European Bionformatics Institute; GATK: Genome Analysis ToolKit; HPC: High Performance Computing; JSON-LD: JavaScript Object Notation for Linked Data; OS: Operating System; PROV-DM: PROVenance Data Model; RO: Research Object; W3C: World Wide Web Consortium; WMS: Workflow Management System;

Ethical Approval (optional)

Not applicable.

Consent for publication

Not applicable.

Competing Interests

SSR and MRC are members of the leadership team for Common Workflow Language at the Software Freedom Conservancy.

Funding

FZK funded by Melbourne International Research Scholarship (MIRS) and Melbourne International Fee Remission Scholarship (MIFRS).

SSR and CG funded by BioExcel CoE, a project funded by the European Commission Horizon 2020 Framework Programme under contracts H2020-INFRAEDI-02-2018-823830 and H2020-EINFRA-2015-1-675728, as well as IBISBA (H2020-INFRAIA-1-2014-2015-730976).

Author's Contributions

Conceptualization: FZK, SSR, MRC. Data curation: FZK. Formal analysis: FZK. Funding acquisition: ROS, AL, CAG. Investigation: FZK. Methodology: FZK, SSR. Project administration: FZK, SSR, ROS, AL Computing Resources: ROS, AL. Software: FZK, SSR, MRC. Supervision: MRC, ROS, AL, CAG. Validation: FZK, SSR. Writing – original draft: FZK. Writing – review & editing: FZK, SSR, ROS, AL, MRC.

Acknowledgements

An earlier version of this article [157] was submitted for consideration at International Provenance and Annotation Workshop (IPAW) 2018. We would like to thank the IPAW reviewers for their constructive comments.

We would also like to thank the GigaScience editors and reviewers Tomoya Tanjo and Alban Gaignard for constructive and valuable feedback we think has improved the manuscript and future directions.

We would like to thank the Common Workflow Language community, and in particular Peter Amstutz, Pau Ruiz Safont and Pjotr Prins, for their continuing support, review and feedback. We would also like to thank Brad Chapman, Christopher Ball and Lon Blauvelt for the workflows used in the evaluation and their prompt replies to our enquiries.

We are grateful for partial travel support from Open Bioinformatics Foundation (OBF) Travel Fellowship Program [158] to Farah Zaib Khan for attending the Bioinformatics Open Source Conference (BOSC) 2017 and 2018 Codefests subsidizing this collaborative effort.

References

1. Stephens ZD, Lee SY, Faghri F, Campbell RH, Zhai C, Efron MJ, et al. Big data: astronomical or genomical? PLoS Biol 2015 jul; 13(7):e1002195, 10.1371/journal.pbio.1002195.
2. Atkinson M, Gesing S, Montagnat J, Taylor I. Scientific workflows: Past, present and future. Future Generation Computer Systems 2017 oct; 75:216–227, 10.1016/j.future.2017.05.041.
3. Spjuth O, Bongcam-Rudloff E, Hernández GC, Forer L, Giovacchini M, Guimera RV, et al. Experiences with workflows for automating data-intensive bioinformatics. Biology Direct 2015 aug; 10(1), 10.1186/s13062-015-0071-8.
4. Cuevas-Vicenttín V, Dey S, Köhler S, Riddle S, Ludäscher B. Scientific workflows and provenance: introduction and research opportunities. Datenbank Spektrum 2012 nov; 12(3):193–203, 10.1007/s13222-012-0100-z.
5. Common Workflow Language project, Community C, editor, Existing Workflow Systems. Common Workflow Language project; 2018. https://s.apache.org/existing-workflow-systems, accessed 12 Sep 2018.
6. Kanwal S, Khan FZ, Lonie A, Sinnott RO. Investigating reproducibility and tracking provenance – A genomic workflow case study. BMC Bioinformatics 2017 jul; 18(1):337, 10.1186/s12859-017-1747-0.
7. Wolstencroft K, Haines R, Fellows D, Williams A, Withers D, Owen S, et al. The Taverna workflow suite: designing and executing workflows of Web Services on the desktop, web or in the cloud. Nucleic Acids Res 2013 jul; 41(Web Server issue):W557–61, 10.1093/nar/gkt328.
8. Möller S, Prescott SW, Wirzenius L, Reinholdtsen P, Chapman B, Prins P, et al. Robust Cross-Platform Workflows: How Technical and Scientific Communities Collaborate to Develop, Test and Share Best Practices for Data Analysis. Data Sci Eng 2017 nov; 2(3):232–244, 10.1007/s41019-017-0050-4.
9. Alterovitz G, Dean D, Goble C, Crusoe MR, Soiland-Reyes S, Bell A, et al. Enabling precision medicine via standard communication of HTS provenance, analysis, and results. PLOS Biology 2019 12;16(12):1–14. , https://doi.org/10.1371/journal.pbio.3000099, 10.1371/journal.pbio.3000099.
10. Amstutz P, Crusoe MR, Khan FZ, Soiland-Reyes S, Singh M, kumar K, et al., common-workflow-language/cwltool: 1.0.20181012180214. Zenodo ; 2018, 10.5281/zenodo.1471589.
11. Amstutz P, Crusoe MR, Nebojša Tijanić, Chapman B, Chilton J, Heuer M, et al., Common Workflow Language, v1.0. Figshare ; 2016, 10.6084/m9.figshare.3115156.v2.
12. Ivie P, Thain D. Reproducibility in Scientific Computing. ACM Comput Surv 2018 Jul; 51(3):63:1–63:36, 10.1145/3186266.
13. Belhajjame K, Zhao J, Garijo D, Gamble M, Hettne K, Palma R, et al. Using a suite of ontologies for preserving workflow-centric research objects. Web Semantics: Science, Services and Agents on the World Wide Web 2015 may; 32(0):16–42, 10.1016/j.websem.2015.01.003.
14. Network Working Group, draft-kunze-bagit-17 – The BagIt File Packaging Format (V1.0); 2017. https://tools.ietf.org/html/draft-kunze-bagit-17, accessed 22 Sep 2018.
15. Missier P, Belhajjame K, Cheney J. The W3C PROV Family of Specifications for Modelling Provenance Metadata. In: Proceedings of the 16th International Conference on Extending Database Technology EDBT '13, New York, NY, USA: ACM; 2013. p. 773–776. , http://doi.acm.org/10.1145/2452376.2452478, 10.1145/2452376.2452478.
16. Hettne KM, Dharuri H, Zhao J, Wolstencroft K, Belhajjame K, Soiland-Reyes S, et al. Structuring research methods and data with the research object model: genomics workflows as a case study. J Biomed Semantics 2014 sep; 5(1):41, 10.1186/2041-1480-5-41.
17. Belhajjame K, Corcho O, Garijo D, Zhao J, Missier P, Newman D, et al. Workflow-centric research objects: First class citizens in scholarly discourse. In: Proceedings of the 2nd Workshop on Semantic Publishing (SePublica 2012), vol. 903 of CEUR Workshop Proceedings; 2012. p. 1–12. http://ceur-ws.org/Vol-903/paper-01.pdf.
18. Herschel M, Diestelkämper R, Ben Lahmar H. A survey on provenance: What for? What form? What from? VLDB J 2017 dec;26(6):881–906.
19. Moreau L, Missier P, Belhajjame K, B'Far R, Cheney J, Coppens S, et al., PROV-DM: The PROV Data Model; 2013. Http://www.w3.org/TR/2013/REC-prov-dm-20130430/.
20. Clifford B, Foster I, Voeckler JS, Wilde M, Zhao Y. Tracking provenance in a virtual data grid. Concurrency and Computation: Practice and Experience 2008 apr; 20(5):565–575, 10.1002/cpe.1256.
21. Casati F, Ceri S, Pernici B, Pozzi G. Workflow evolution. Data & Knowledge Engineering 1998 jan; 24(3):211–238, 10.1016/s0169-023x(97)00033-5.
22. interoperability | Definition of interoperability in English by Oxford Dictionaries;. https://en.oxforddictionaries.com/definition/interoperability, accessed 22 Sep 2018.
23. Tolk A. What Comes After the Semantic Web – PADS Implications for the Dynamic Web. In: 20th Workshop on Principles of Advanced and Distributed Simulation (PADS'06) IEEE;. , 10.1109/pads.2006.39.
24. Cohen-Boulakia S, Belhajjame K, Collin O, Chopard J, Froidevaux C, Gaignard A, et al. Scientific workflows for computational reproducibility in the life sciences: Status, challenges and opportunities. Future Generation Computer Systems 2017 oct; 75:284–298, 10.1016/j.future.2017.01.012.
25. Howe B. Virtual Appliances, Cloud Computing, and Reproducible Research. Computing in Science & Engineering 2012 jul;14(4):36–41. , https://doi.org/10.1109/mcse.2012.62, 10.1109/mcse.2012.62.
26. DigitalOcean – Cloud Computing, Simplicity at Scale;. https://www.digitalocean.com/, accessed 01 Dec 2018.
27. Amazon EC2;. https://aws.amazon.com/ec2/, accessed 01 Dec 2018.
28. Google Cloud including GCP & G Suite url = https://cloud.google.com/, note = Accessed 01 Dec 2018, urldate = 01/12/2018;.
29. Microsoft Azure Cloud Computing Platform & Services;. https://azure.microsoft.com/en-us/, accessed 01 Dec 2018.
30. Merkel D. Docker: Lightweight Linux Containers for Consistent Development and Deployment. Linux Journal 2014 Mar;2014(239). https://www.linuxjournal.com/node/1335702.
31. Kurtzer GM, Sochat V, Bauer MW. Singularity: Scientific containers for mobility of compute. PLoS ONE 2017 may; 12(5):e0177459, 10.1371/journal.pone.0177459.
32. Möller S, Prescott SW, Wirzenius L, Reinholdtsen P, Chapman B, Prins P, et al. Robust Cross-Platform Workflows: How Technical and Scientific Communities Collaborate to Develop, Test and Share Best Practices for Data Analysis. Data Science and Engineering 2017 Sep; 2(3):232–244, 10.1007/s41019-017-0050-4.
33. Grüning B, , Dale R, Sjödin A, Chapman BA, Rowe J, et al. Bioconda: sustainable and comprehensive software distribution for the life sciences. Nature Methods 2018 jul; 15(7):475–476, 10.1038/s41592-018-0046-7.
34. Conda –Conda documentation;. https://conda.io/docs/,

accessed 22 Sep 2018.
35. Zenodo - Research. Shared.;. https://zenodo.org/, accessed 22 Sep 2018.
36. GitHub;. https://github.com/, accessed 22 Sep 2018.
37. Goble CA, Bhagat J, Aleksejevs S, Cruickshank D, Michaelides D, Newman D, et al. myExperiment: a repository and social network for the sharing of bioinformatics workflows. Nucleic Acids Research 2010 may;38(suppl_2):W677–W682. , https://doi.org/10.1093/nar/gkq429, 10.1093/nar/gkq429.
38. figshare - credit for all your research;. https://figshare.com/, accessed 22 Sep 2018.
39. Code as a Research Object;. http://mozillascience.github.io/code-research-object/, accessed 22 Sep 2018.
40. Mozilla Science;. https://science.mozilla.org/, accessed 01 Dec 2018.
41. Chirigati F, Rampin R, Shasha D, Freire J. ReproZip. In: Proceedings of the 2016 International Conference on Management of Data - SIGMOD '16 ACM Press; 2016. , 10.1145/2882903.2899401.
42. Gomez-Perez JM, Palma R, Garcia-Silva A. Towards a Human-Machine Scientific Partnership Based on Semantically Rich Research Objects. In: 2017 IEEE 13th International Conference on e-Science (e-Science) IEEE; 2017. p. 266–275, 10.1109/eScience.2017.40.
43. Custovic A, Ainsworth J, Arshad H, Bishop C, Buchan I, Cullinan P, et al. The Study Team for Early Life Asthma Research (STELAR) consortium 'Asthma e-lab': team science bringing data, methods and investigators together. Thorax 2015; 70(8):799–801, 10.1136/thoraxjnl-2015-206781.
44. Moreau L, Freire J, Futrelle J, McGrath RE, Myers J, Paulson P. The Open Provenance Model: An Overview. In: Lecture Notes in Computer Science Springer Berlin Heidelberg; 2008. p. 323–326, 10.1007/978-3-540-89965-5_31.
45. Moreau L, Freire J, Futrelle J, Myers J, Paulson P. Governance of the open provenance model 2009 Jun;https://nms.kcl.ac.uk/luc.moreau/papers/governance.pdf, accessed 18 Sep 2018.
46. W3C Provenance Incubator Group Wiki - XG Provenance Wiki;. https://www.w3.org/2005/Incubator/prov/wiki/W3C_Provenance_Incubator_Group_Wiki, accessed 22 Sep 2018.
47. Moreau L, Groth P, Cheney J, Lebo T, Miles S. The rationale of PROV. Web Semantics: Science, Services and Agents on the World Wide Web 2015 dec; 35:235–257, 10.1016/j.websem.2015.04.001.
48. Michaelides DT, Parker R, Charlton C, Browne WJ, Moreau L. Intermediate notation for provenance and workflow reproducibility. In: Mattoso M, Glavic B, editors. Provenance and annotation of data and processes, vol. 9672 of Lecture notes in computer science Cham: Springer International Publishing; 2016. p. 83–94, 10.1007/978-3-319-40593-3_7.
49. Pasquier T, Han X, Goldstein M, Moyer T, Eyers D, Seltzer M, et al. Practical whole-system provenance capture. In: Proceedings of the 2017 Symposium on Cloud Computing - SoCC '17 New York, New York, USA: ACM Press; 2017. p. 405–418, 10.1145/3127479.3129249.
50. Giesler A, Czekala M, Hagemeier B, Grunzke R. Uniprov: A flexible provenance tracking system for UNICORE. In: Di Napoli E, Hermanns MA, Iliev H, Lintermann A, Peyser A, editors. High-Performance Scientific Computing, vol. 10164 of Lecture notes in computer science Cham: Springer International Publishing; 2017. p. 233–242, 10.1007/978-3-319-53862-4_20.
51. Benabdelkader A, VanKampen AA, Olabarriaga SD. PROVman: A PROV-compliant toolkit for provenance management. PeerJ PrePrints 2015; 3(e1102v1), 10.7287/peerj.preprints.1102v1.
52. Gaignard A, Skaf-Molli H, Bihouée A. From Scientific Workflow Patterns to 5-star Linked Open Data. In: TaPP; 2016. .
53. Clark T, Ciccarese PN, Goble CA. Micropublications: a semantic model for claims, evidence, arguments and annotations in biomedical communications. Journal of Biomedical Semantics 2014;5(1):28. , https://doi.org/10.1186/2041-1480-5-28, 10.1186/2041-1480-5-28.
54. Soiland-Reyes S, Alper P, Goble C. Tracking workflow execution with TavernaProv. PROV Three Years Later; workshop at Provenance Week 2016, McLean, Virginia, USA.2016 jun;, 10.5281/zenodo.51314.
55. Freire J, Silva CT. Making Computations and Publications Reproducible with VisTrails. Comput Sci Eng 2012 jul; 14(4):18–25, 10.1109/{MCSE}.2012.76.
56. Gil Y, Ratnakar V, Kim J, Gonzalez-Calero P, Groth P, Moody J, et al. Wings: Intelligent Workflow-Based Design of Computational Experiments. IEEE Intelligent Systems 2011 Jan; 26(1):62–72, 10.1109/MIS.2010.9.
57. Garijo D, Gil Y, Corcho O. Abstract, link, publish, exploit: An end to end framework for workflow sharing. Future Generation Computer Systems 2017 oct; 75:271–283, 10.1016/j.future.2017.01.008.
58. Nekrutenko A, Taylor J. Next-generation sequencing data interpretation: enhancing reproducibility and accessibility. Nature Reviews Genetics 2012 sep; 13(9):667–672, 10.1038/nrg3305.
59. Garijo D, Kinnings S, Xie L, Xie L, Zhang Y, Bourne PE, et al. Quantifying reproducibility in computational biology: the case of the tuberculosis drugome. PLoS ONE 2013 nov; 8(11):e80278, 10.1371/journal.pone.0080278.
60. Sandve GK, Nekrutenko A, Taylor J, Hovig E. Ten simple rules for reproducible computational research. PLoS Comput Biol 2013 oct; 9(10):e1003285, 10.1371/journal.pcbi.1003285.
61. Mohan A, Lu S, Kotov A. Addressing the Shimming Problem in Big Data Scientific Workflows. In: 2014 IEEE International Conference on Services Computing IEEE; 2014. , 10.1109/scc.2014.53.
62. Littauer R, Ram K, Ludäscher B, Michener W, Koskela R. Trends in Use of Scientific Workflows: Insights from a Public Repository and Recommendations for Best Practice. International Journal of Digital Curation 2012 oct; 7(2):92–100, 10.2218/ijdc.v7i2.232.
63. Stodden V, McNutt M, Bailey DH, Deelman E, Gil Y, Hanson B, et al. Enhancing reproducibility for computational methods. Science 2016 dec; 354(6317):1240–1241, 10.1126/science.aah6168.
64. Stodden V, Miguez S. Best Practices for Computational Science: Software Infrastructure and Environments for Reproducible and Extensible Research. Journal of Open Research Software 2014 jul; 2(1), 10.5334/jors.ay.
65. Zhao J, Gomez-Perez JM, Belhajjame K, Klyne G, Garcia-Cuesta E, Garrido A, et al. Why workflows break -Understanding and combating decay in Taverna workflows. In: 2012 IEEE 8th International Conference on E-Science IEEE; 2012. , 10.1109/escience.2012.6404482.
66. Gymrek M, Farjoun Y. Recommendations for open data science. GigaScience 2016 may; 5(1), 10.1186/s13742-016-0127-4.
67. Wilkinson MD, Dumontier M, Aalbersberg IJJ, Appleton G, Axton M, Baak A, et al. The FAIR Guiding Principles for scientific data management and stewardship. Sci Data 2016 mar; 3:160018, 10.1038/sdata.2016.18.
68. Ludäscher B. A Brief Tour Through Provenance in Scientific Workflows and Databases. Springer Proceedings in Business and Economics 2016; p. 103–126, 10.1007/

978-3-319-40226-0_7.
69. Bubak M, Kasztelnik M, Malawski M, Meizner J, Nowakowski P, Varma S. Evaluation of Cloud Providers for VPH Applications. 2013 13th IEEE/ACM International Symposium on Cluster, Cloud, and Grid Computing 2013 May;, 10.1109/ccgrid.2013.54.
70. Angiuoli SV, White JR, Matalka M, White O, Fricke WF. Resources and Costs for Microbial Sequence Analysis Evaluated Using Virtual Machines and Cloud Computing. PLoS ONE 2011 Oct; 6(10):e26624, 10.1371/journal.pone.0026624.
71. Chen W, Deelman E. Partitioning and scheduling workflows across multiple sites with storage constraints. In: International Conference on Parallel Processing and Applied Mathematics Springer; 2011. p. 11–20, 10.1007/978-3-642-31500-8_2.
72. GATK | BP Doc #11165 | Data pre-processing for variant discovery; 2018. https://software.broadinstitute.org/gatk/best-practices/workflow?id=11165, accessed 22 Sep 2018.
73. Malawski M, Figiela K, Nabrzyski J. Cost minimization for computational applications on hybrid cloud infrastructures. Future Generation Computer Systems 2013 Sep; 29(7):1786–1794, 10.1016/j.future.2013.01.004.
74. Alper P, Belhajjame K, Curcin V, Goble C. LabelFlow Framework for Annotating Workflow Provenance. Informatics 2018 feb; 5(1):11, 10.3390/informatics5010011.
75. Gaignard A, Montagnat J, Gibaud B, Forestier G, Glatard T. Domain-specific summarization of Life-Science e-experiments from provenance traces. Journal of Web Semantics 2014 Dec;29:19–30. , https://doi.org/10.1016/j.websem.2014.07.001, 10.1016/j.websem.2014.07.001.
76. Ison J, Kalas M, Jonassen I, Bolser D, Uludag M, McWilliam H, et al. EDAM: an ontology of bioinformatics operations, types of data and identifiers, topics and formats. Bioinformatics 2013 mar; 29(10):1325–1332, 10.1093/bioinformatics/btt113.
77. Michel F, Community TB. Bioschemas amp; Schema.org: a Lightweight Semantic Layer for Life Sciences Websites. Biodiversity Information Science and Standards 2018 May; 2:e25836, 10.3897/biss.2.25836.
78. Garijo D, Gil Y, Corcho O. Towards Workflow Ecosystems through Semantic and Standard Representations. In: 2014 9th Workshop on Workflows in Support of Large-Scale Science IEEE; 2014. p. 94–104, 10.1109/{WORKS}.2014.13.
79. Sefton P, Lynch M, Devine G, Loxton D. DataCrate: a method of packaging, distributing, displaying and archiving Research Objects Workshop on Research Objects (RO2018) at IEEE eScience 20182018 Jul;, 10.5281/zenodo.1312323.
80. Woods C, BioExcel Webinar 28: BioSimSpace – filling the gaps between molecular simulation codes. YouTube, BioExcel; 2018. https://youtu.be/pD8mhj3WElE?t=1599.
81. BioSimSpace; 2018. https://biosimspace.org/, accessed 29 Nov 2018.
82. Mitchell AL, Scheremetjew M, Denise H, Potter S, Tarkowska A, Qureshi M, et al. EBI Metagenomics in 2017: enriching the analysis of microbial communities, from sequence reads to assemblies. Nucleic Acids Research 2018; 46(D1):D726–D735, 10.1093/nar/gkx967.
83. Kim JG, et al MH, 5 Star Open Data;. https://5stardata.info/, accessed 21 May 2019.
84. Soiland-Reyes S, Khan FZ, Crusoe MR, common-workflow-language/cwlprov: CWLProv 0.6.0. Zenodo ; 2018, 10.5281/zenodo.1471585.
85. Lóscio BF, Burle C, Calegari N, Isaac AGA, Iglesias C, Laufer C, et al., Data on the Web Best Practices; 2017. https://www.w3.org/TR/2017/REC-dwbp-20170131/#ReuseVocabularies, w3C Recommendation 31 January 2017.
86. Kaushik G, Ivkovic S, Simonovic J, Tijanic N, Davis-Dusenbery B, Kural D. Rabix: an open-source workflow executor supporting recomputability and interoperability of workflow descriptions. Pac Symp Biocomput 2017; 22:154–165, 10.1142/9789813207813_0016.
87. Voss K, Auwera GVD, Gentry J, Full-stack genomics pipelining with GATK4 + WDL + Cromwell. F1000Research ; 2017, 10.7490/f1000research.1114634.1.
88. Guimera RV. bcbio-nextgen: Automated, distributed next-gen sequencing pipeline. EMBnet j 2012 feb; 17(B):30, 10.14806/ej.17.B.286.
89. Bechhofer S, Buchan I, De Roure D, Missier P, Ainsworth J, Bhagat J, et al. Why linked data is not enough for scientists. Future Gener Comput Syst 2013;29(2):599–611.
90. researchobject.org;. http://www.researchobject.org/overview/, accessed 22 Sep 2018.
91. Chard K, D'Arcy M, Heavner B, Foster I, Kesselman C, Madduri R, et al. I'll take that to go: Big data bags and minimal identifiers for exchange of large, complex datasets. In: 2016 IEEE International Conference on Big Data (Big Data) IEEE; 2016. p. 319–328, 10.1109/bigdata.2016.7840618.
92. Gil Y, Miles S, Belhajjame K, Deus H, Garijo D, Klyne G, et al., PROV Model Primer; 2013. http://www.w3.org/TR/2013/NOTE-prov-primer-20130430/, w3C Working Group Note 30 April 2013.
93. Moreau L, Missier P, Cheney J, Soiland-Reyes S, PROV-N: The Provenance Notation; 2013. http://www.w3.org/TR/2013/REC-prov-n-20130430/, w3C Recommendation 30 April 2013.
94. Huynh TD, Jewell M, Keshavarz AS, Michaelides D, Yang H, Moreau L, The PROV-JSON Serialization. A JSON Representation for the PROV Data Model; 2013. http://www.w3.org/Submission/2013/SUBM-prov-json-20130424/, w3C Member Submission 24 April 2013.
95. Hua H, Tilmes C, Zednik S, Moreau L, PROV-XML: The PROV XML Schema; 2013. http://www.w3.org/TR/2013/NOTE-prov-xml-20130430/, w3C Working Group Note 30 April 2013.
96. Lebo T, Sahoo S, McGuinness D, Belhajjame K, Cheney J, Corsar D, et al., PROV-O: The PROV Ontology; 2013. http://www.w3.org/TR/2013/REC-prov-o-20130430/, w3C Recommendation Note 30 April 2013.
97. EMC Education Services. Information Storage and Management: Storing, Managing, and Protecting Digital Information in Classic, Virtualized, and Cloud Environments, Second Edition. Gnanasundaram S, Shrivastava A, editors, John Wiley & Sons; 2012. ISBN 978-1-118-09483-9.
98. Soiland-Reyes S, Gamble M, Haines R. Research Object Bundle 1.0 2014;, 10.5281/zenodo.12586.
99. Sporny M, Longley D, Kellogg G, Lanthaler M, Lindström N, JSON-LD 1.0: A JSON-based Serialization for Linked Data; 2014. http://www.w3.org/TR/2014/REC-json-ld-20140116/, w3C Recommendation 16 January 2014.
100. Ohta T, Tanjo T, Ogasawara O. Accumulating computational resource usage of genomic data analysis workflow to optimize cloud computing instance selection. GigaScience 2019 04; 8(4), 10.1093/gigascience/giz052.
101. Soiland-Reyes S, Bechhofer S, Corcho O, Klyne G, Belhajjame K, Garijo D, et al., The Wfdesc Ontology; 2016. https://w3id.org/ro/2016-01-28/wfdesc, accessed 22 Sep 2018.
102. Soiland-Reyes S, Bechhofer S, Corcho O, Klyne G, Belhajjame K, Garijo D, et al., The Wfprov Ontology; 2016.

https://w3id.org/ro/2016-01-28/wfprov/, accessed 22 Sep 2018.
103. Cao Y, Jones C, Cuevas-Vicenttın V, Jones MB, Ludäscher B, McPhillips T, et al. ProvONE: Extending PROV to support the DataONE scientific community 2016 jun;http://homepages.cs.ncl.ac.uk/paolo.missier/doc/dataone-prov-3-years-later.pdf.
104. Soiland-Reyes S, Cáceres M. The Archive and Package (arcp) URI scheme. Internet-Draft soilandreyes-arcp 2018 jan;https://tools.ietf.org/id/draft-soilandreyes-arcp-03.html.
105. Soiland-Reyes S, Cáceres M. The Archive and Package (arcp) URI scheme. In: 2018 IEEE 13th International Conference on e-Science (e-Science); 2018. http://s11.no/2018/arcp.html, in Print, arXiv:1809.06935.
106. prov 1.5.2; 2018. https://pypi.org/project/prov/, accessed 22 Sep 2018.
107. Vivian J, Rao AA, Nothaft FA, Ketchum C, Armstrong J, Novak A, et al. Toil enables reproducible, open source, big biomedical data analyses. Nature biotechnology 2017; 35(4):314, 10.1038/nbt.3772.
108. Arvados - Open Source Big Data Processing and Bioinformatics;. https://arvados.org/, accessed 08 Nov 2018.
109. Kotliar M, Kartashov A, Barski A. CWL-Airflow: a lightweight pipeline manager supporting Common Workflow Language. bioRxiv 2018;, 10.1101/249243.
110. common-workflow-language/cwltool:Common Workflow Language reference implementation; 2016. https://github.com/common-workflow-language/cwltool#cwl-tool-control-flow, accessed 23 Sep 2018.
111. Di Tommaso P, Chatzou M, Floden EW, Barja PP, Palumbo E, Notredame C. Nextflow enables reproducible computational workflows. Nat Biotechnol 2017 apr; 35(4):316–319, 10.1038/nbt.3820.
112. Centre for Genomic Regulation (CRG), Nextflow: Tracing and visualization; 2018. https://www.nextflow.io/docs/latest/tracing.html#trace-report, accessed 28 Nov 2018.
113. Ohta T, Tanjo T, Ogasawara O. Accumulating computational resource usage of genomic data analysis workflow to optimize cloud computing instance selection. bioRxiv 2018;, 10.1101/456756.
114. Cristofori A, Bologna I, Gordon J, London SR, Kennedy J, Munich R, et al. Usage Record–Format Recommendation. In: Open Grid Forum; 2013. https://www.ogf.org/documents/GFD.204.pdf, gFD-R-P.204.
115. Robinson M, Soiland-Reyes S, Crusoe MR, Goble C. Common Workflow Language Viewer; 2017. https://view.commonwl.org/.
116. Dobin A, Gingeras TR. Mapping RNA-seq reads with STAR. Current Protocols in Bioinformatics 2015; 51(1):11–14, 10.1002/0471250953.bi1114s51.
117. Conesa A, Madrigal P, Tarazona S, Gomez-Cabrero D, Cervera A, McPherson A, et al. A survey of best practices for RNA-seq data analysis. Genome Biology 2016 jan; 17(1), 10.1186/s13059-016-0881-8.
118. TopMed RNA-seq workflow; 2018. https://w3id.org/cwl/view/git/027e8af41b906173aafdb791351fb29efc044120/topmed-workflows/TOPMed_RNAseq_pipeline/rnaseq_pipeline_fastq.cwl, accessed 23 Sep 2018.
119. heliumdatacommons; 2017. https://github.com/heliumdatacommons, accessed 23 Sep 2018.
120. Data Commons | NIH Common Fund; 2018. https://commonfund.nih.gov/commons, accessed 23 Sep 2018.
121. heliumdatacommons/cwl_workflows: Example CWL Workflows that run on team Helium PIVOT architecture.;. https://github.com/heliumdatacommons/cwl_workflows, accessed 03 Oct 2018.
122. Trans-Omics for Precision Medicine (TOPMed) Program | National Heart, Lung, and Blood Institute (NHLBI); 2014. https://www.nhlbi.nih.gov/science/trans-omics-precision-medicine-topmed-program, accessed 23 Sep 2018.
123. Gtex RNA-seq pipeline; 2017. https://github.com/broadinstitute/gtex-pipeline/tree/master/rnaseq, accessed 23 Sep 2018.
124. Dobin A, Davis CA, Schlesinger F, Drenkow J, Zaleski C, Jha S, et al. STAR: ultrafast universal RNA-seq aligner. Bioinformatics 2012 oct;29(1):15–21. , https://doi.org/10.1093/bioinformatics/bts635, 10.1093/bioinformatics/bts635.
125. Tool documentation: MarkDuplicates;. http://broadinstitute.github.io/picard/command-line-overview.html#MarkDuplicates, accessed 23 Sep 2018.
126. Li H, Handsaker B, Wysoker A, Fennell T, Ruan J, Homer N, et al. The sequence alignment/map format and SAMtools. Bioinformatics 2009; 25(16):2078–2079, 10.1093/bioinformatics/btp352.
127. DeLuca DS, Levin JZ, Sivachenko A, Fennell T, Nazaire MD, Williams C, et al. RNA-SeQC: RNA-seq metrics for quality control and process optimization. Bioinformatics 2012 apr; 28(11):1530–1532, 10.1093/bioinformatics/bts196.
128. Li B, Dewey CN. RSEM: accurate transcript quantification from RNA-Seq data with or without a reference genome. BMC Bioinformatics 2011; 12(1):323, 10.1186/1471-2105-12-323.
129. Seo JS, Ju YS, Lee WC, Shin JY, Lee JK, Bleazard T, et al. The transcriptional landscape and mutational profile of lung adenocarcinoma. Genome Research 2012;, 10.1101/gr.145144.112.
130. Xu C. A review of somatic single nucleotide variant calling algorithms for next-generation sequencing data. Computational and structural biotechnology journal 2018;, 10.1016/j.csbj.2018.01.003.
131. topmed-workflows/topmed-alignment.cwl at cwl-prov_testing · FarahZKhan/topmed-workflows;. https://github.com/FarahZKhan/topmed-workflows/blob/2fcea0b9469b572399755b6828ff87a40d865e43/aligner/sbg-alignment-cwl/topmed-alignment.cwl, accessed 23 Sep 2018.
132. Data Biosphere; 2018. https://github.com/DataBioSphere, accessed 23 Sep 2018.
133. statgen/docker-alignment: Dockerfile for Alignment; 2017. https://github.com/statgen/docker-alignment, accessed 23 Sep 2018.
134. Abecasis Lab - Genome Analysis Wiki; 2017. https://genome.sph.umich.edu/wiki/Abecasis_Lab, accessed 23 Sep 2018.
135. Cochrane G, Alako B, Amid C, Bower L, Cerdeño-Tárraga A, Cleland I, et al. Facing growth in the European Nucleotide Archive. Nucleic Acids Research 2012 nov;41(D1):D30–D35. , https://doi.org/10.1093/nar/gks1175, 10.1093/nar/gks1175.
136. Li H. Aligning sequence reads, clone sequences and assembly contigs with BWA-MEM. arXiv preprint arXiv:13033997 2013;https://arxiv.org/abs/1303.3997.
137. Faust GG, Hall IM. SAMBLASTER: fast duplicate marking and structural variant read extraction. Bioinformatics 2014 may;30(17):2503–2505. , https://doi.org/10.1093/bioinformatics/btu314, 10.1093/bioinformatics/btu314.
138. Introduction to Variant Calling; 2014. https://bioconductor.org/help/course-materials/2014/CSAMA2014/3_Wednesday/lectures/VariantCallingLecture.pdf, accessed 23 Sep 2018.
139. Saunders CT, Wong WSW, Swamy S, Becq J, Murray LJ, Cheetham RK. Strelka: accurate somatic small-variant calling from sequenced tumor–normal sample

pairs. Bioinformatics 2012 may; 28(14):1811–1817, 10.1093/bioinformatics/bts271.

140. Blue Collar Bioinformatics;. http://bcb.io/, accessed 23 Sep 2018.
141. Somatic Variant Calling Workflow; 2018. https://github.com/FarahZKhan/bcbio_test_cwlprov/blob/master/somatic/somatic-workflow/main-somatic.cwl, accessed 23 Sep 2018.
142. Common Workflow Language (CWL) -bcbio-nextgen 1.1.0 documentation; 2017. https://bcbio-nextgen.readthedocs.io/en/latest/contents/cwl.html#current-status, accessed 23 Sep 2018.
143. Nectar Cloud - Nectar;. https://nectar.org.au/research-cloud/, accessed 23 Sep 2018.
144. Kanwal S, Lonie A, Sinnott RO. Digital reproducibility requirements of computational genomic workflows. In: Bioinformatics and Biomedicine (BIBM), 2017 IEEE International Conference on IEEE; 2017. p. 1522–1529, 10.1109/bibm.2017.8217887.
145. Khan FZ, Soiland-Reyes S, CWL run of RNA-seq Analysis Workflow (CWLProv 0.5.0 Research Object)). Mendeley DataV1. ; 2018, 10.17632/xnwncxpw42.1.
146. Khan FZ, Soiland-Reyes S, CWL run of Alignment Workflow (CWLProv 0.6.0 Research Object). Mendeley DataV1. ; 2018, 10.17632/6wtpgr3kbj.1.
147. Khan FZ, Soiland-Reyes S, CWL run of Somatic Variant Calling Workflow (CWLProv 0.5.0 Research Object). Mendeley DataV3. ; 2018, 10.17632/97hj93mkfd.3.
148. Gruening B, Sallou O, Moreno P, da Veiga Leprevost F, Ménager H, Søndergaard D, et al. Recommendations for the packaging and containerizing of bioinformatics software. F1000Research 2018 jun;7:742. , https://doi.org/10.12688/f1000research.15140.1, 10.12688/f1000research.15140.1.
149. Missier P. The Lifecycle of Provenance Metadata and Its Associated Challenges and Opportunities. In: Building Trust in Information Springer International Publishing; 2016.p. 127–137. , https://doi.org/10.1007/978-3-319-40226-0_8, 10.1007/978-3-319-40226-0_8.
150. Soiland-Reyes S, Khan FZ, common-workflow-language/cwlprov-py: cwlprov-py 0.1.1. Zenodo; 2018. , https://doi.org/10.5281/zenodo.1471376, 10.5281/zenodo.1471376.
151. Carata L, Akoush S, Balakrishnan N, Bytheway T, Sohan R, Selter M, et al. A primer on provenance. Communications of the ACM 2014 may;57(5):52–60. , https://doi.org/10.1145/2596628, 10.1145/2596628.
152. Kim D, Vouk MA. Assessing Run-time Overhead of Securing Kepler. Procedia Computer Science 2016; 80:2281–2286, 10.1016/j.procs.2016.05.412.
153. Madduri R, Chard K, D'Arcy M, Jung SC, Rodriguez A, Sulakhe D, et al. Reproducible big data science: A case study in continuous FAIRness. PLOS ONE 2019 04;14(4):1–22. , https://doi.org/10.1371/journal.pone.0213013, 10.1371/journal.pone.0213013.
154. Goble C. Better Software, Better Research. IEEE Internet Computing 2014 sep;18(5):4–8. , https://doi.org/10.1109/mic.2014.88, 10.1109/mic.2014.88.
155. Software Carpentry;. https://software-carpentry.org/, accessed 03 Oct 2018.
156. Code Is Science;. http://www.codeisscience.com/, accessed 03 Oct 2018.
157. Khan FZ, Soiland-Reyes S, Crusoe MR, Lonie A, Sinnott R. CWLProv - Interoperable Retrospective Provenance capture and its challenges Zenodo preprint2018 mar;, 10.5281/zenodo.1215611.
158. OBF Travel Fellowship Program | OBF News; 2016. https://news.open-bio.org/2016/03/01/obf-travel-fellowship-program/, accessed 26 Sep 2018.

Response to reviewers

Click here to access/download
Supplementary Material
Response_to_Reviewers.pdf

Cover Letter Click here to access/download;Personal Cover;coverletter.pdf

Cover Letter

Dear Scott,

We are writing to submit a revised manuscript titled as "Sharing interoperable workflow provenance: A review of best practices and their practical application in CWLProv" to be considered for publication in GigaScience.

Our work focuses on provenance documentation and standardised representation of bioinformatics workflows to achieve interoperability and reproducibility of workflow-centric data-intensive computational experiments. We have reviewed the existing literature dedicated to devise best practices for workflow design, provenance documentation, analysis sharing and publishing. In the light of these community-driven recommendations, we have devised a generalised hierarchical provenance framework to achieve uniformity in the provenance granularity and completeness in analysis sharing. We have also realized this framework and devised CWLProv - A format for CWL workflow enactment representation using open-source well-defined standards for workflow definition, method aggregation and provenance representation.

As our contributions can be generalized to any computational scientific workflow-centric study, these are likely to be of interest to wide range of scientific community. The key principles of our research align with the goals of GigaScience i.e. "promoting reproducibility of analyses and data dissemination, organization, understanding, and use". We believe GigaScience is the most appropriate place for publishing our research.

All authors have approved the manuscript for submission. The work represents original research which is not under consideration by any other journal. We also declare that the content of this manuscript has not been published elsewhere. However, an earlier version with preliminary implementation was submitted to IPAW-2018 (10.5281/zenodo.1208478) but was not accepted.

We thank the reviewers for their valuable feedback and GigaScience for giving us this opportunity to implement the changes suggested by the reviewers. We forward hearing from you in due course.

Date: 23/05/2019

Yours faithfully,
Farah Zaib Khan
Stian Soiland-Reyes
Richard O. Sinnott
Andrew Lonie
Carole Goble
Michael R. Crusoe
