# Supplementary material for: Sharing interoperable workflow provenance: A review of best practices and their practical application in CWLProv
Source: Gigascience. 2019 Nov 1;8(11):giz095. doi: 10.1093/gigascience/giz095 (PMC6824458; doi:10.1093/gigascience/giz095)
Supplement: giz095_Response_to_Reviewer_Comments_Original_Submission [file giz095_response_to_reviewer_comments_original_submission.pdf]

Author's Response To Reviewer Comments

Close

Hi,
We have provided detailed responses in a pdf document (attached as supplementary material).
Please refer to that.
Cheers,

Close
