# Supplementary material for: Sharing interoperable workflow provenance: A review of best practices and their practical application in CWLProv
Source: Gigascience. 2019 Nov 1;8(11):giz095. doi: 10.1093/gigascience/giz095 (PMC6824458; doi:10.1093/gigascience/giz095)
Supplement: giz095_Reviewer_1_Report_Original_Submission — Tomoya Tanjo, Ph.D. -- 1/5/2019 Reviewed [file giz095_reviewer_1_report_original_submission.pdf]

Reviewer Report

Title: Sharing interoperable workflow provenance: A review of best practices and their practical application in CWLProv

Version: Original Submission **Date:** 1/5/2019

Reviewer name: Tomoya Tanjo, Ph.D.

Reviewer Comments to Author:

This paper summarizes the best practice recommendations for computation analysis from literature and defines a set of levels of provenance and resource sharing. Also, the authors define `CWLProv` that is based on the defined levels of recommendations to provide interoperability between different workflow platforms. The evaluation results show that it enables several levels of interoperability between several workflow platforms and operating systems.
Their proposed set of levels of provenance and summary of recommendation will be helpful for developers of workflow platforms as well as users of workflow platforms because now we can discuss how higher level of provenance our platform (or our workflow) support.
This paper is well structured and well written but there is a point to be addressed in the evaluation. Table 5 says that the enactment of Alignment Workflow with `cwltool` with enabling provenance capture on MacOS could not be tested due to insufficient hardware resources. Does it mean that the step (I) in `Evaluation Activity` for Alignment Workflow could not be executed? If so, please clarify it.
Here are minor things to be addressed:
- Sometimes `CWLProv` and its following word are accidentally concatenated.
 - e.g, p2. line 13 or 14 "CWLProvoutcome", p2. line 32 "CWLProv0.6.0"
- Figure 1 uses the spelling `artifacts` in level 1 but this paper mainly uses
 `artefacts`. It is better to use a consistent spelling.
- The left side of Figure 2 shows a GATK workflow but the caption says the right side is a workflow.
- Table 5 says that the enactment of Somatic Variant Calling
 Workflow with `toil-cwl-runner` due to a known bug. However, the link in the table
 is for a issue of `cwltool`, not `toil-cwl-runner`. I got confused
 because the enactment of the same workflow with `cwltool` works.
 If the linked issue is occurred in `toil-cwl-runner` for the
 variant calling workflow, I recommend to make a link to the issue of
 `toil-cwl-nurner` instead of `cwltool`. It is less confusing.

Methods

Are the methods appropriate to the aims of the study, are they well described, and are necessary controls included? Choose an item.

Conclusions

Are the conclusions adequately supported by the data shown? Choose an item.

Reporting Standards

Does the manuscript adhere to the journal's guidelines on minimum standards of reporting? Choose an item.

Choose an item.

Statistics

Are you able to assess all statistics in the manuscript, including the appropriateness of statistical tests used? Choose an item.

Quality of Written English

Please indicate the quality of language in the manuscript: Choose an item.

Declaration of Competing Interests

Please complete a declaration of competing interests, considering the following questions:

- Have you in the past five years received reimbursements, fees, funding, or salary from an organisation that may in any way gain or lose financially from the publication of this manuscript, either now or in the future?
- Do you hold any stocks or shares in an organisation that may in any way gain or lose financially from the publication of this manuscript, either now or in the future?
- Do you hold or are you currently applying for any patents relating to the content of the manuscript?
- Have you received reimbursements, fees, funding, or salary from an organization that holds or has applied for patents relating to the content of the manuscript?
- Do you have any other financial competing interests?
- Do you have any non-financial competing interests in relation to this paper?

If you can answer no to all of the above, write 'I declare that I have no competing interests' below. If your reply is yes to any, please give details below.

I declare that I have no competing interests

I agree to the open peer review policy of the journal. I understand that my name will be included on my report to the authors and, if the manuscript is accepted for publication, my named report including any attachments I upload will be posted on the website along with the authors' responses. I agree for my report to be made available under an Open Access Creative Commons CC-BY license (http://creativecommons.org/licenses/by/4.0/). I understand that any comments which I do not wish to be included in my named report can be included as confidential comments to the editors, which will not be published.

Choose an item.

To further support our reviewers, we have joined with Publons, where you can gain additional credit to further highlight your hard work (see: https://publons.com/journal/530/gigascience). On publication of this paper, your review will be automatically added to Publons, you can then choose whether or not to claim your Publons credit. I understand this statement.

Yes Choose an item.
