# Supplementary material for: Sharing interoperable workflow provenance: A review of best practices and their practical application in CWLProv
Source: Gigascience. 2019 Nov 1;8(11):giz095. doi: 10.1093/gigascience/giz095 (PMC6824458; doi:10.1093/gigascience/giz095)
Supplement: giz095_Reviewer_2_Report_Original_Submission — Alban Gaignard -- 1/22/2019 Reviewed [file giz095_reviewer_2_report_original_submission.pdf]

Reviewer Report

Title: Sharing interoperable workflow provenance: A review of best practices and their practical application in CWLProv

Version: Original Submission **Date:** 1/22/2019

Reviewer name: Alban Gaignard

Reviewer Comments to Author:

Review "Sharing interoperable workflow provenance: a review of best practices and their practical application in CWLProv"

This paper proposes an in-depth, extensive, literature review in the field of reproducible computational sciences as well as a prototype implementation leveraging state-of-the-art standardization efforts.

Main issues identified are the incompleteness of captured provenance information and the lack of interoperability. Even if CWL is emerging as a standard and system-independent language to represent scientific workflows, there is no standards to represent, share, and reproduce computational analysis as workflow executions. As a result of the literature review, the authors propose (1) a broad list of requirements for better reproducibility, and (2) a hierarchy of provenance levels addressing various needs, from workflow debug, workflow re-execution and packaging, towards better re-use and re-purposing for scientists, based on domain-specific annotated provenance. Then, the CWLProv implementation is described and evaluated on 3 real-life bioinformatics workflows.

My main concern regarding this work is that it is often stated that the re-usability of workflow resources (methods / input or output data) is facilitated but it is difficult to evaluate this claim based on CWLProv features and the proposed experiments. It is clear that re-execution of workflows is facilitated but it is unclear to what extent produced/analysed data can be considered for secondary use. In addition, the "pragmatic" interoperability should refer to top-level provenance and thus domain-specific annotations referring to the scientific context of the computational experiment. The experiments don't clearly show how CWLprov goes into the direction of (still ambitious and challenging) domain-specific provenance.

I've also a technical concern regarding the FAIRness of the approach since some of the requirements could be addressed following the (5-star) Linked Data principles. This point should be addressed in the discussion.

Finally, I tried to browse the research objects provided as supporting material but unfortunately I could not access the resource. Logs are provided at the end of the review.

Apart from that, the paper is well written (sometime a bit long) and well illustrated. The structure is easy to follow. This paper results from an impressive and high-quality work for a very timely topic, with urgent community needs. It should be published, after minor clarifications and corrections.

Detailed comments

Introduction

In Key Points, 4th point, a space is missing in "CWLProvoutcome"

Background and related works

    - The first paragraph of related works is too long.
    - "co-installability" -> what does it means ?
    - "They explore five essential requirements" -> why not briefly describing them ?
    - Some references could be added to works addressing the sharing of domain-specific annotated provenance, for instance, https://doi.org/10.1186/2041-1480-5-28, https://doi.org/10.1016/j.websem.2014.07.001, or "From Scientific Workflow Patterns to 5-star Linked Open Data" in TaPP'16.
Levels of Provenance and resource sharing
    - "… in Figure 1 that all WMs can benefit from and conform to without additional technical overhead" -> difficult to believe that there is no technical overhead
    - Table 1 -> the list of recommendations is quite long, some of the recommendations are overlapping (R9 and R19 could be merged, as well as R6 and R7). Grouping them, possibly through the proposed levels could ease the reading and understanding of these recommendations. In addition R18 is a too vague.
    - Figure1 -> in Level 0 "Results interpretation is questionable", scientists will need some context (Level 3) to understand the produced results, he/she may be lost in all fine-grained provenance, and extracting important parameters would certainly be time-consuming and require technical expertise.
    - R2, R13, R16-18 are not mentioned in the Levels0-3 descriptions.
    - Level 2 paragraph 2 : Re-enactment -> this feature already exists in make-like systems, such as snakemake, actively developed and used in the bioinformatics community.
    - Level 2 : "meaningful for a user" -> which kind of user ?

CWLProv 0.6.0
    - "we have reused the BDBag approach based on BagIt" -> a short example of a Bag would have been useful.
    - "We utilise mainly two serialisations of PROV […]" -> why not using PROV-O to ease the linking of provenance information to other datasets as well as its analytics through querying or logical reasoning. This would also enhance findability on the web. This point should be part of the discussion.
    - "workflow/" -> the paragraph on "executable workflow objects" is hard to follow.
    - "metadata/" -> the discussion on URI schemes is hard to follow, again an example would help.
    - "Retrospective provenance Profile" -> is the production of wfdesc / wfprov RDF data automatic or manual ?
Practical realisation of CWLProv
    - Figure 5 -> what does "relativised job object"
    - Figure 5 -> which steps are the most costly (time/space)

CWLProv evaluation
    - CWLProv supports syntactic, semantic, and pragmatic -> since pragmatic refers to scientific context / claims, etc., it is unclear how pragmatic interoperability is addressed.
    - Why choosing these 3 bioinformatics workflows, do they cover different aspects of the evaluation ? May be a single in-depth description would be enough.
    - "In addition, the resource requirement" -> this is a good example for R19, a link to R19 would be useful here.
    - The re-enactment scenario is clear as well as the provenance queries scenarios but the interoperability evaluation is less clear towards the "pragmatic assumption" and domain annotations.
    - Temporal and spatial overhead -> For the RNAseq and Alignment workflows, the Prov overhead appears as quite noticable. Which part of the process (Fig. 5) would explain this difference ?
Discussion and Future Directions
    - Even if it could be completed as mentioned before, the discussion is well developed and very interesting.
    - With respect to "big omics data", the scalability issue is real but interesting future directions are proposed.
    - "Selected jobs provenance" : this paragraph is a bit confusing since the lack of completeness of provenance was identified as a main issue, it highlights that this complete capture approach may raise human-tractability issues.
    - In addition, users can add domain-specific annotations to data -> How ? how difficult/easy it is ?
Access logs to the supporting materials :
The DOI seems OK but data.mendeley.com seems unavailable :
```
â€”2019-01-22 21:34:01â€”  https://data.mendeley.com/datasets/97hj93mkfd/3
Resolving data.mendeley.com… 54.154.101.245, 54.229.27.249
Connecting to data.mendeley.com|54.154.101.245|:443… failed: Operation timed out.
```
```
--2019-01-22 21:43:51--  https://doi.org/10.17632/xnwncxpw42.1
Resolving doi.org... 2606:4700:20::6819:af09, 2606:4700:20::6819:ae09, 104.25.175.9, ...
Connecting to doi.org|2606:4700:20::6819:af09|:443... connected.
HTTP request sent, awaiting response... 302
Location: https://data.mendeley.com/datasets/xnwncxpw42/1 [following]
--2019-01-22 21:43:51--  https://data.mendeley.com/datasets/xnwncxpw42/1
Resolving data.mendeley.com... 54.154.101.245, 54.229.27.249
Connecting to data.mendeley.com|54.154.101.245|:443... failed: Operation timed out.
Connecting to data.mendeley.com|54.229.27.249|:443... ^C
```

Methods

Are the methods appropriate to the aims of the study, are they well described, and are necessary controls included? Choose an item.

Conclusions

Are the conclusions adequately supported by the data shown? Choose an item.

Reporting Standards

Does the manuscript adhere to the journal's guidelines on minimum standards of reporting? Choose an item.

Choose an item.

Statistics

Are you able to assess all statistics in the manuscript, including the appropriateness of statistical tests used? Choose an item.

Quality of Written English

Please indicate the quality of language in the manuscript: Choose an item.

Declaration of Competing Interests

Please complete a declaration of competing interests, considering the following questions:

- Have you in the past five years received reimbursements, fees, funding, or salary from an organisation that may in any way gain or lose financially from the publication of this manuscript, either now or in the future?
- Do you hold any stocks or shares in an organisation that may in any way gain or lose financially from the publication of this manuscript, either now or in the future?
- Do you hold or are you currently applying for any patents relating to the content of the manuscript?
- Have you received reimbursements, fees, funding, or salary from an organization that holds or has applied for patents relating to the content of the manuscript?
- Do you have any other financial competing interests?
- Do you have any non-financial competing interests in relation to this paper?

If you can answer no to all of the above, write 'I declare that I have no competing interests' below. If your reply is yes to any, please give details below.

I declare that I have no competing interests.

I agree to the open peer review policy of the journal. I understand that my name will be included on my report to the authors and, if the manuscript is accepted for publication, my named report including any attachments I upload will be posted on the website along with the authors' responses. I agree for my report to be made available under an Open Access Creative Commons CC-BY license (http://creativecommons.org/licenses/by/4.0/). I understand that any comments which I do not wish to be included in my named report can be included as confidential comments to the editors, which will not be published.

Choose an item.

To further support our reviewers, we have joined with Publons, where you can gain additional credit to further highlight your hard work (see: https://publons.com/journal/530/gigascience). On publication of this paper, your review will be automatically added to Publons, you can then choose whether or not to claim your Publons credit. I understand this statement.

Yes Choose an item.
