# Supplementary material for: Sharing interoperable workflow provenance: A review of best practices and their practical application in CWLProv
Source: Gigascience. 2019 Nov 1;8(11):giz095. doi: 10.1093/gigascience/giz095 (PMC6824458; doi:10.1093/gigascience/giz095)
Supplement: giz095_Reviewer_2_Report_Revision_1 — Alban Gaignard -- 6/24/2019 Reviewed [file giz095_reviewer_2_report_revision_1.pdf]

Reviewer Report

Title: Sharing interoperable workflow provenance: A review of best practices and their practical application in CWLProv

Version: Revision 1 **Date:** 6/24/2019

Reviewer name: Alban Gaignard

Reviewer Comments to Author:

I thank the authors for taking into account all the requested / highlithed needs for clarification.
I particularly appreciated the graphical clustering or requirements, the box describing the BagIt approach, the bridges with Linked Data efforts as well as the quality of the discussion and future works section.
The response to reviewers is concise and appropriate.
In conclusion, a very nice research work, supported with consequent software development efforts.
This work opens very interesting perspectives towards better reuse, transparency, etc. in data driven sciences.

Methods

Are the methods appropriate to the aims of the study, are they well described, and are necessary controls included? Choose an item.

Conclusions

Are the conclusions adequately supported by the data shown? Choose an item.

Reporting Standards

Does the manuscript adhere to the journal's guidelines on minimum standards of reporting? Choose an item.

Choose an item.

Statistics

Are you able to assess all statistics in the manuscript, including the appropriateness of statistical tests used? Choose an item.

Quality of Written English

Please indicate the quality of language in the manuscript: Choose an item.

Declaration of Competing Interests

Please complete a declaration of competing interests, considering the following questions:

- Have you in the past five years received reimbursements, fees, funding, or salary from an organisation that may in any way gain or lose financially from the publication of this manuscript, either now or in the future?
- Do you hold any stocks or shares in an organisation that may in any way gain or lose financially from the publication of this manuscript, either now or in the future?
- Do you hold or are you currently applying for any patents relating to the content of the manuscript?
- Have you received reimbursements, fees, funding, or salary from an organization that holds or has applied for patents relating to the content of the manuscript?
- Do you have any other financial competing interests?
- Do you have any non-financial competing interests in relation to this paper?

If you can answer no to all of the above, write 'I declare that I have no competing interests' below. If your reply is yes to any, please give details below.

I declare that I have no competing interests.

I agree to the open peer review policy of the journal. I understand that my name will be included on my report to the authors and, if the manuscript is accepted for publication, my named report including any attachments I upload will be posted on the website along with the authors' responses. I agree for my report to be made available under an Open Access Creative Commons CC-BY license (http://creativecommons.org/licenses/by/4.0/). I understand that any comments which I do not wish to be included in my named report can be included as confidential comments to the editors, which will not be published.

Choose an item.

To further support our reviewers, we have joined with Publons, where you can gain additional credit to further highlight your hard work (see: https://publons.com/journal/530/gigascience). On publication of this paper, your review will be automatically added to Publons, you can then choose whether or not to claim your Publons credit. I understand this statement.

Yes Choose an item.
